# Comparison of American mink embryonic stem and induced pluripotent stem cell transcriptomes

**DOI:** 10.1186/1471-2164-16-S13-S6

**Published:** 2015-12-16

**Authors:** Aleksei G Menzorov, Natalia M Matveeva, Marios N Markakis, Venyamin S Fishman, Knud Christensen, Anna A Khabarova, Inna E Pristyazhnyuk, Elena A Kizilova, Susanna Cirera, Razvan Anistoroaei, Oleg L Serov

**Affiliations:** 1 Laboratory of Developmental Genetics, Federal State Budget Scientific Institution "The Federal Research Center Institute of Cytology and Genetics of Siberian Branch of the Russian Academy of Sciences", Novosibirsk, 630090, Russia; 2 Natural Sciences Department, Novosibirsk State University, Novosibirsk, 630090, Russia; 3 Department of Biology, Plant Growth and Development, University of Antwerp, 2020 Antwerp, Belgium; 4 Section of Genetics, Bioinformatics and Systems Biology, Department of Veterinary Clinical and Animal Sciences, Faculty of Health and Medical Sciences, University of Copenhagen, 1870 Frederiksberg C, Denmark

**Keywords:** pluripotency, reprogramming, American mink, iPS cells, ES cells, Nanog, transcriptome

## Abstract

**Background:**

Recently fibroblasts of many mammalian species have been reprogrammed to pluripotent state using overexpression of several transcription factors. This technology allows production of induced pluripotent stem (iPS) cells with properties similar to embryonic stem (ES) cells. The completeness of reprogramming process is well studied in such species as mouse and human but there is not enough data on other species. We produced American mink (*Neovison vison*) ES and iPS cells and compared these cells using transcriptome analysis.

**Results:**

We report the generation of 10 mink ES and 22 iPS cell lines. The majority of the analyzed cell lines had normal diploid chromosome number. The only ES cell line with XX chromosome set had both X-chromosomes in active state that is characteristic of pluripotent cells. The pluripotency of ES and iPS cell lines was confirmed by formation of teratomas with cell types representing all three germ layers. Transcriptome analysis of mink embryonic fibroblasts (EF), two ES and two iPS cell lines allowed us to identify 11831 assembled contigs which were annotated. These led to a number of 6891 unique genes. Of these 3201 were differentially expressed between mink EF and ES cells. We analyzed expression levels of these genes in iPS cell lines. This allowed us to show that 80% of genes were correctly reprogrammed in iPS cells, whereas approximately 6% had an intermediate expression pattern, about 7% were not reprogrammed and about 5% had a "novel" expression pattern. We observed expression of pluripotency marker genes such as *Oct4*, *Sox2 *and *Rex1 *in ES and iPS cell lines with notable exception of *Nanog*.

**Conclusions:**

We had produced and characterized American mink ES and iPS cells. These cells were pluripotent by a number of criteria and iPS cells exhibited effective reprogramming. Interestingly, we had showed lack of *Nanog *expression and consider it as a species-specific feature.

## Background

Recently mouse and human adult and embryonic fibroblasts (EF) have been reprogrammed into pluripotent state by overexpression of only four transcription factors [1-3]. At present, induced pluripotent stem (iPS) cells have been derived from somatic cells of mammalian species such as primates [4-7], rat [8], prairie vole [9], rabbit [10], dog [11-16], pig [17], horse [18], sheep [19], cow [20], goat [21] and buffalo [22]. In addition, iPS cells have been produced from endangered species, e.g. rhinoceros [7] and snow leopard [23]. Mouse and human iPS cells are very similar to embryonic stem (ES) cells produced from blastocyst inner cell mass [24]. Apart from extensively studied mouse and human iPS cells, little is known about characteristics of iPS cells of other mammalian species. Additionally, ES cells are produced from a limited number of species and their pluripotent state is rarely well characterized. This complicates comparison between ES and iPS cells. So far, there are no reports of iPS cell derivation from somatic cells of any mustelid species. The aim of the current research is producing mink ES and iPS cells to assess reprogramming completeness by comparing gene expression profiles of mink EF, ES and iPS cells.

We report generation of American mink pluripotent ES and iPS cells. Transcriptome analysis shows efficient EF genome reprogramming. Pluripotent stem cells express key pluripotency markers with notable exception of *Nanog*.

## Results

### ES and iPS cell derivation

American mink ES cell line MES12 used in this study was previously described by our group [25]. By using the same protocol we have generated an additional set of mink ES cell lines. Twelve mink morulas and early blastocysts were plated on feeder cells, and in a total of 10 ES cell lines were produced. As in the previous series, they were designated as MES (MES20 - MES29). Unlike mouse ES cells, mink ES cells contain peripheral visible granules and form flat monolayer colonies of epithelial-like morphology [25,26].

**Figure 1:**
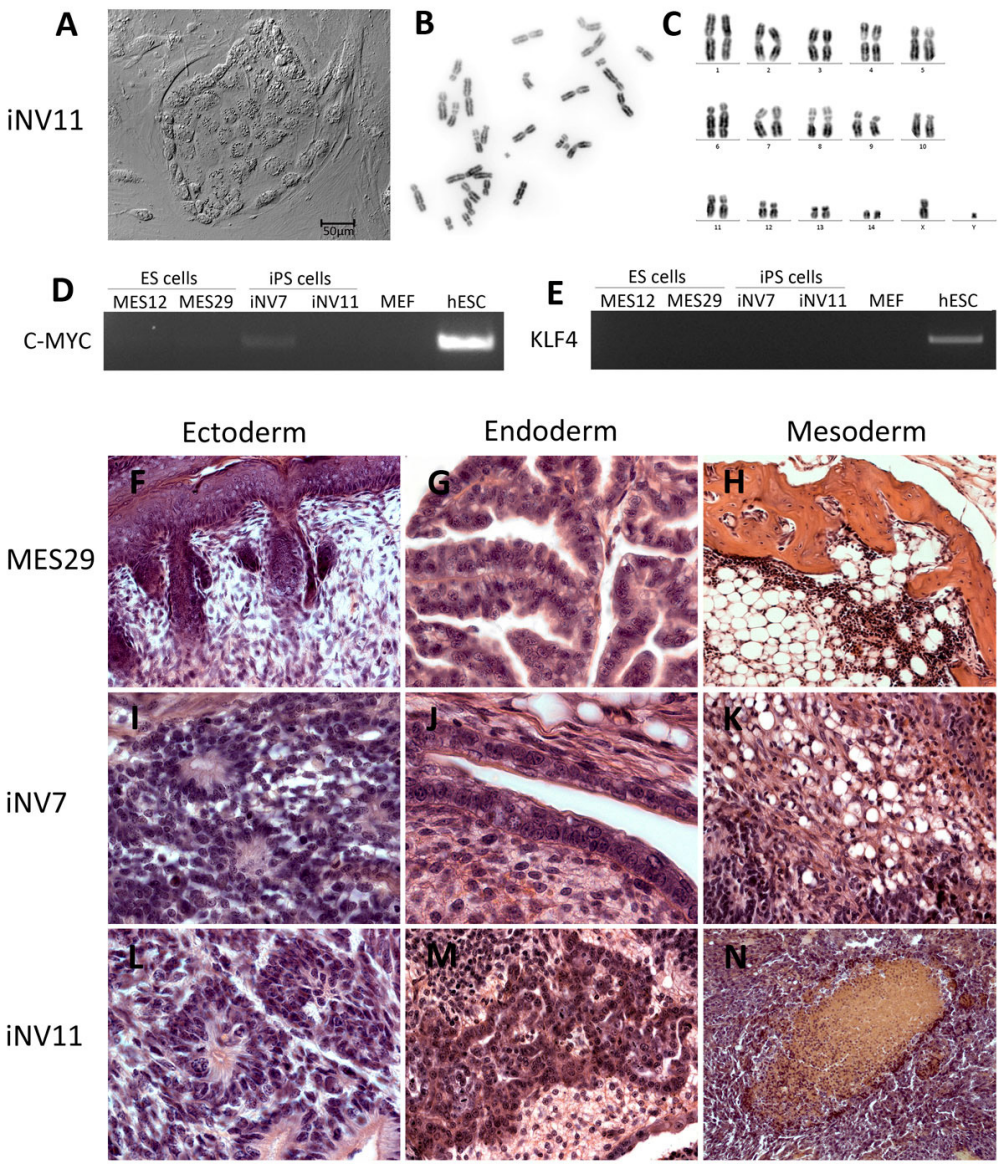
**iPS cell morphology, karyotype and examples of cell types in teratomas developed from mink ES and iPS cells**. **a **- morphology of iNV11 iPS cell line colony; **b **- iNV11 metaphase plate; **c **- iNV11 karyotype; **d **- expression of human transgene *C-MYC*; **e **- expression of human transgene *KLF4*; examples of representative cell types in teratomas formed from pluripotent cell lines: **f **- epidermal epithelium with hair follicle; **g **- gut-like epithelium; **h **- bone, adipose and hematopoietic tissues; **i **- rosettes of neural epithelium; **j **- gut-like epithelium; **k **- connective and adipose tissues; **l **- rosettes of neural epithelium; **m **- hepatocytes; **n **- erythropoietic tissue.

To produce iPS cells from primary mink EF we used the following human reprogramming transcription factors: OCT4, SOX2, C-MYC and KLF4. To facilitate reprogramming process we supplemented culture medium with valproic acid, an epigenetic modifier that inhibits histone deacetylases. Two weeks after first lentiviral transduction of EF we observed many colonies with different morphology. Based on mink ES-like morphology we selected and picked up 25 colonies. From these primary colonies 22 iPS cell lines were successfully produced. We designated them as iNV (iNV1 - iNV22). Morphology of iPS cell line iNV11 colony is shown in Figure 1a.

We assessed the efficacy of colony formation in a separate experiment with the same conditions as above. We observed 722 colonies (1.2% of initial 6 × 10^4 ^transduced cells) on day 11 after transduction of mink EF. Most of them had mink ES-like phenotype.

### Cytogenetic analysis

We randomly selected 6 out of 10 ES cell lines and 11 out of 22 iPS cell lines for the analysis. The results of the cytogenetic analysis are shown in Table 1. Five ES cell lines were XY, and MES25 was XX. All analyzed iPS cell lines were XY as they were produced from EF derived from a single male embryo. All analyzed pluripotent cell lines with the exception of MES20 had diploid modal chromosome number. We consider it as an indication of karyotype stability. In MES20 64.3% of cells had tetraploid chromosome number. Tetraploidization of pluripotent cells frequently happens *in vitro *for human and mouse ES cells and thus some percentage of tetraploid cell lines could be considered as normal [27]. A metaphase plate and a karyotype of iNV11 are presented in Figure 1b-c.

**Table 1:** Cytogenetic analysis of ES and iPS cell lines.

Cell line	Sex chromosomes	29 chromosomes (%)	30 chromosomes (%)	31-32 chromosomes (%)	Tetraploid cells (%)	N
MES20	XY	4,8	23,8		**71,4**	21
MES22	XY	17.6	**50.0**	14.7	17.6	34
MES24	XY	12.0	**68.0**	12.0	8.0	25
MES25	XX	9.4	**84.4**		6.2	32
MES27	XY	6.7	**79.9**	6.7	6.7	30
MES29	XY	14.3	**74.3**	5.7	5.7	35
iNV3	XY	21.9	**75.0**	3.1		32
iNV5	XY	7.1	**85.8**		7.1	28
iNV6	XY	21.0	**67.9**	3.6	7.1	28
iNV7	XY		**87.1**	3.2	9.7	31
iNV9	XY	12.1	**78.8**		9.1	33
iNV11	XY	15.1	**72.7**	3.1	9.1	33
iNV13	XY	16.1	**80.6**		3.2	31
iNV15	XY	9.4	**81.3**	3.1	6.2	32
iNV18	XY	11.5	**77.0**	3.8	7.7	26
iNV19	XY	12.0	**80.0**		8.0	25
iNV20	XY	9.1	**60.6**	8.8	20.6	33

N - number of metaphase plates, bold font - percentage of cells with modal chromosome number

### X-inactivation analysis

Female pluripotent cells have both X-chromosomes in active state. One of the markers of inactive X-linked chromatin modifications is histone H3 trimethylated at lysine 27 (H3K27me3) [28,29]. Control female mink EF had 82.3% (n = 96) cells positive for H3K27me3 as expected. In the only XX pluripotent cell line, MES25, 87% (n = 285) of the cells were negative for inactive X-linked chromatin marker. Most of the positive cells were situated at the borders of ES cell colonies and might represent differentiation (Additional file 1). Thus, MES25 has both X-chromosomes in active state confirming its pluripotency.

### Generation and analysis of teratomas

To analyze the pluripotency of ES and iPS cells we used the *in vivo *teratoma formation assay. The summary of teratoma analysis data is presented in Table 2. Cell types in the individual teratomas are presented in Additional file 2. Teratomas generated from ES cell lines MES22, MES24 and MES29, and iPS cell lines iNV7, iNV11, iNV19 and iNV20 had most or all analyzed cell types representing three germ layers (ectoderm, endoderm and mesoderm). Thus, we consider these cell lines pluripotent. Representative cell types in MES29, iNV7 and iNV11 derived teratomas are shown in Figure 1f-n. These three cell lines and the previously characterized MES12 [25] were chosen for the transcriptome analysis.

**Table 2:** Summary of histological analysis of teratomas formed after injection of ES and iPS cells into immunodeficient mice.

	iNV3	iNV5	iNV6	iNV7	iNV11	iNV13	iNV15	iNV19	iNV20	MES20	MES22	MES24	MES29
primitive neuroepithelium		**+**		**+**	**+**	**+**	**+**	**+**	**+**		**+**	**+**	**+**
keratinized epithelium		**+**	**+**	**+**		**+**		**+**	**+**	**+**	**+**	**+**	**+**
melanocytes and pigmented epithelium													**+**
connective tissue	**+**	**+**	**+**	**+**	**+**	**+**	**+**	**+**	**+**	**+**	**+**	**+**	**+**
cartilage				**+**					**+**		**+**	**+**	**+**
bone	**+**		**+**	**+**	**+**	**+**		**+**	**+**	**+**	**+**	**+**	**+**
muscle	**+**	**+**	**+**	**+**	**+**	**+**		**+**	**+**			**+**	**+**
adipose tissue	**+**	**+**	**+**	**+**	**+**		**+**	**+**	**+**	**+**	**+**	**+**	**+**
erythropoietic tissue	**+**	**+**	**+**	**+**	**+**		**+**	**+**	**+**		**+**	**+**	**+**
lymphoid tissue	**+**		**+**	**+**	**+**			**+**	**+**	**+**	**+**	**+**	**+**
ciliated epithelium					**+**			**+**	**+**		**+**	**+**	**+**
gut-like epithelium				**+**	**+**			**+**	**+**		**+**	**+**	**+**
glandular epithelium			**+**	**+**				**+**	**+**		**+**	**+**	**+**
hepatocytes or pancreocytes		**+**		**+**	**+**	**+**		**+**	**+**		**+**	**+**	**+**

### Transgene silencing analysis

Transgenes inserted into pluripotent stem cells with viral vectors are usually silenced [28,30,31]. We performed analysis of the human transgene silencing in iPS cell lines iNV7 and iNV11 by RT-PCR using human specific primers (Additional file 3) for *OCT4*, *C-MYC*, *KLF4 *and *SOX2*. Human transgenes *KLF4 *and *C-MYC *were silenced, as no product was visible in the agarose gel with the exception of a slight band in the iNV7 line for *C-MYC *(Figure 1d-e). In addition, we showed *C-MYC *and *KLF4 *transgene silencing for iPS cell lines iNV5, iNV15 and iNV20 (data not shown). In the case of *SOX2 *and *OCT4 *genes, due to their high sequence conservation between human and mink in the coding region, we were not able to design human specific primers. Therefore, we have no information about the silencing of the *SOX2 *and *OCT4 *transgenes. We can thus conclude that the human transgenes are mostly silenced in the mink iPS cell lines.

### Quantitative real-time PCR (qPCR) results

We verified expression of several mink genes by qPCR (Additional file 4 Additional file 5). We found significant differences between the five cell lines for the gene expression: *Oct4 *(*P *= 2.00E-08), *Nanog *(*P *= 3.00E-08), *Gdf3 *(*P *= 8.00E-08), *Sox2 *(*P *= 1.63E-05) and *Nestin *(*P *= 0.0351). Interestingly, looking at the pairwise post comparisons (Additional file 6 Figure 2a), we can see that gene expression varies between pluripotent cell lines. As expected, the mink EF show a pattern of expression very different from the pluripotent cell lines.

**Figure 2:**
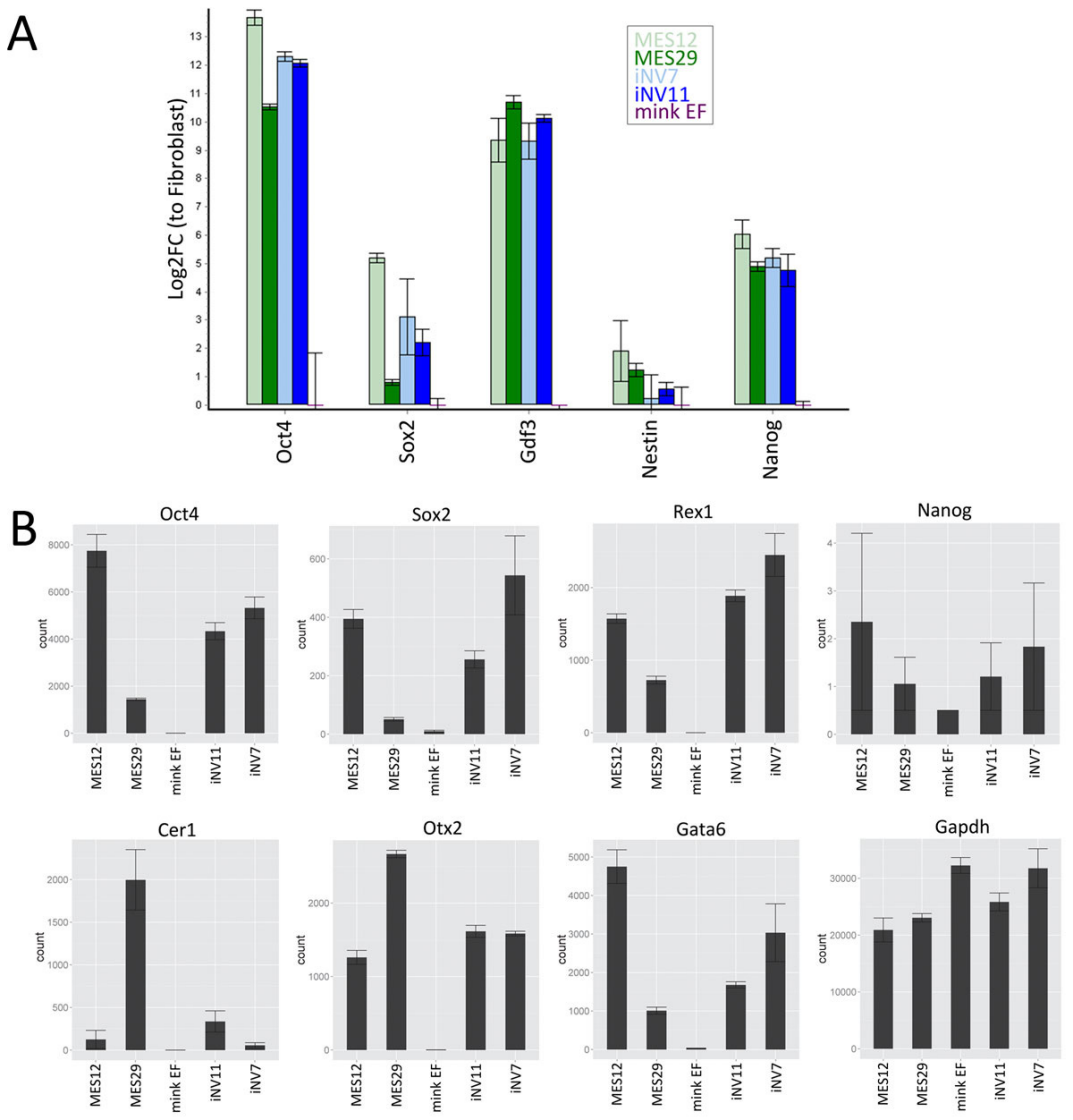
**Expression levels of several genes of interest in mink EF, ES and iPS cells based on transcriptome analysis and qPCR**. **a **- results of qPCR for *Oct4*, *Sox2*, *Gdf3*, *Nestin *(*Nes*) and *Nanog*; **b**- expression levels of *Oct4*, *Sox2*, *Rex1*, *Nanog*, *Cer1*, *Otx1*, *Gata6 *and *Gapdh *genes in mink pluripotent cells and EF. The vertical axis represents counts determined for each sample by transcriptome analysis.

### Transcriptome analysis

To assess completeness of reprogramming in mink iPS cells we compared transcriptomes of ES cells, iPS cells and EF. We selected cell lines for transcriptome analysis based on cytogenetic analysis and teratoma formation test. For transcriptome analysis all cell lines were cultured for two passages in three independent replicates. Mink EF were collected on passage 7, ES cells lines MES12 and MES29 on passages 21 and 25, respectively, iPS cell lines iNV7 and iNV11 on passages 10 and 11, respectively. To eliminate contamination with mink EF transcripts ES and iPS cells were grown on mouse strain CD-1 feeder cells. We produced between 4.0 and 23.9 million reads for each cell line replicate (see Additional file 7).

**Figure 3:**
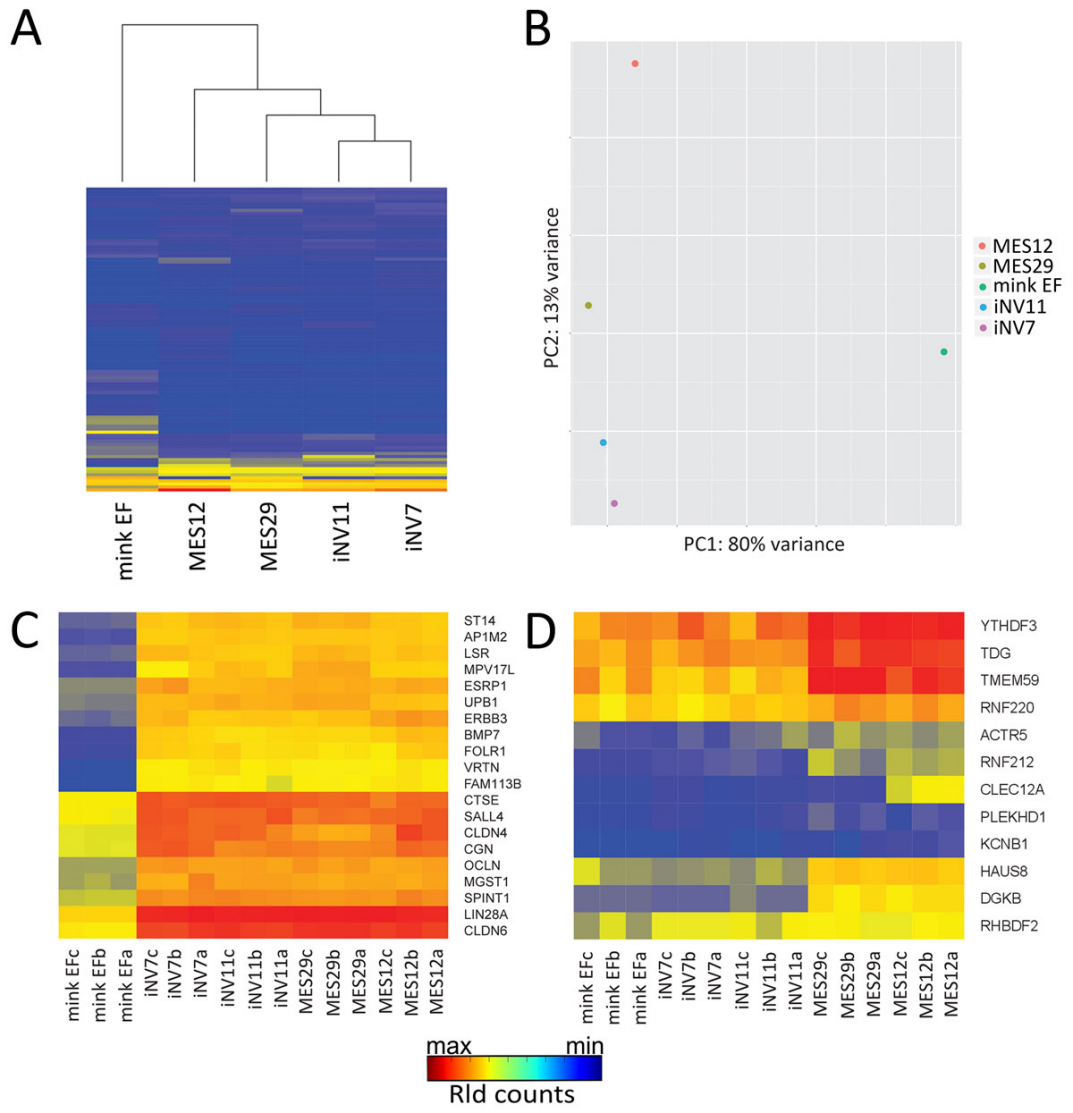
**Transcriptome gene expression analysis**. **a **- hierarchical cluster dendrogram based on the expression levels of 100 genes with a highest variance between samples; **b **- principal component analysis of expression based on the same set of genes as in **a**. The horizontal axis represents the first principal component of variation - a projection of the array data representing the maximal variance among them (80% of total variance between samples). The vertical axis represents the second principal component of variation (13% of total variance); **c **- example of heat-map presentation of gene expression profiles of correctly reprogrammed genes in mink EF, ES and iPS cells. Each row of the heat-map corresponds to one gene, and expression levels are shown using color-code (blue color represents the lowest expression level, red color - the highest); **d **- example of heat-map presentation of gene expression profiles of not reprogrammed genes in mink EF, ES and iPS cells.

Hierarchical clustering, as well as principal component analysis (PCA) of transcriptome data, shows a sharp contrast between EF and pluripotent cells (Figure 3a-b). 80% of all differences in expression levels observed between cell lines can be explained by difference between differentiated and pluripotent cells, whereas only 13% of dissimilarities reflect difference between ES and iPS cells (Figure 3b). This result clearly indicates that the reprogramming process results in cells that lose most of EF signature genes and are very similar to the pluripotent ES cells.

The observed slight difference between ES and iPS cells might reflect incomplete reprogramming, a phenomenon well known for mouse and human iPS cells. A common source of incomplete reprogramming is so-called "somatic memory" determined as expression of certain genes in iPS cells on a level of original somatic cells [32]. To study "somatic memory" in iPS cells we decided to perform detailed analysis of genes that are differently expressed in EF and ES cells. We annotated 11831 contigs out of 30490, generated by Trinity. Among them we identified 6891 unique genes and found 3201 showing significant difference in expression between EF and either MES12 or MES29 cells (Additional file 8). Of these, the majority of genes were expressed at the same level in iPS and ES cells, thus being correctly reprogrammed. List and examples of such genes are presented in Additional file 9, Figure 2b (*Oct4*, *Sox2 *and *Rex1*) and Figure 3c. Some genes show intermediate expression pattern, i.e. characterized by expression level between EF and ES cells. A small number of genes were not reprogrammed, displaying EF-like expression pattern in iPS cells with expression level significantly different from both MES12 and MES29 cell lines (Figure 3)d. Finally, few genes do not fall into correctly reprogrammed, intermediate or not reprogrammed categories. We designed these genes as "novel", since they show a novel expression pattern in iPS cells, different from both EF and ES cells (Additional file 10). In contrast to genes from intermediate category, the expression level of these "novel" genes does not lie between levels of EF and ES cells, suggesting that unexpected expression of these genes is not due to incomplete activation or repression of respective promoters during reprogramming process, but rather is a result of some reprogramming mistakes. The lists and number of genes with different patterns of reprogramming are presented in Additional file 8 and Table 3.

**Table 3:** Percentage of different categories of genes in mink iPS cells.

	Correctly reprogrammed	Intermediate pattern	Not reprogrammed	"Novel" pattern
iNV7	2569 (80.26%)	213 (6.65%)	239 (7.47%)	180 (5.62%)
iNV11	2579 (80.57%)	223 (6.97%)	224 (7.00%)	175 (5.47%)
Shared between iNV7 and iNV11	2232 (69.73%)	105 (3.28%)	90 (2.81%)	66 (2.06%)

## Discussion

We have produced American mink ES and iPS cell lines. Their pluripotency was confirmed by teratoma formation test. We have observed silencing of two of four transgenes in analyzed iPS cell lines. That is in line with reports on transgene silencing in mouse and human iPS cells [28,30,31]. American mink represents one of a few species for which both ES and iPS cells were obtained. This had allowed us to address several important questions. One of them is completeness of somatic cell reprogramming, whether genes of EF origin are effectively reprogrammed to pluripotent state in iPS cells. Another question refers to the nature of the pluripotent state in the American mink ES and iPS cells, naïve or primed [33,34].

We had shown that the majority of genes were correctly reprogrammed (Table 3). About 400 genes were not reprogrammed or have "novel" expression pattern in iPS cells. That is comparable with data on human iPS cells where approximately 600 genes were not reprogrammed in iPS cells compared to ES cells [32]. In another study, it was shown that in 12 human iPS cell lines an average of 550 genes were not reprogrammed compared to ES cell reference. It should be noted that ES cell lines differed in gene expression. In the same study an analysis of 20 ES cell lines revealed on average 387 differentially expressed genes in each line compared to ES cell reference [24]. Thus, such number of differentially expressed genes could be attributed to "normal" gene expression fluctuations or differences. In general, we conclude that the majority of the genes in both analyzed iPS cell lines were effectively reprogrammed.

The second question concerns the status of pluripotency in mink cells. Recently, pluripotent cells were divided into two categories: naïve and primed, distinguished by colony morphology, pattern of gene expression and other features [33,34]. Mouse ES cells are considered naïve while human ES cells resemble primed epiblast stem cells. The appliance of 2i inhibitors (MEK inhibitor PD 0325901 and GSK3 inhibitor CHIR 99021) was shown to switch the pluripotent state from primed to naïve. Among species with reported iPS cell derivation the closest to *N. vison *is dog, *Canis familiaris*, order Carnivora. Interestingly, in canine ES [35-39] and iPS cells [11-16] different research groups were able to observe both morphological cell types, although their pluripotent state was not identified. However, it should be noted that only three research groups were able to produce teratomas with all three germ layer derivatives from canine ES cells [38] and iPS cells [15,16]. Lack of teratoma formation raise a question about pluripotency of these cell lines. In our study, ES and iPS cells are pluripotent by this criterion. Both types of the cells form morphologically monolayer colonies. In mouse and human pluripotent stem cells, this morphology is characteristic of primed pluripotent state. However, the morphology of mink ES and iPS cells is very similar to that of mink blastocyst inner cell mass [26]. This characteristic could be species-specific and does not point out a pluripotent state.

Expression of pluripotency-associated genes was assessed based on transcriptome data showing that both mink ES and iPS cells represent one cell type. Moreover, we observed expression of several known key pluripotency genes, including *Rex1 *(Figure 2b), which is considered a marker of naïve pluripotent state [40]. One notable exception is the lack of *Nanog *expression as only several copies of its transcript were found in solitary replicates of pluripotent cell lines. This finding was also verified by qPCR. Taking into consideration the proven pluripotency of analyzed cell lines, we presume that the low level of *Nanog *expression is a species-specific feature. Interestingly, *Gata6 *is expressed in all four pluripotent cell lines. *Gata6 *is *Nanog*'s antagonist in mouse early embryo development, Nanog-positive cells tend to form epiblast while Gata6-positive - primitive endoderm [41]. Thus, coexpression of *Rex1* and *Nanog *could be considered contradictory.

We have also analyzed the expression of several genes such as *Cer1 *[42] and *Otx2 *[43] that are characteristic of mouse primed pluripotent cells (Figure 2b). We can observe the differences in these genes expression between the different ES cell lines as well as when compared to the iPS cell lines. Interestingly, the comparative levels of *Oct4*, *Sox2 *and *Rex1 *expression in MES12 and MES29 are reciprocal to *Cer1 *and *Otx2*. It might point out to different pluripotent states of these ES cell lines. Nevertheless, based on these gene expression levels we cannot assess the pluripotent state of the analyzed cell lines. It was shown that in mouse these genes are expressed both in ES and epiblast stem cells but on different levels [42,44]. Due to the fact that we do not have a control with known pluripotency status the expression itself is not an indicator.

As it was shown for various mouse pluripotent cell lines, addition of 2i could shift primed cells into naïve [33,34]. Interestingly, to produce and culture canine pluripotent cells investigators used supplementation with substantially different factors, e.g. LIF as used for mouse ES cells with bFGF as for human ES cells [12,14,15,38]. In addition, some groups were able to obtain pluripotent cells using 2i + LIF + bFGF [16] and LIF + bFGF + 2i + valproic acid + TGH-β antagonist A83-01 [11]. Some researchers used mix of all mentioned factors for iPS cell production but cultured iPS cells with LIF only [13]. To test whether the change of culture condition could change morphology of mink iPS colonies we applied various combinations: 2i, (2i + LIF), (2i + bFGF) and (2i + LIF + bFGF) respectively to iNV11 cells for two weeks. The morphology of the colonies remained unchanged. If mink iPS cells are in primed pluripotent state, it maybe that additional factors are needed to shift it to naïve. Alternatively, they could already be in naïve state as indicated by *Rex1 *expression.

## Conclusions

We produced and characterized American mink ES and iPS cells. These cell lines have diploid chromosome number, and are pluripotent based on teratoma formation test. The transcriptome analysis shows efficient reprogramming of the mink EF genome to the pluripotent state in iPS cells. Colony morphology and expression of several marker genes are not enough to conclude whether the cells are in naïve or primed pluripotent state. We have found that *Nanog *is nearly absent in these pluripotent stem cells and consider it as species-specific feature.

## Methods

### Production of mink embryonic fibroblasts

Primary EF of American mink were obtained from individual 29-day embryos by standard protocol [45]. Mink of wild type genotype were used as donors of embryos. The EF culture medium consisted of DMEM (Invitrogen, USA) supplemented with 10% fetal bovine serum (Invitrogen, USA), and 1x penicillin and streptomycin (Invitrogen, USA).

### Production of mink ES cell lines

To produce mink ES cells, the previously published protocol was followed [25]. Embryos were obtained from Public Center "Fur-bearing and farm animals" of Federal State Budget Scientific Institution "The Federal Research Center Institute of Cytology and Genetics of Siberian Branch of the Russian Academy of Sciences" (ICG SB RAS), Novosibirsk, Russia. Briefly, embryos of standard (wild type) genotypes at morula and early blastocyst stage were plated on plastic dishes coated with 0.1% gelatin on mitomycin C inactivated mink EF. *Zona pellucidae *of embryos was previously removed by treatment in 0.5% pronase solution. Within a few days the embryos attached to the feeder layer of EF and formed colonies of morphologically homogeneous cells similar to the ICM cells. These primary colonies were passaged by trypsinization with 0.25% Trypsin-EDTA (Invitrogen, USA) on the fresh feeder. ES cell culture medium contained α-MEM (Invitrogen, USA) with 20% ES cell qualified FBS (Invitrogen, USA), 1x NEAA (Invitrogen, USA), 1x GlutaMAX (Invitrogen, USA), 0.1 mM β-mercaptoethanol (Sigma, USA) and 1x Penicillin-Streptomycin (Invitrogen, USA). For subsequent culture we used 15% ES cell qualified FBS.

### Production of mink iPS cell lines

To produce iPS cells from the mink EF we used lentiviral vectors LeGO (http://www.lentigo-vectors.de/vectors.htm) with *GFP *and human reprogramming transcription factors: *OCT4*, *SOX2*, *C-MY*C and *KLF4*, courtesy of Dr. Sergei L. Kiselev. Lentiviruses were produced in Phoenix cell line using Lipofectamine LTX (Invitrogen, USA) according to manufacturer's recommendations. Multiplicity of infection was estimated as 4.8 using GFP lentiviral vector. Mink EF (3 × 10^5 ^cells, 15 × 10^3 ^cells/cm^2^) plated the day before were transduced with viruses containing four reprogramming transcription factors and 4 µg/ml Polybrene. Transduction was performed three consecutive days, on second and third day with halved amount of C-MYC lentivirus. Until day 10 the medium was changed daily with addition of 1 mM valproic acid. On day 7 transduced cells were seeded onto 10 cm culture dishes on feeder in ES cell culture medium. From days 13 to 20 colonies with mink ES cell-like morphology were picked up and expanded. All cell cultures were maintained at 37°C and 5% CO_2_.

All animal studies were undertaken with prior approval from Interinstitutional Bioethical Committee of ICG SB RAS.

### Cytogenetic analysis

Cytogenetic analysis for ES was carried out on passages 7-23 and for iPS cell lines on passages 4-6. Preparation of metaphase chromosomes from ES cells was performed as previously described with minor modifications: cells were treated with hypotonic solution (0.56% KCl for 15 min) and after that fixated with methanol/acetic acid (3:1) solution [46]. For each cell line an average of 30 metaphase plates were analyzed on Carl Zeiss Axioscop 2 imaging microscope with CoolCube 1 CCD-camera (Meta Sistems). Digital images were analyzed using ISIS (In Situ Imaging System, MetaSystems GmbH) software in collective Microscopic Center of ICG SB RAS, Novosibirsk, Russia.

### Immunofluorescence analysis of X-chromosome inactivation

Immunofluorescence analysis was carried out according to previously published protocol [47]. In short, cells cultured on glass coverslips were fixed with 3% formaldehyde (Fluka, Germany) and permeabilized with 0.1% Triton X-100 (Fluka, Germany). Non-specific binding was blocked with 2% bovine serum albumin (Sigma, USA) and cells were incubated with rabbit polyclonal or anti-H3K27me3 antibodies (1:500) (Molecular Probes, USA). The primary antibodies were visualized with the secondary goat anti-rabbit IgG antibodies conjugated with Alexa Fluor 488 (1:400) (Molecular Probes, USA). The cells were stained with DAPI, mounted in a glycerol solution containing 1,4 diazobicyclo-[2.2.3] octane (DABCO) (Sigma, USA) and visualized on LSM 780 NLO (Zeiss) based on AxioObserver Z1 (Zeiss) using ZEN software in collective Microscopic Center of ICG SB RAS, Novosibirsk.

### Teratoma formation analysis

For teratoma formation, we used SCID hairless outbred mice (Crl:SHO-Prkdc^scid^Hr^hr^) of SPF status and BALB/c-nu. Experiments with SCID mice were performed in the Center for Genetic Resources of Laboratory Animals (RFMEFI61914X0005) at ICG SB RAS; BALB/c-nu mice were kept in the Vivarium for conventional animals at ICG SB RAS. Teratomas were produced using standard protocol [45]. Between 3 and 17.7 × 10^6 ^ES cells on passages 7-23 and 1.5 to 7 × 10^6 ^iPS cells on passages 5-14 were injected subcutaneously into immunodeficient mice. Teratomas generated by ES or iPS cells were dissected after 3-12 weeks and were fixed in Bouin solution. Paraffin sections were prepared according to standard protocol and were stained with histological dyes Picro-Mallory trichromica (04-021822), Masson trichromica (04-011802), P.T.A.H.-hematoxyline (04-060802), Luxol fast blue Krever Barrera (04-200812), Azan trichromica (04-001802), Picrofuchsin Van Gizon (04-030802) (Bio-Optica Milano S.P.A., Italy) and with hematoxylin-eosin. Images were analyzed on Carl Zeiss Axioscop 2+ microscope with AxioCam HRc CCD-camera. Digital images were taken using AxioVision software in collective Microscopic Center of ICG SB RAS.

All animal studies were undertaken with prior approval from Interinstitutional Bioethical Committee of ICG SB RAS.

### RNA isolation and cDNA synthesis

RNA was isolated from each sample using TriReagent^® ^(MRC Inc., USA) according to manufacturer's recommendations. We had three technical replicates for each cell line (a, b and c, resulting in 15 samples in total). Genomic DNA was removed using RNeasy MinElute Cleanup kit (Qiagen, Germany). Quantity and quality were assessed in a Nanodrop ND-1000 machine (Thermo Scientific). The RNA integrity was assessed by gel electrophoresis and by an Experion system (BioRad Laboratories) using the ExperionTM RNA StdSens analysis kit (BioRad, Sweden). Average RNA quality indicator values (RQI) were 9.7 for the American mink ES cells, 9.6 for iPS cells and 9.3 for EF. This RNA was used for RNAseq library preparation and for cDNA synthesis, gene silencing analysis and qPCR.

One microgram of DNase I treated total RNA was used for cDNA synthesis [48]. cDNA synthesis was done in duplicates for each RNA sample, thus resulting in 30 samples. All samples were diluted eight times before using in qPCR.

### Primer design

We used Primer3Plus software to design primers for the following mink genes (Additional file 3): *Sox2*, *Oct4*, *Gdf3*, *Nanog *and *Nestin*. For the reference genes *Gapdh *and *Hprt1 *mink-specific primers were from Rouvinen-Watt et al. [49].

Human-specific primers for *C-MYC *and *KLF4 *were from Mathew et al. [50]. For *OCT4 *and *SOX2 *primer design was based on the maximum number of mismatches between human and mink sequences.

### qPCR

qPCR was performed as described by Cirera et al. [51]. Briefly, PCR efficiency was calculated from the log-linear portion of the standard curve for each assay, which was done with dilution serials of a pool of cDNA from all the samples to test. Efficiencies between 80 and 110% were accepted. GeneEx Professional (MultiD) software was used for pre-processing the qPCR data. Normalisation was undertaken using the two stable reference genes according to GeNorm and NormFinder software [52,53], namely GAPDH and HPRT1. Subsequently, technical replicates were averaged and relative quantities (fold changes, FC) were calculated based in the less expressed sample for each assay. Prior to statistical analysis, FC were log2 transformed to ensure normal distribution. Transcriptional differences between cell lines were evaluated using analysis of variance (ANOVA). A post hoc test (Tukey-Kramer) was performed in order to see which pairwise comparisons were significant. *P *values below 0.05 were considered significant. Results are reported as mean of the Log2 of the FC. The qPCR experiments, as well as the data analysis, were all compliant with MIQE guidelines [54].

### RNA-Seq data processing, reference assembly and alignment

Prior to library preparation the RNA quality and integrity was assessed according to Illumina guidelines. Library preparation was done using the TruSeq^® ^Stranded mRNA sample preparation 96 rxn kit (Illumina™) following the low sample protocol according to manufacturer's recommendations. Briefly, approximately 2.5 µg of total RNA was diluted and purified using RNA purification beads targeting the poly-A tail of the mRNA and subsequently was fragmented by means of the enzymes provided in the kit. After the cDNA synthesis adenylation of 3' ends and ligation of the adaptors were performed. Adaptors were ligated in 12-plex formations, allowing the pooling of 12 samples after the PCR enrichment of the library. Subsequently, the library was quantified using PicoGreen^® ^dye (Life Technologies™) as described in the manufacturer's protocol. Thereafter 12 samples were pooled at equal concentrations to create the eight pools. In order to accurately quantify the concentration in nM of our pools, the Kapa SYBR^® ^FAST universal qPCR kit (Kapa Biosystems™) for Illunima™ sequencing was used to quantify the number of the amplifiable molecules in the pools and the Bioanalyzer^® ^machine (Agilent Technologies™) to determine the average fragment size of the pools. These measurements allowed optimizing the flow cell clustering and proceed with the Run. The samples were sequenced in five lanes of an Illumina HiSeq2000 sequencer, for 50 bp pair-end read.

Since mink pluripotent cells were maintained on mouse feeder cells raw reads produced by next generation sequencing might contain transcripts of mouse feeder origin. Considering this, we aligned all data produced from iPS and ES cells sequencing to mm10-based mouse transcriptome (obtained from Illumina iGenomes project, https://support.illumina.com/sequencing/sequencing_software/igenome.html) using bowtie2 with parameters "--no-unal --no-sq --no-hd --np 0 --rdg 5,1 --rfg 5,1 --score-min L,-6.1,0 -X 500 --mp 3,2". All concordantly aligned read-pairs were considered as of mouse origin and eliminated from the analysis.

We used filtered data from mink iPS and ES cells as well as raw data from mink fibroblasts to perform *de novo *transcriptome assembly. Transcriptome was assembled using Trinity [55] with default parameters. We blasted Trinity-produced data against mouse transcripts and manually checked all highly similar (similarity >= 97%) sequences to ensure absence of contamination.

Annotation of *de novo *assembled contigs was done by alignment coding sequences against the human RefSeq mRNA database (Homo Sapiens GRCh37.66.cdna; http://www.ensembl.org/Homo_sapiens). The contigs that did not aligned against the human RefSeq were then blasted against the *Mustela putorius furo *protein sequences (Ensemble Genes 81; http://www.ensembl.org/info/data/ftp/). The annotation was made only for sequences translating to minimum of 50 amino acids in length. For efficient assembly, analysis and annotation of the data, scripts developed in-house and described earlier were used [56].

To perform differential expression we used mouse-filtered mink iPS and ES cells data. To make it comparable with fibroblasts data we also performed filtering of fibroblasts reads aligning it to mouse transcriptome as described above.

Resulting filtered sequencing data was aligned to the transcriptome assembly using bowtie [57] with options "-aS -X 800 --offrate 1". Produced alignment results were passed to the eXpress software [58] to obtain bias-corrected counts. To analyze expression on gene-level counts of all contigs representing the same gene were summed using self-made python script. Obtained counts were analyzed using DESeq2 package, that included library size normalization, dispersions estimation, differential expression tests etc. [59].

### Availability of supporting data

The raw sequenced data sets supporting the results of this article are available in the NCBI BioProject repository, http://www.ncbi.nlm.nih.gov/bioproject/297393.

## Competing interests

The authors declare that they have no competing interests.

## Authors' contributions

AGM conceived the study, participated in cell culture, provided analysis and interpretations of the data and is the principal investigator of the project; NMM participated in cell culture and produced all mink ES cells; MNM carried out Illumina libraries preparation, sequence processing and analysis; VSF carried out sequence processing and transcriptome-based gene expression analysis; KC carried out bioinformatics tasks, sequence analysis and gene annotation; AAK participated in cell culture; IEP carried out cytogenetic and immunocytochemical analyses, participated in cell culture; EAK carried out work with animals and teratoma histochemical analysis; SC carried out RNA preparation, qPCR analysis and interpretation; RA carried out bioinformatics tasks, sequence analysis and gene annotation, project coordination in Western Europe; OLS carried out interpretations of the data and project coordination in Russia. AGM did most of the writing with contributions from all authors. All authors read and approved the final manuscript.

## Supplementary Material

Additional file 1 **Mink EF and MES25 stained with H3K27me3 antibodies and visualized with the secondary antibodies conjugated with Alexa Fluor 488, counterstained with DAPI**. a - mink EF; b - MES25. (Additional file 1.jpg) Click here for file

Additional file 2 Histological analysis of individual teratomas formed after injection of ES and iPS cells into immunodeficient mice. Click here for file

Additional file 3 Primers for qPCR and RT-PCR. Click here for file

Additional file 4 qPCR raw data of selected gene expression. Click here for file

Additional file 5 qPCR data of selected gene expression corrected by PCR efficiency and normalized to reference genes *Gapdh *and *Hprt1*. Click here for file

Additional file 6 *P *values of the pairwise comparison (Tukey-Kramer post-test) of selected gene expression. Click here for file

Additional file 7 Number of pair-end reads and base in mink cell lines. Click here for file

Additional file 8 Lists of differentially expressed genes between mink EF, ES and iPS cells. Click here for file

Additional file 9 **Expression levels of selected pluripotency-associated genes in mink EF, ES and iPS cells**. Vertical axis represents counts determined for each sample by transcriptome analysis. Click here for file

Additional file 10 **Expression levels of shared between iNV7 and iNV11 genes with "novel" expression pattern in EF, ES and iPS cells**. Vertical axis represents counts determined for each sample by transcriptome analysis. Click here for file
