# Supplementary figures and images for: Comparison of American mink embryonic stem and induced pluripotent stem cell transcriptomes
Source: BMC Genomics. 2015 Dec 16;16(Suppl 13):S6. doi: 10.1186/1471-2164-16-S13-S6 (PMC4686781; doi:10.1186/1471-2164-16-S13-S6)

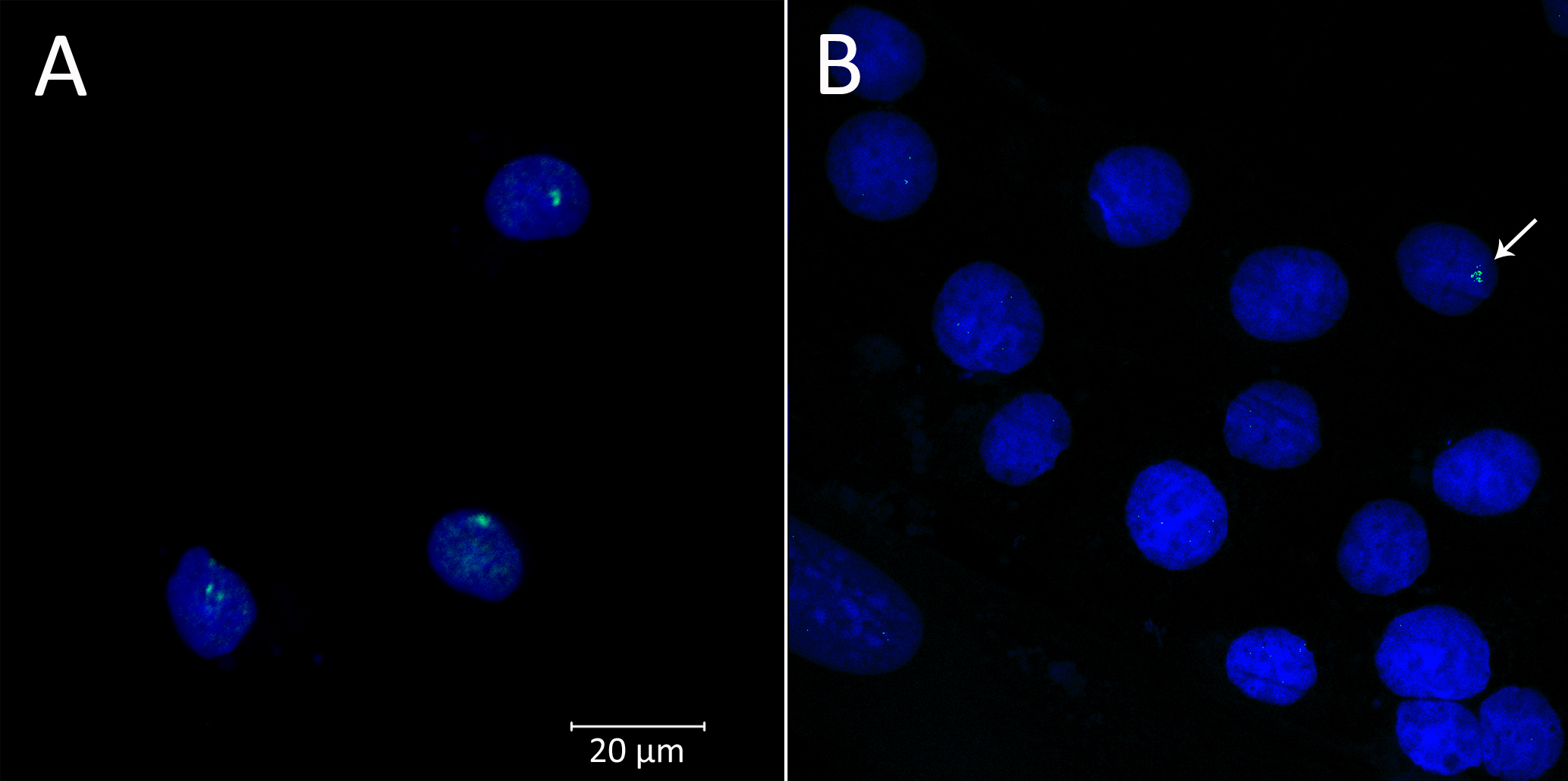

Supplement: Additional file 1 — Mink EF and MES25 stained with H3K27me3 antibodies and visualized with the secondary antibodies conjugated with Alexa Fluor 488, counterstained with DAPI. a - mink EF; b - MES25. (Additional file 1.jpg) [file 1471-2164-16-S13-S6-S1.jpg]
