# Supplementary material for: Comparison of American mink embryonic stem and induced pluripotent stem cell transcriptomes
Source: BMC Genomics. 2015 Dec 16;16(Suppl 13):S6. doi: 10.1186/1471-2164-16-S13-S6 (PMC4686781; doi:10.1186/1471-2164-16-S13-S6)
Supplement: Additional file 3 — Primers for qPCR and RT-PCR. [file 1471-2164-16-S13-S6-S3.docx]

**Table** Primers for qPCR and RT-PCR

| Gene | Forward Primer | Reverse Primer | Amplicon size, bp | Efficiency, % |
| --- | --- | --- | --- | --- |
| *Gdf3 (mink)* | GGGGGCTCCCGAGATTTATG | CAAGGGGAGGCTTGAGGAAG | 119 | 97 |
| *Nanog (mink)* | TGCAGAGGAGAGAACTGGGA | TCTGCTGGAGGCTGAGGTAT | 137 | 110 |
| *Nestin (mink)* | GTGAGTCCCGAGAACAGAGG | GAGAAGGGGTGTGACAGGAA | 120 | 108 |
| *Oct4 (mink)* | GATCAGCCACATTGCCCAG | CGTTGCGAATAGTCACTGCT | 159 | 102 |
| *Sox2 (mink)* | CCATCTCCGTGGTCTTCTTT | ATTACCAACGAAGTCAACCT | 131 | 97 |
| *C-MYC (human)*[45] | TGCCTCAAATTGGACTTTGG | GATTGAAATTCTGTGTAACTGC | 192 | - |
| *KLF4 (human)*[45] | GCCACCCACACTTGTGATT | TCCACTCACAAGATGACTCAGT | 408 | - |
| *OCT4 (human)* | CTGCCTCAGCCTCCTGAGTA | AATAGAACCCCCAGGGTGAG | 152 | - |
| *SOX2 (human)* | AGCACCCGGGCCTCAATGCGC | GCACCTCGGCGCCGGGGAGA | 306 | - |
| *Hprt (mink)*[44] | CTTTGCTGACCTGCTGGATT | CACCAATGACTTTGATGTCCC | 134 | 100 |
| *Gapdh (mink)*[44] | AATGCCTCCTGTACCACCAA | GGTCATGAGTCCCTCCACAA | 84 | 104 |
