# Supplementary material for: Comparison of American mink embryonic stem and induced pluripotent stem cell transcriptomes
Source: BMC Genomics. 2015 Dec 16;16(Suppl 13):S6. doi: 10.1186/1471-2164-16-S13-S6 (PMC4686781; doi:10.1186/1471-2164-16-S13-S6)
Supplement: Additional file 4 — qPCR raw data of selected gene expression. [file 1471-2164-16-S13-S6-S4.docx]

**Table** qPCR raw data of selected gene expression

| Sample | *Gapdh* | *Hprt1* | *Oct4m* | *Sox2* | *Gdf3* | *Nes* | *Nanog* |
| --- | --- | --- | --- | --- | --- | --- | --- |
| MES12a | 17,58 | 22,325 | 17,855 | 20,025 | 23,185 | 25,665 | 29,78 |
| MES12b | 17,35 | 22,605 | 18,11 | 19,83 | 23,42 | 25,2 | 30,08 |
| MES12c | 17,685 | 22,125 | 18,335 | 20,05 | 24,6 | 27,24 | 30,69 |
| MES29a | 17,155 | 20,63 | 20,165 | 23,185 | 21,345 | 25,865 | 30,275 |
| MES29b | 17,365 | 20,685 | 20,26 | 23,52 | 21,72 | 25,81 | 30,59 |
| MES29c | 17,16 | 20,645 | 20,34 | 23,33 | 21,12 | 25,385 | 30,11 |
| iNV7a | 17,185 | 20,565 | 18,45 | 22,49 | 22,055 | 27,19 | 30,345 |
| iNV7b | 16,305 | 20,965 | 18,03 | 19,75 | 22,535 | 25,41 | 29,44 |
| iNV7c | 15,77 | 21,09 | 18,11 | 19,91 | 22,865 | 26,565 | 29,41 |
| iNV11a | 17,33 | 21,135 | 18,92 | 22,72 | 22,08 | 26,38 | 30,2 |
| iNV11b | 17,505 | 21,02 | 18,94 | 21,82 | 22,38 | 26,875 | 31,365 |
| iNV11c | 17,375 | 20,74 | 18,97 | 21,99 | 22,185 | 26,55 | 30,535 |
| mink EFa | 16,98 | 23,025 | 30,805 | 25,475 | 33,26 | 28,29 | 36,39 |
| mink EFb | 17,17 | 22,975 | 34,05 | 25,2 | 33,26 | 27,33 | 36,39 |
| mink EFc | 17,43 | 23,105 | 31,01 | 25,27 | 33,26 | 28,675 | 36,39 |

Data has been corrected by PCR efficiency. The values indicate cycles of qPCR where each gene is detected
