# Supplementary material for: Comparison of American mink embryonic stem and induced pluripotent stem cell transcriptomes
Source: BMC Genomics. 2015 Dec 16;16(Suppl 13):S6. doi: 10.1186/1471-2164-16-S13-S6 (PMC4686781; doi:10.1186/1471-2164-16-S13-S6)
Supplement: Additional file 5 — qPCR data of selected gene expression corrected by PCR efficiency and normalized to reference genes Gapdh and Hprt1. [file 1471-2164-16-S13-S6-S5.docx]

**Table** qPCR data of selected gene expression corrected by PCR efficiency and normalized to reference genes *Gapdh* and *Hprt1*

| Sample | *Oct4* | *Sox2* | *Gdf3* | *Nes* | *Nanog* |
| --- | --- | --- | --- | --- | --- |
| MES12a | -2,095 | 0,075 | 3,235 | 5,71 | 9,83 |
| MES12b | -1,865 | -0,15 | 3,44 | 5,22 | 10,105 |
| MES12c | -1,57 | 0,145 | 4,695 | 7,335 | 10,785 |
| MES29a | 1,27 | 4,295 | 2,455 | 6,975 | 11,385 |
| MES29b | 1,23 | 4,5 | 2,7 | 6,79 | 11,57 |
| MES29c | 1,435 | 4,43 | 2,22 | 6,48 | 11,205 |
| iNV7a | -0,42 | 3,615 | 3,175 | 8,31 | 11,475 |
| iNV7b | -0,605 | 1,12 | 3,895 | 6,775 | 10,81 |
| iNV7c | -0,325 | 1,48 | 4,435 | 8,14 | 10,98 |
| iNV11a | -0,315 | 3,49 | 2,845 | 7,15 | 10,97 |
| iNV11b | -0,325 | 2,555 | 3,115 | 7,61 | 12,1 |
| iNV11c | -0,09 | 2,935 | 3,125 | 7,495 | 11,475 |
| mink EFa | 10,8 | 5,47 | 13,255 | 8,285 | 16,39 |
| mink EFb | 13,98 | 5,125 | 13,185 | 7,26 | 16,32 |
| mink EFc | 10,745 | 5 | 12,99 | 8,405 | 16,125 |

Higher values indicate lower expression
