# Supplementary material for: Comparison of American mink embryonic stem and induced pluripotent stem cell transcriptomes
Source: BMC Genomics. 2015 Dec 16;16(Suppl 13):S6. doi: 10.1186/1471-2164-16-S13-S6 (PMC4686781; doi:10.1186/1471-2164-16-S13-S6)
Supplement: Additional file 6 — P values of the pairwise comparison (Tukey-Kramer post-test) of selected gene expression. [file 1471-2164-16-S13-S6-S6.docx]

**Table** *P* values of the pairwise comparison (Tukey-Kramer pots-test) of selected gene expression

| Pairwise comparison | *Oct4* | *Sox2* | *Gdf3* | *Nestin* | *Nanog* |
| --- | --- | --- | --- | --- | --- |
| MES12-MES29 | **0,00685** | **6,89E-05** | 0,0393267 | 0,773733 | 0,0282094 |
| MES12-iNV7 | 0,31813 | 0,0209508 | 0,9999436 | 0,090503 | 0,1238552 |
| MES12-iNV11 | 0,211532 | **0,0016743** | 0,3492767 | 0,211394 | 0,0148798 |
| MSE12-mink EF | **2,00E-08** | **1,57E-05** | **<1E-08** | 0,047238 | **2,00E-08** |
| MES29-iNV7 | 0,152198 | 0,0092448 | 0,0326277 | 0,449774 | 0,8666164 |
| MES29-iNV11 | 0,234211 | 0,1321795 | 0,5921617 | 0,764621 | 0,9927044 |
| MES29m-mink EF | **2,20E-07** | 0,5896978 | **<1E-08** | 0,265164 | **1,80E-07** |
| iNV7-iNV11 | 0,997903 | 0,4566734 | 0,299133 | 0,976162 | 0,6544327 |
| iNV7-mink EF | **5,00E-08** | **0,001129** | **<1E-08** | 0,992372 | **1,00E-07** |
| iNV11-mink EF | **6,00E-08** | 0,0133578 | **<1E-08** | 0,852175 | **2,30E-07** |

In bold – statistically significant *P* values (<0.05) after multiple test-correction (Bonferroni)
