# Supplementary material for: Comparison of American mink embryonic stem and induced pluripotent stem cell transcriptomes
Source: BMC Genomics. 2015 Dec 16;16(Suppl 13):S6. doi: 10.1186/1471-2164-16-S13-S6 (PMC4686781; doi:10.1186/1471-2164-16-S13-S6)
Supplement: Additional file 7 — Number of pair-end reads and base in mink cell lines. [file 1471-2164-16-S13-S6-S7.docx]

**Table** Number of pair-end reads and base in mink cell lines

| Cell line, replica | Number of pair-end reads | Number of base pairs |
| --- | --- | --- |
| iNV7, a | 13,687,123 | 1,396,086,546 |
| iNV7, b | 23,885,694 | 2,436,340,788 |
| iNV7, c | 12,639,871 | 1,289,266,842 |
| iNV11, a | 7,937,544 | 809,629,488 |
| iNV11, b | 9,710,087 | 990,428,874 |
| iNV11, c | 11,298,165 | 1,152,412,830 |
| MES12, a | 8,992,607 | 917,245,914 |
| MES12, b | 5,925,115 | 604,361,730 |
| MES12, c | 13,228,226 | 1,349,279,052 |
| MES29, a | 17,325,927 | 1,767,244,554 |
| MES29, b | 4,055,964 | 413,708,328 |
| MES29, c | 13,205,251 | 1,346,935,602 |
| Mink EF, a | 10,890,730 | 1,110,854,460 |
| Mink EF, b | 10,170,474 | 1,037,388,348 |
| Mink EF, c | 9,406,309 | 959,443,518 |
