# Supplementary material for: Comparison of American mink embryonic stem and induced pluripotent stem cell transcriptomes
Source: BMC Genomics. 2015 Dec 16;16(Suppl 13):S6. doi: 10.1186/1471-2164-16-S13-S6 (PMC4686781; doi:10.1186/1471-2164-16-S13-S6)
Supplement: Additional file 9 — Expression levels of selected pluripotency-associated genes in mink EF, ES and iPS cells. Vertical axis represents counts determined for each sample by transcriptome analysis. [file 1471-2164-16-S13-S6-S9.pdf]

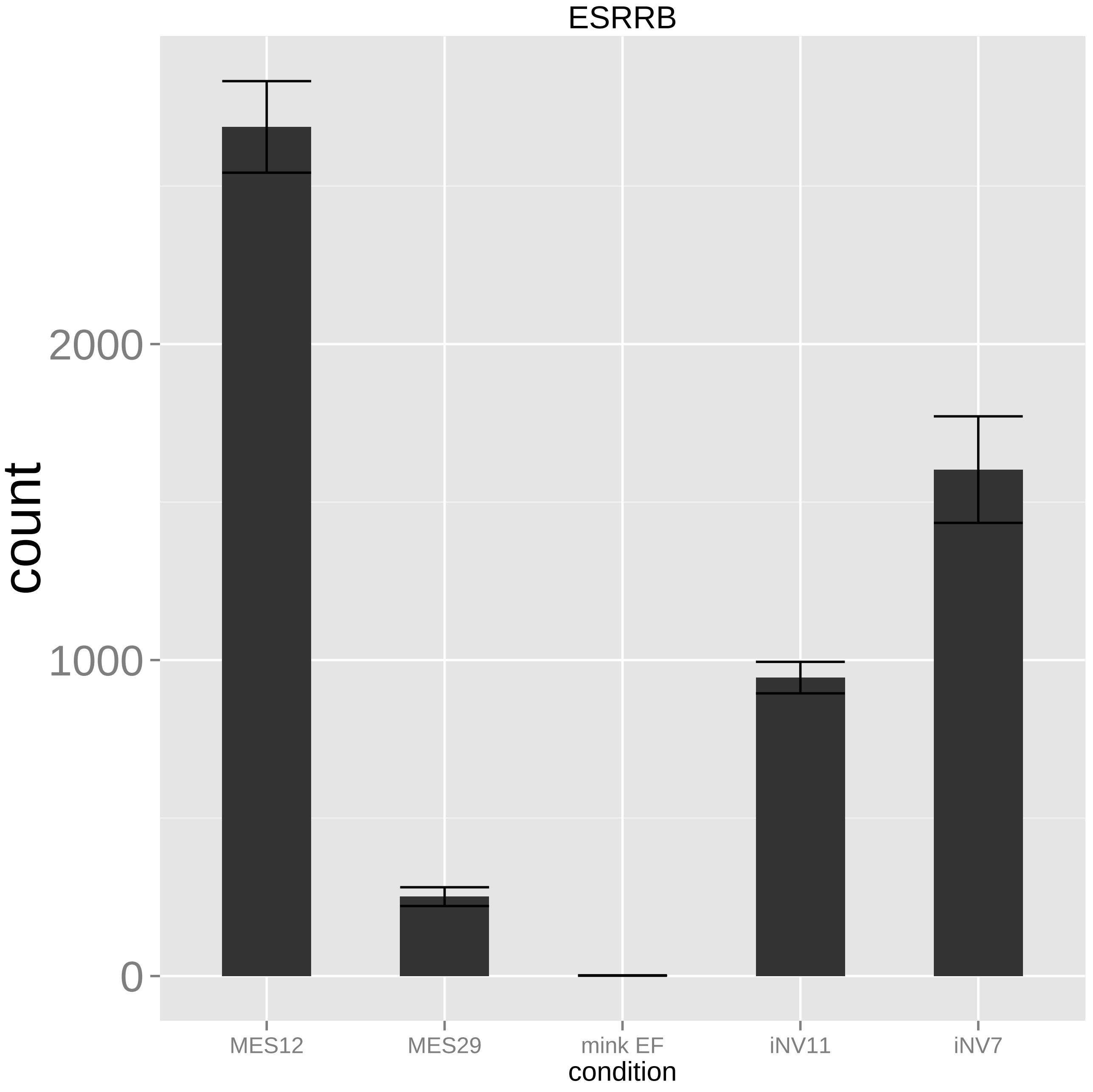
ESRRB
count
2000
1000
0
MES12
MES29
mink EF
iNV11
iNV7
condition

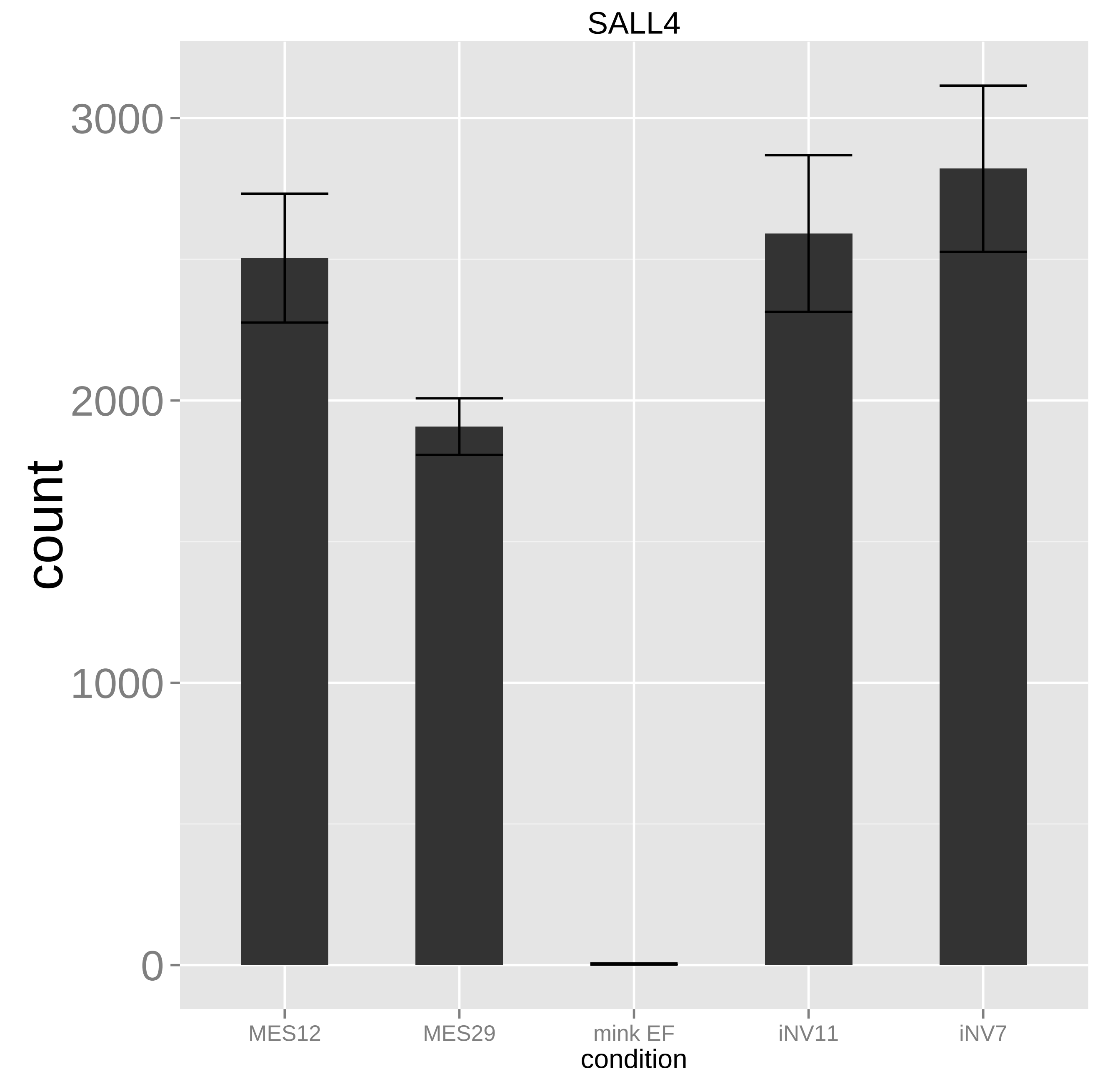
SALL4
count
3000
2000
1000
0
MES12
MES29
mink EF
iNV11
iNV7
condition

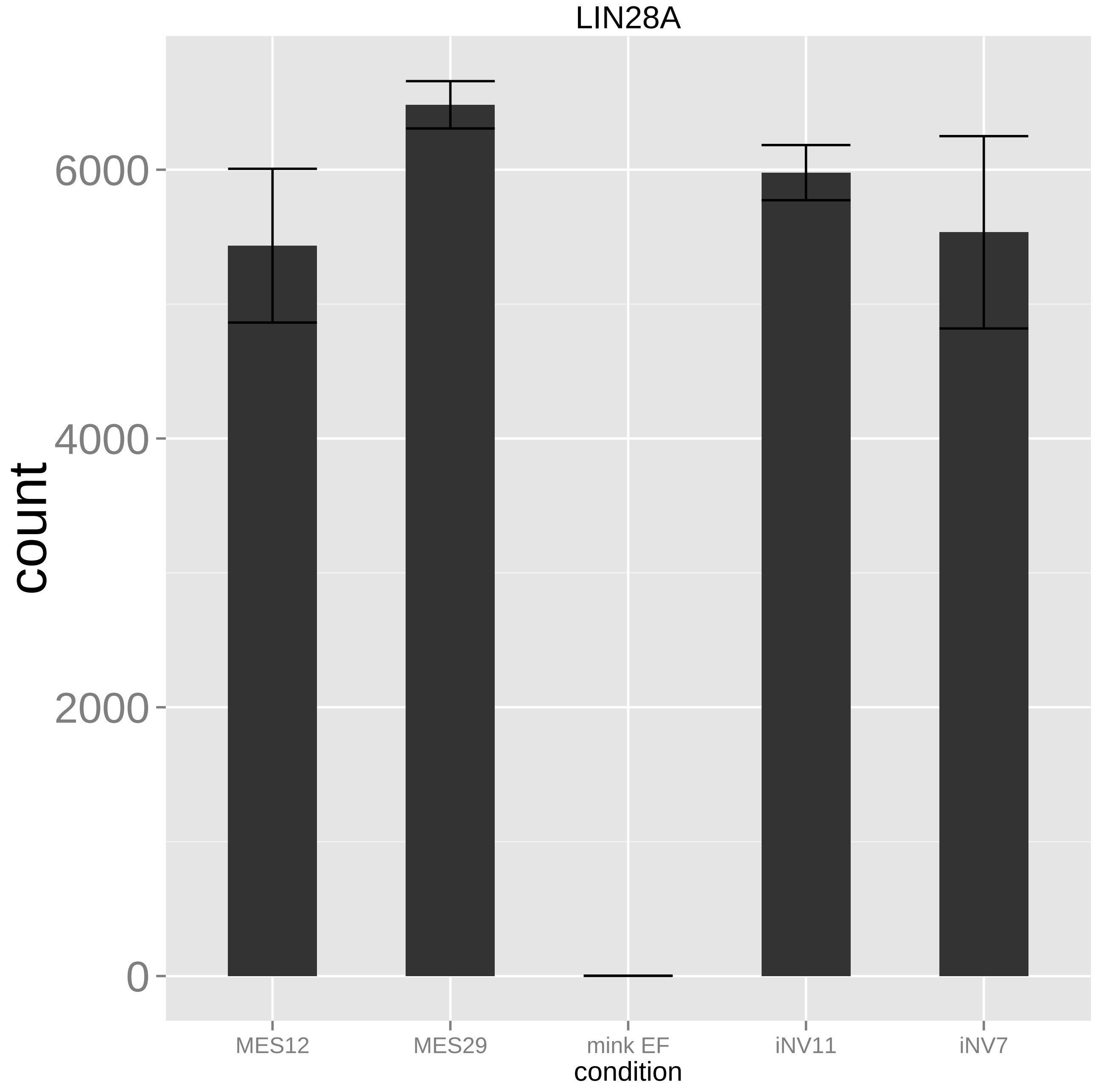

LIN28A
count
6000
4000
2000
0
MES12
MES29
mink EF
iNV11
iNV7
condition

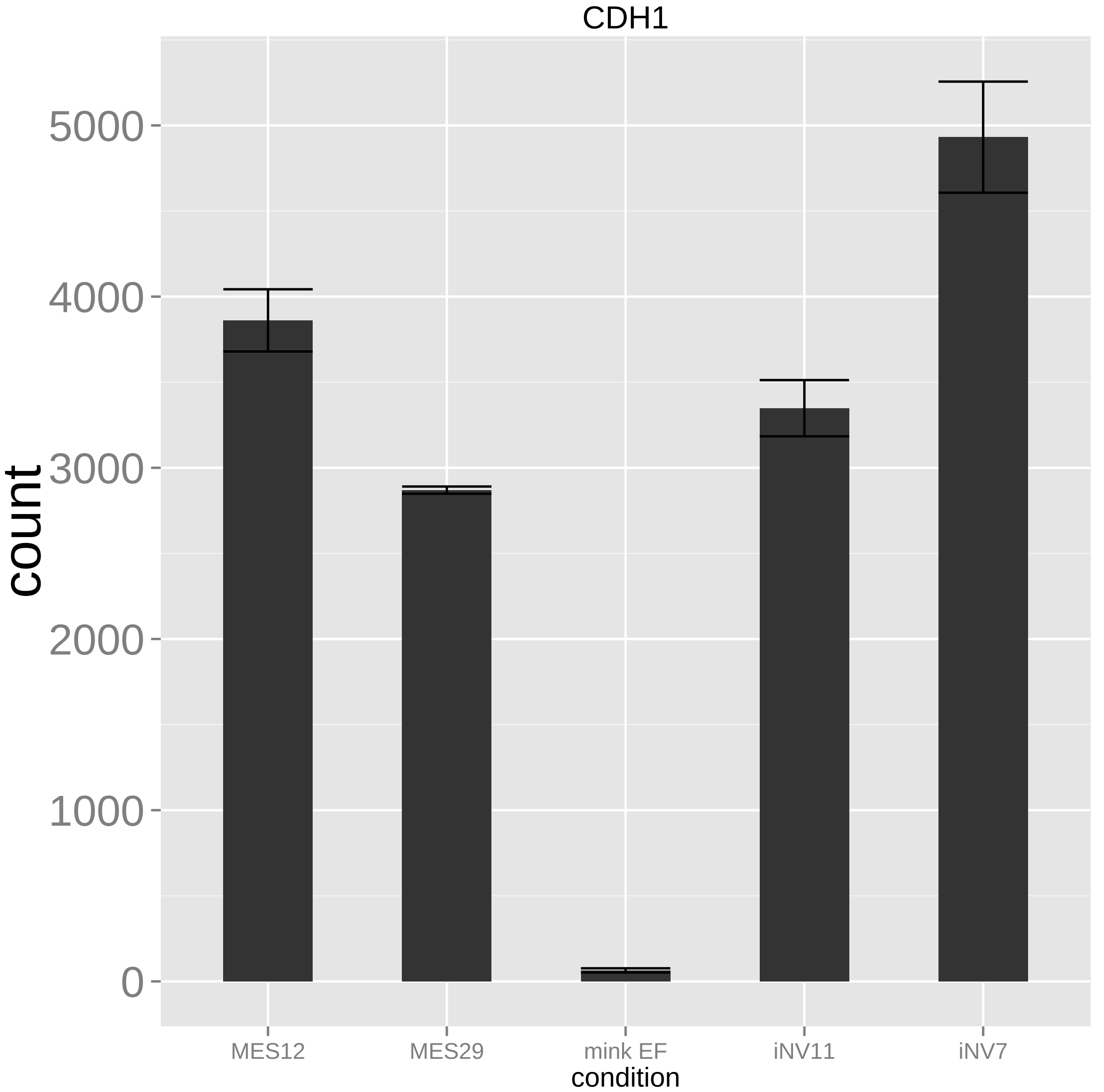

CDH1
5000
4000
3000
2000
1000
0
count
MES12
MES29
mink EF
iNV11
iNV7
condition

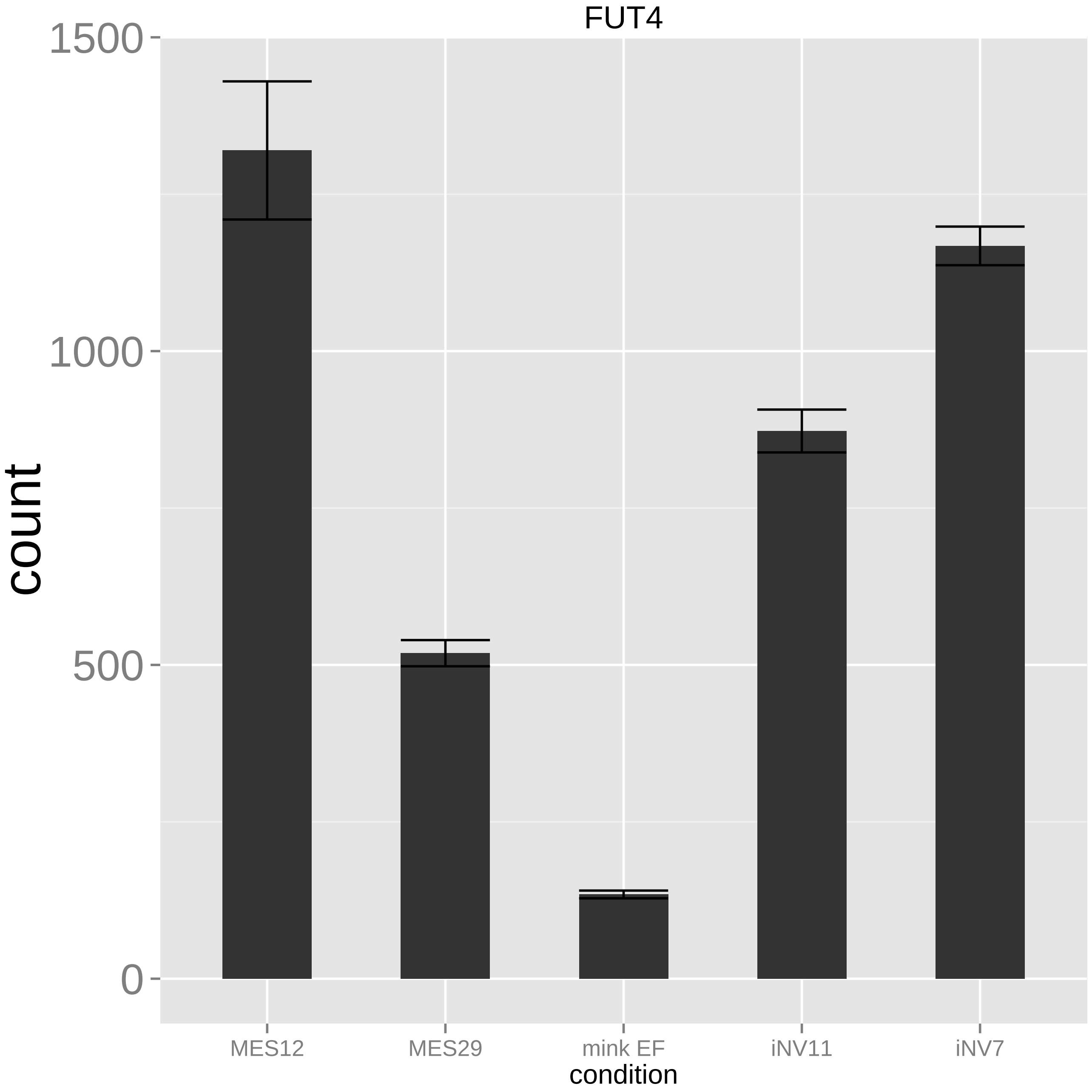
FUT4
1500
1000
500
0
count
MES12
MES29
mink EF
iNV11
iNV7
condition

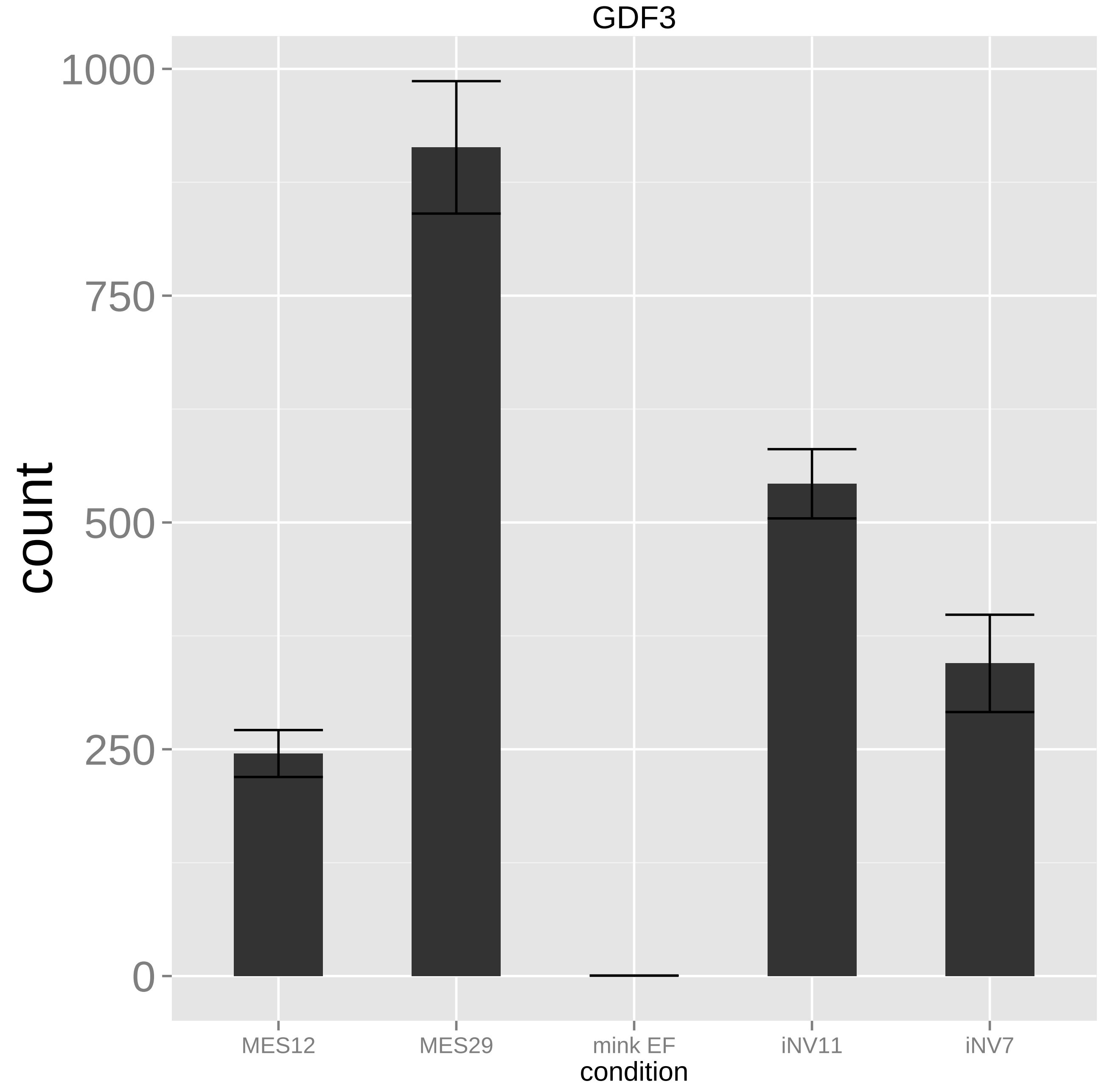
GDF3
count
1000
750
500
250
0
MES12
MES29
mink EF
iNV11
iNV7
condition

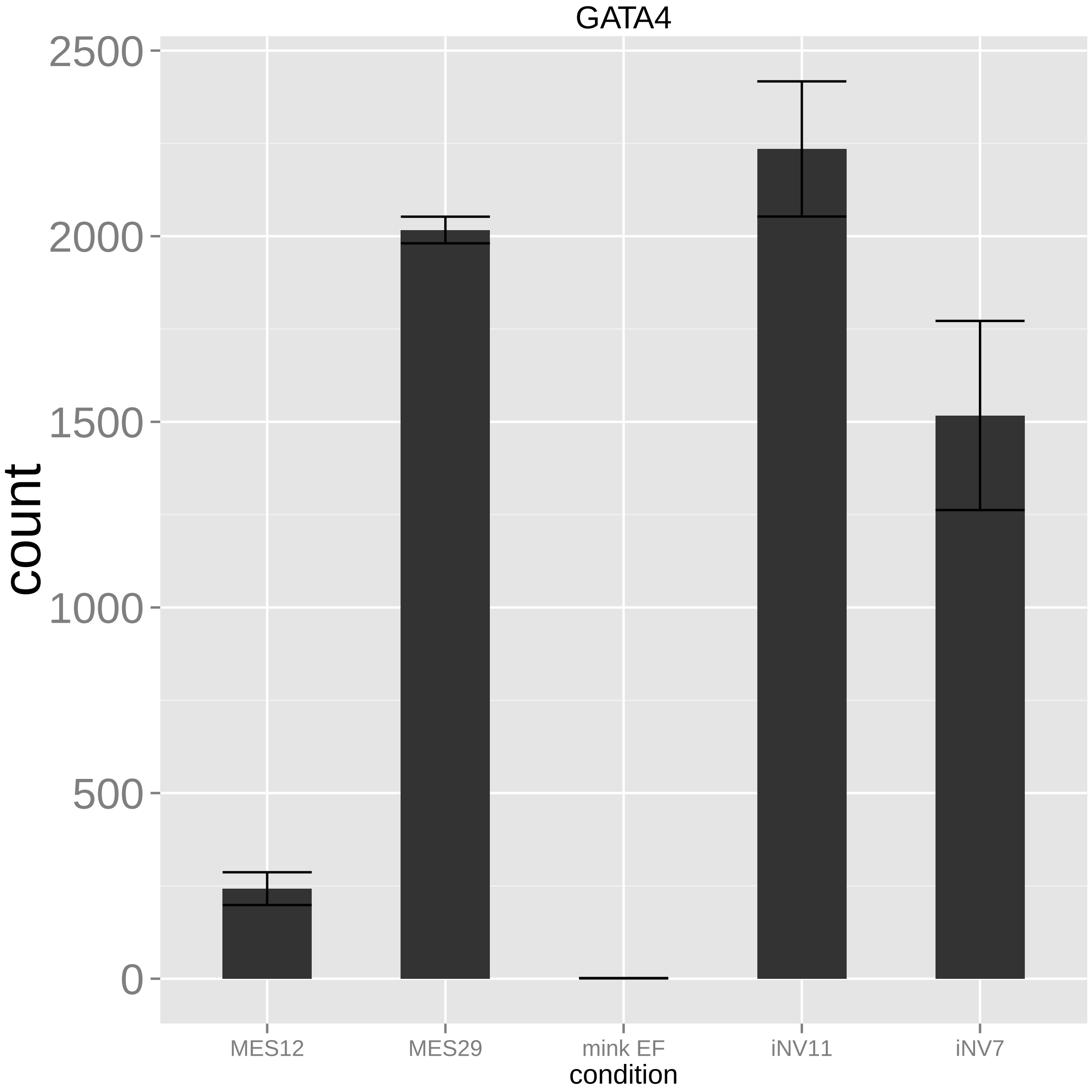
GATA4
count
2500
2000
1500
1000
500
0
MES12
MES29
mink EF
iNV11
iNV7
condition

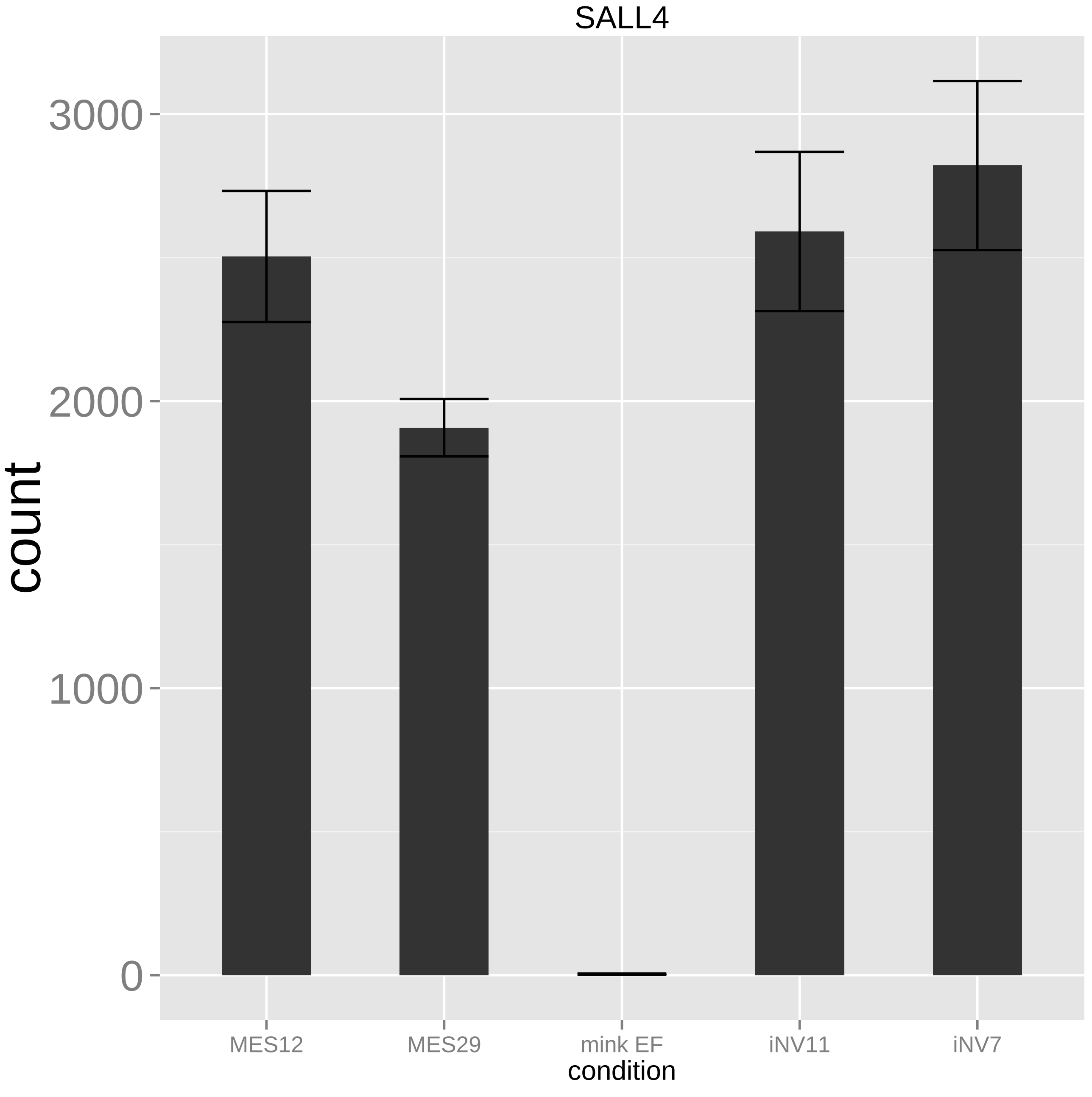
SALL4
count
3000
2000
1000
0
MES12
MES29
mink EF
iNV11
iNV7
condition

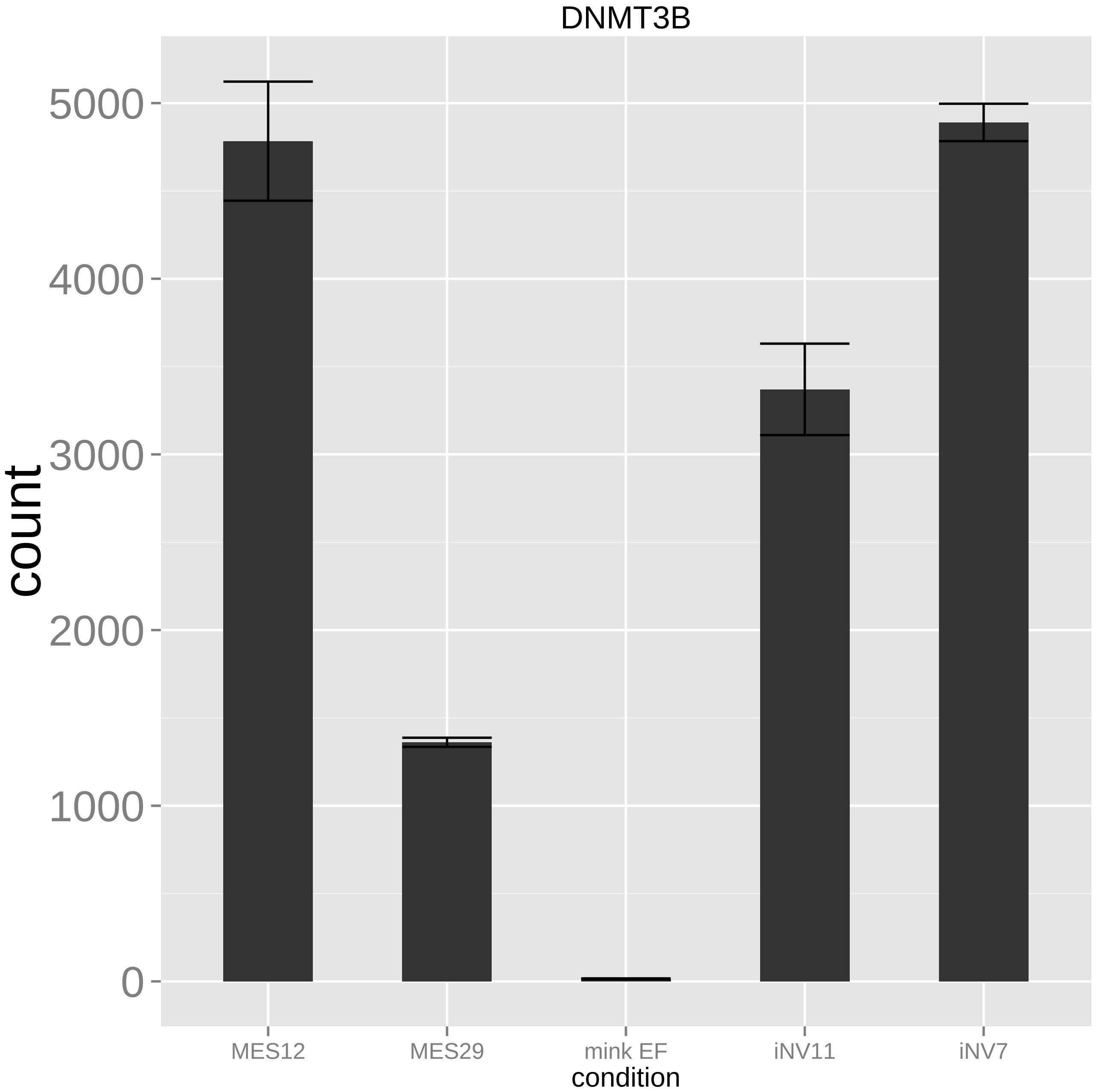

DNMT3B
count
5000
4000
3000
2000
1000
0
MES12
MES29
mink EF
iNV11
iNV7
condition

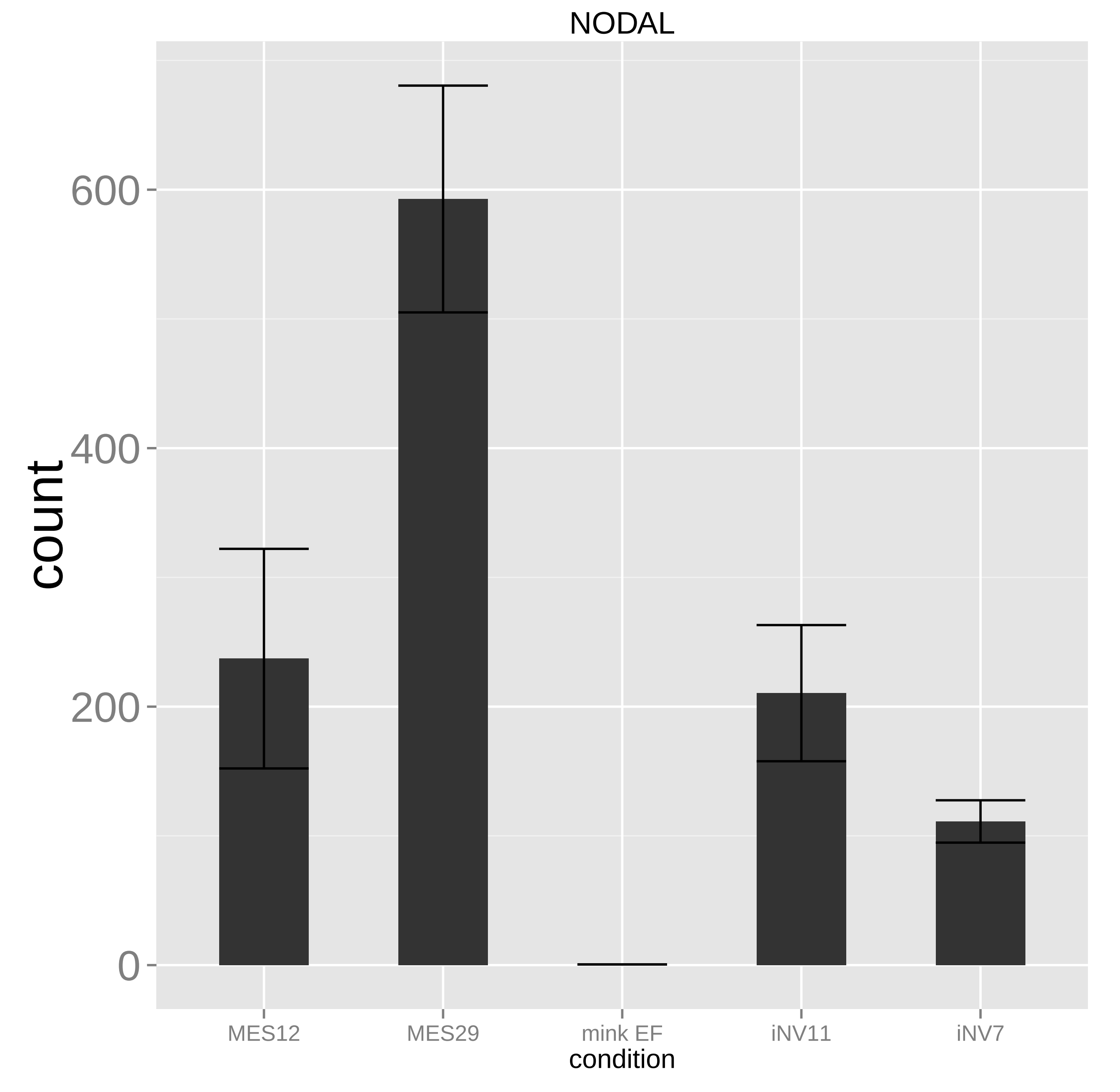
NODAL
count
600
400
200
0
MES12
MES29
mink EF
iNV11
iNV7
condition

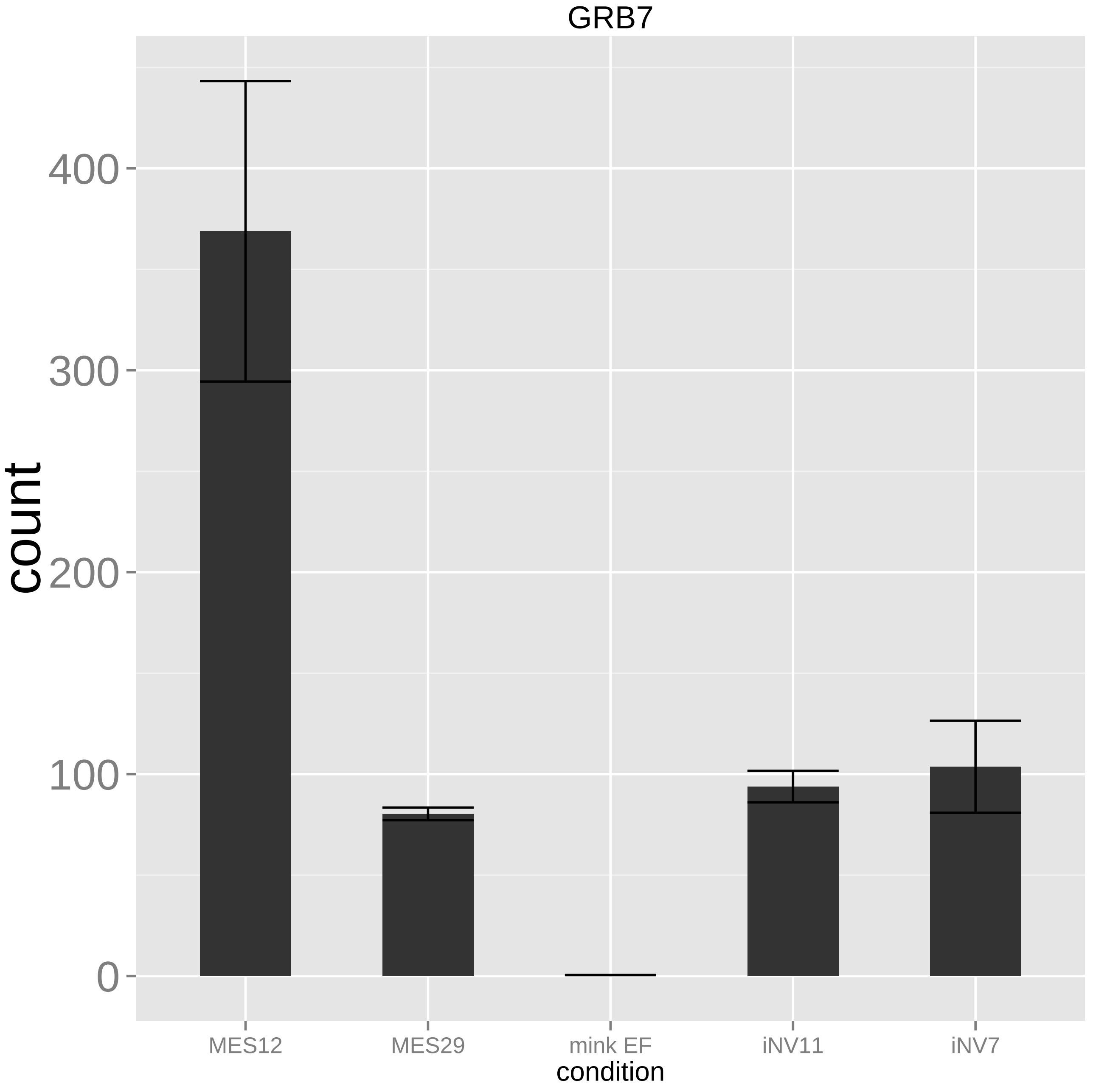
GRB7
count
400
300
200
100
0
MES12
MES29
mink EF
iNV11
iNV7
condition
