# Supplementary material for: Comparison of American mink embryonic stem and induced pluripotent stem cell transcriptomes
Source: BMC Genomics. 2015 Dec 16;16(Suppl 13):S6. doi: 10.1186/1471-2164-16-S13-S6 (PMC4686781; doi:10.1186/1471-2164-16-S13-S6)
Supplement: Additional file 10 — Expression levels of shared between iNV7 and iNV11 genes with "novel" expression pattern in EF, ES and iPS cells. Vertical axis represents counts determined for each sample by transcriptome analysis. [file 1471-2164-16-S13-S6-S10.pdf]

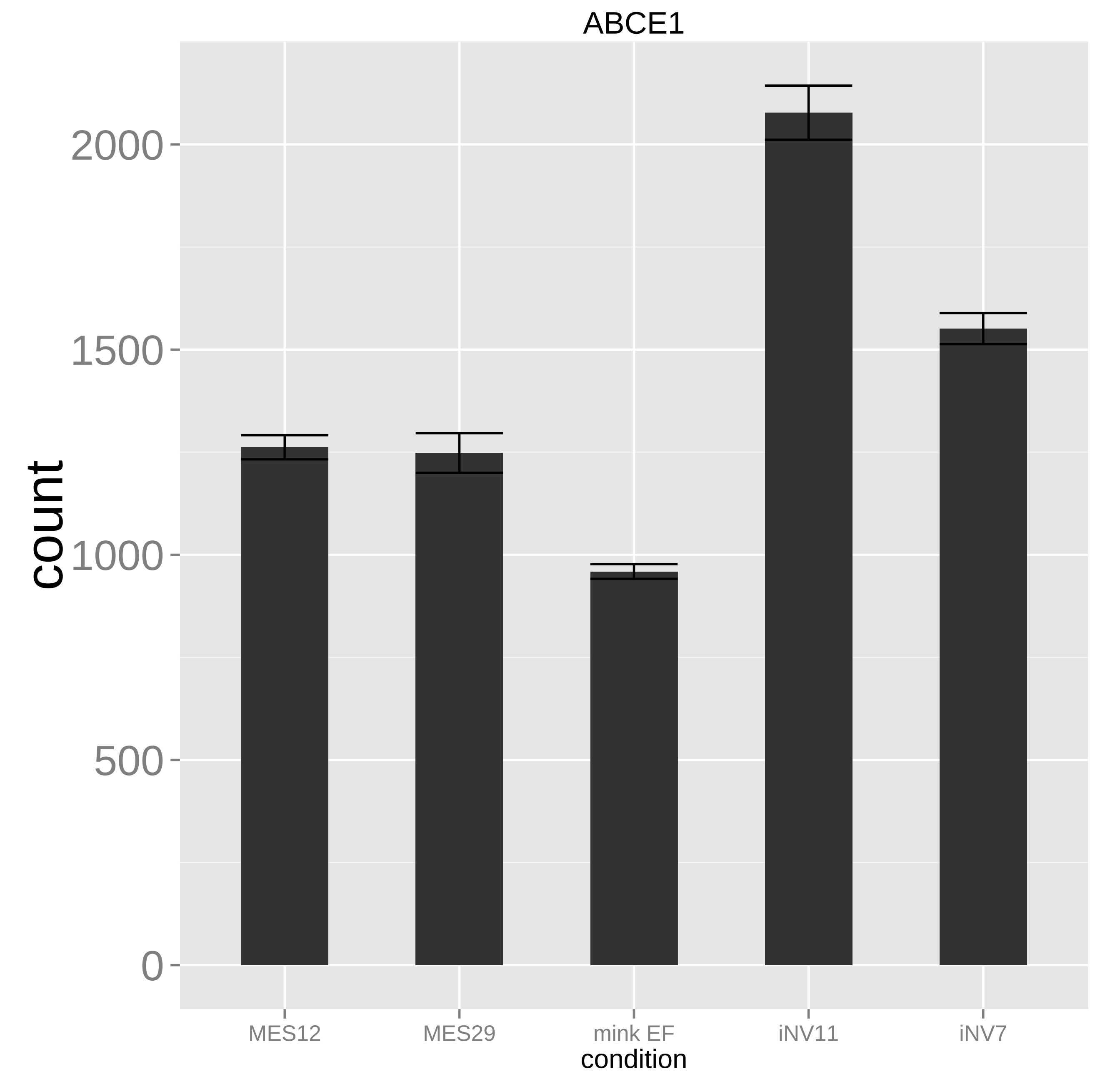
ABCE1
count
2000
1500
1000
500
0
MES12
MES29
mink EF
iNV11
iNV7
condition

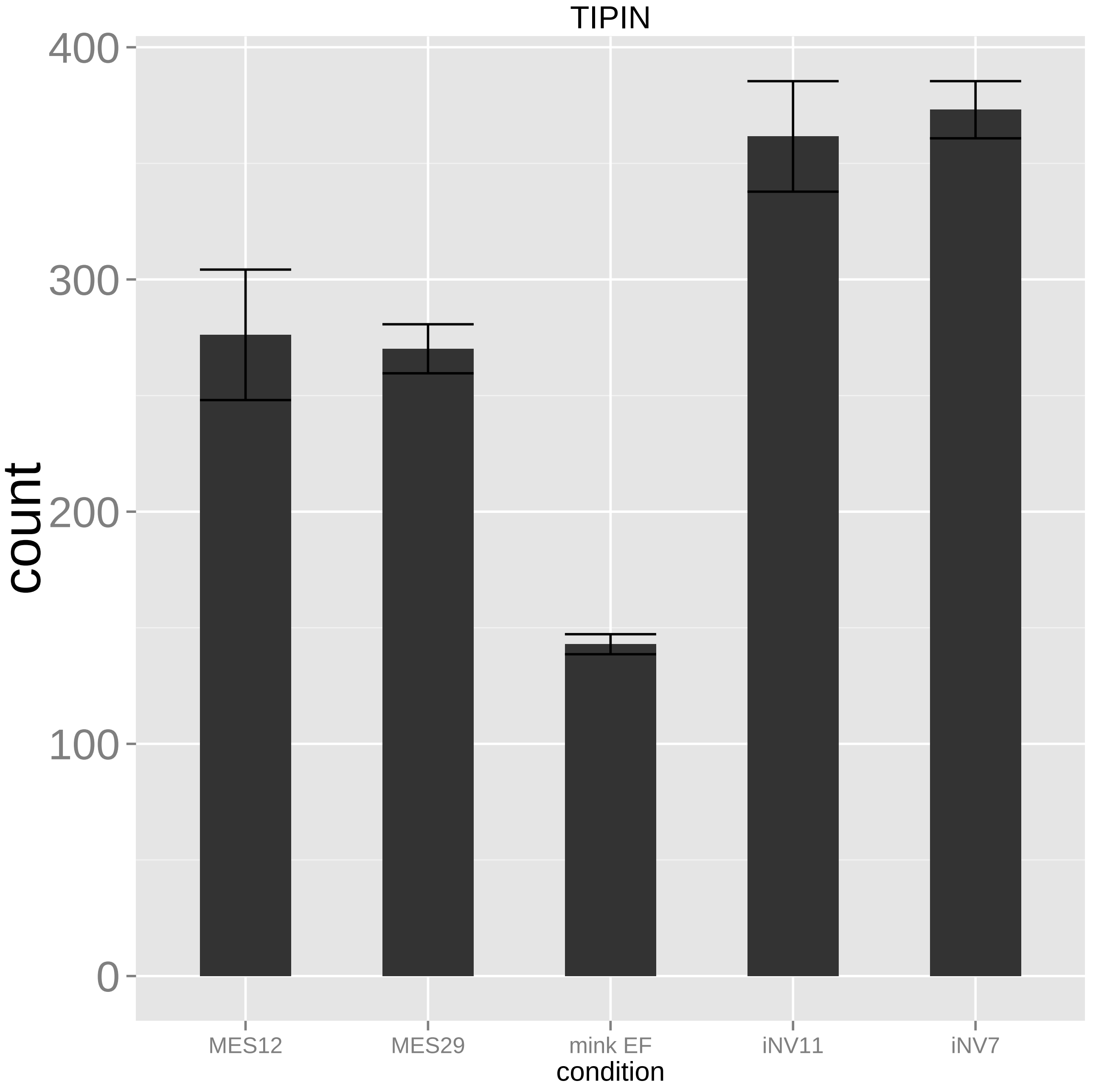

TIPIN
400
300
200
100
0
count
MES12
MES29
mink EF
iNV11
iNV7
condition

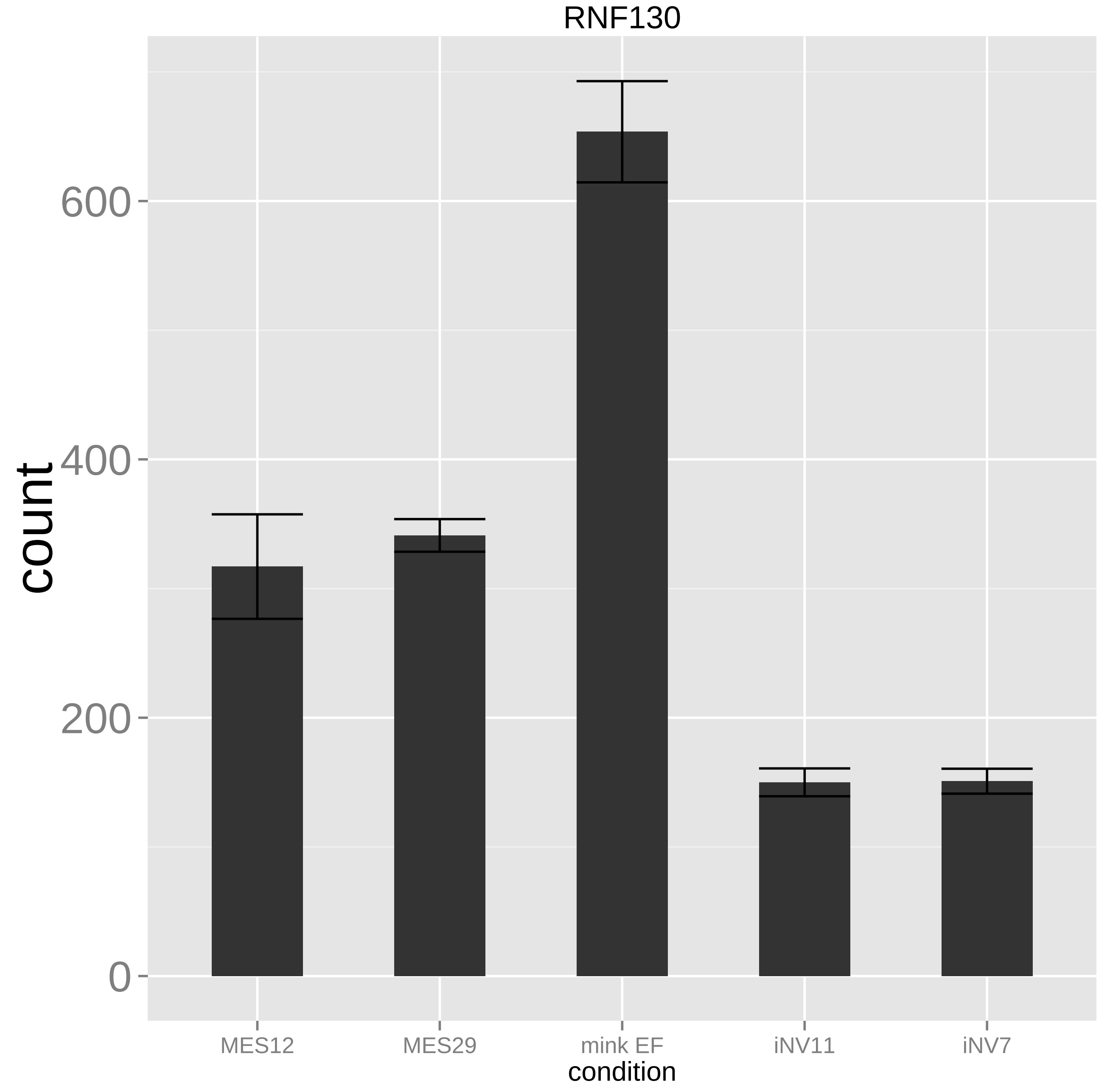

RNF130
count
600
400
200
0
MES12
MES29
mink EF
iNV11
iNV7
condition

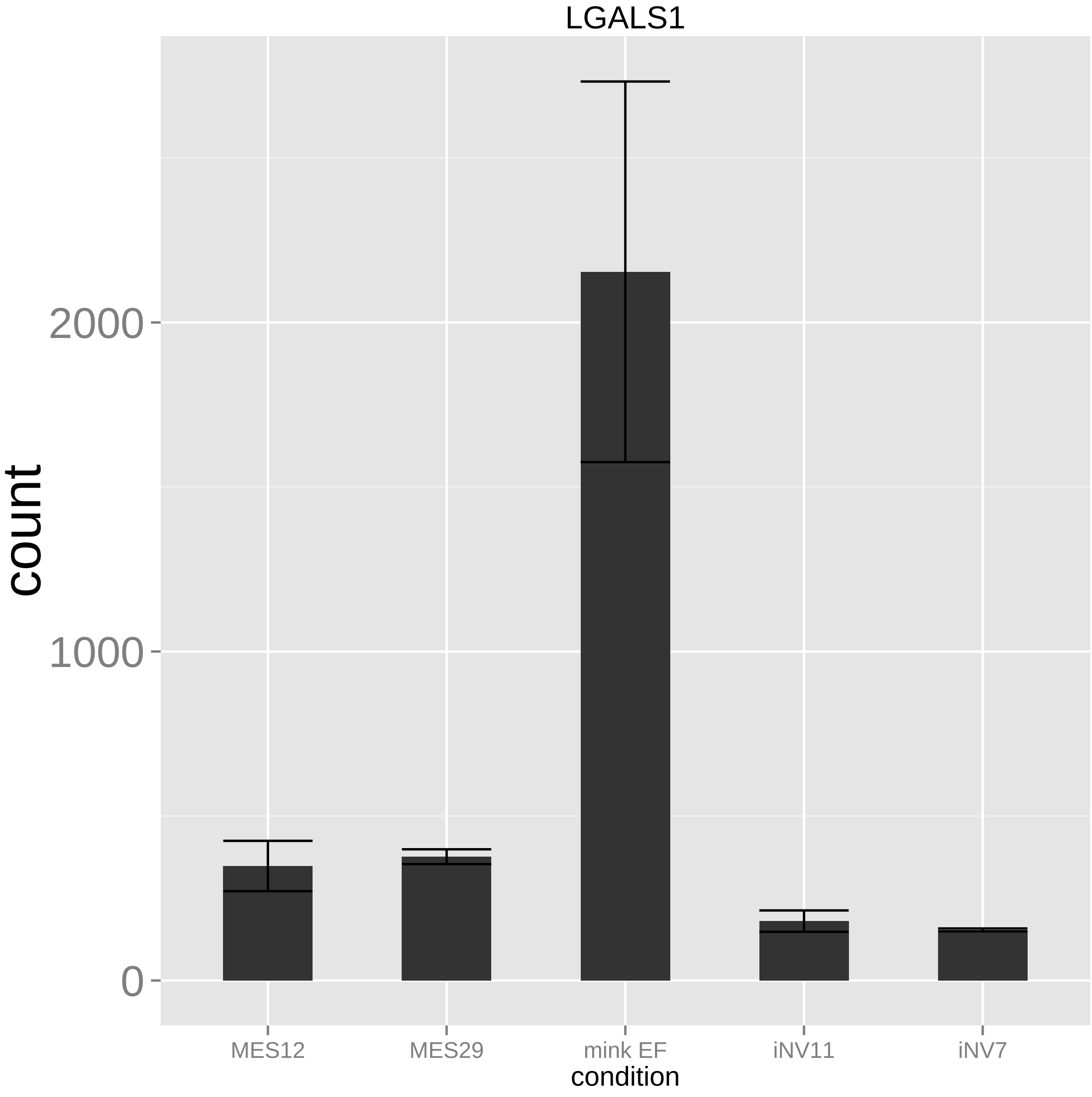
LGALS1
count
2000
1000
0
MES12
MES29
mink EF
iNV11
iNV7
condition

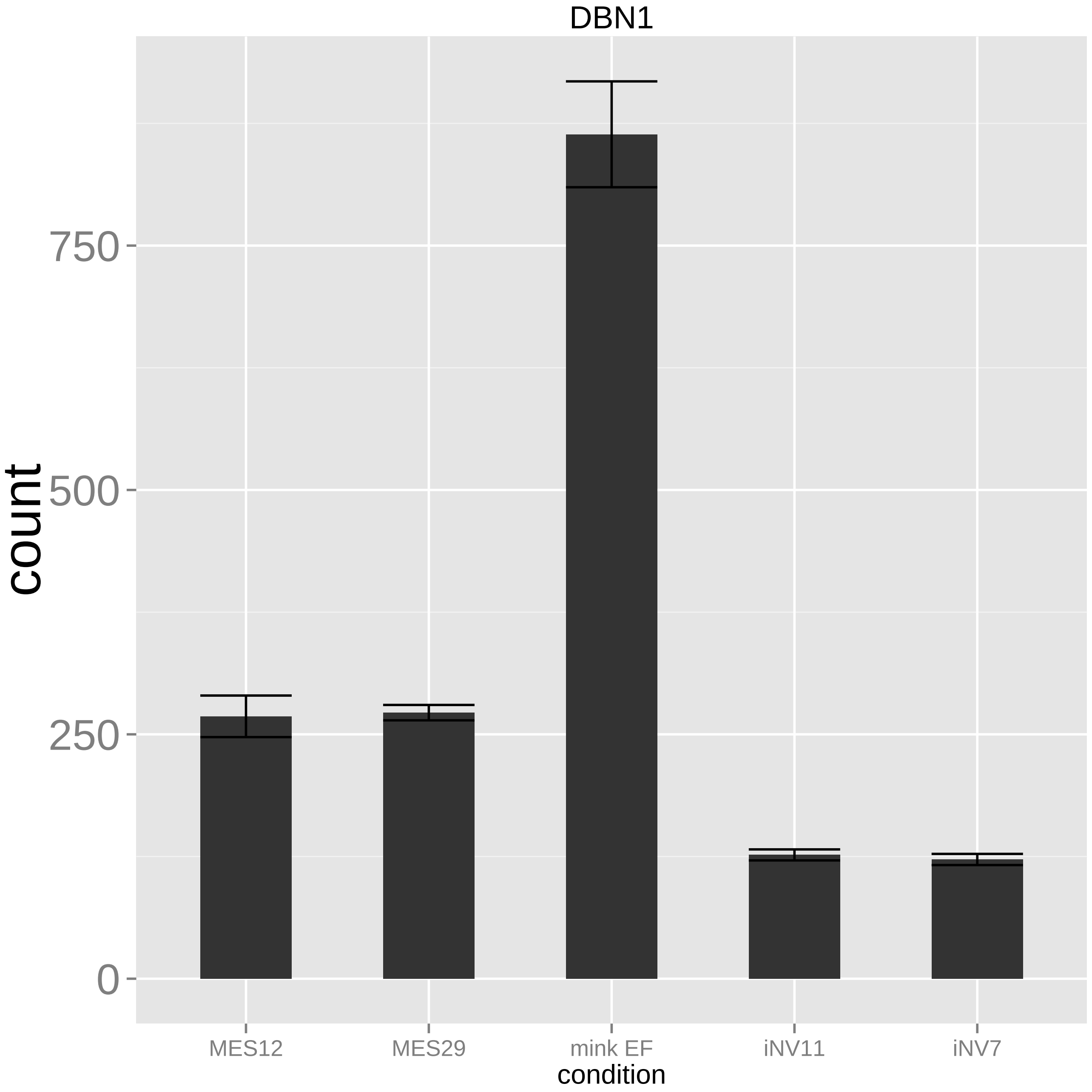
DBN1
count
750
500
250
0
MES12
MES29
mink EF
iNV11
iNV7
condition

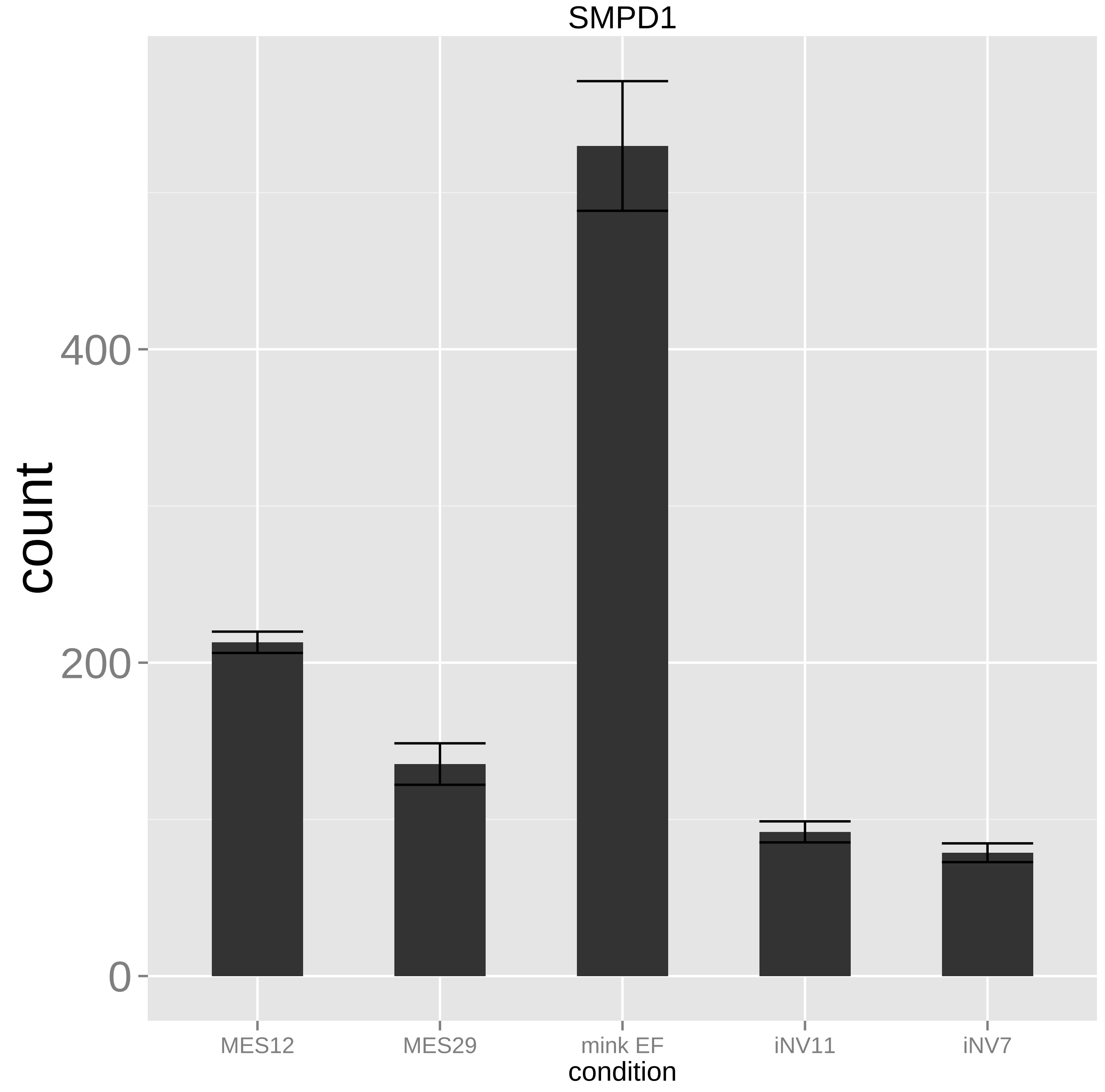
SMPD1
count
400
200
0
MES12
MES29
mink EF
iNV11
iNV7
condition

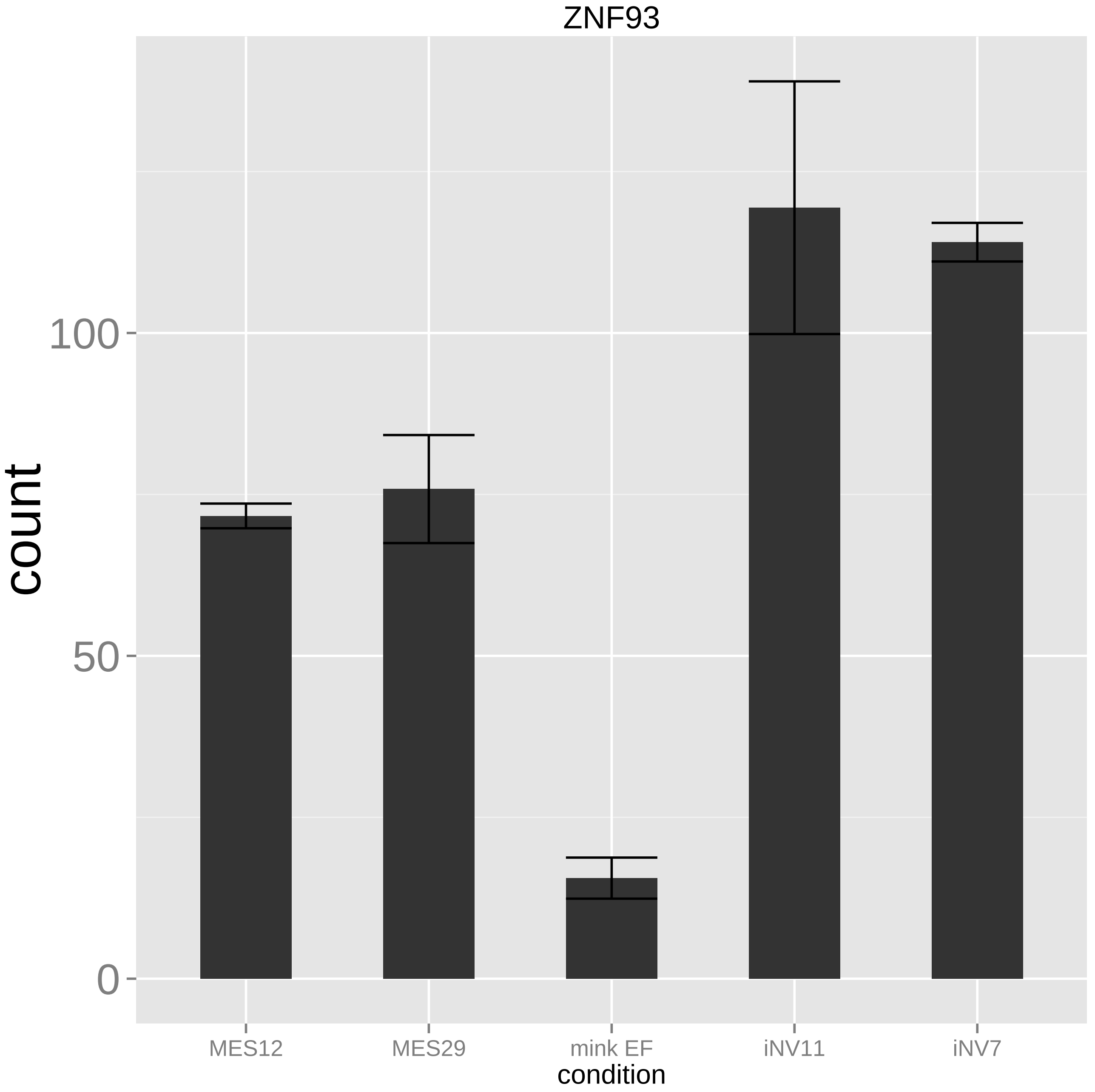
ZNF93
count
100
50
0
MES12
MES29
mink EF
iNV11
iNV7
condition

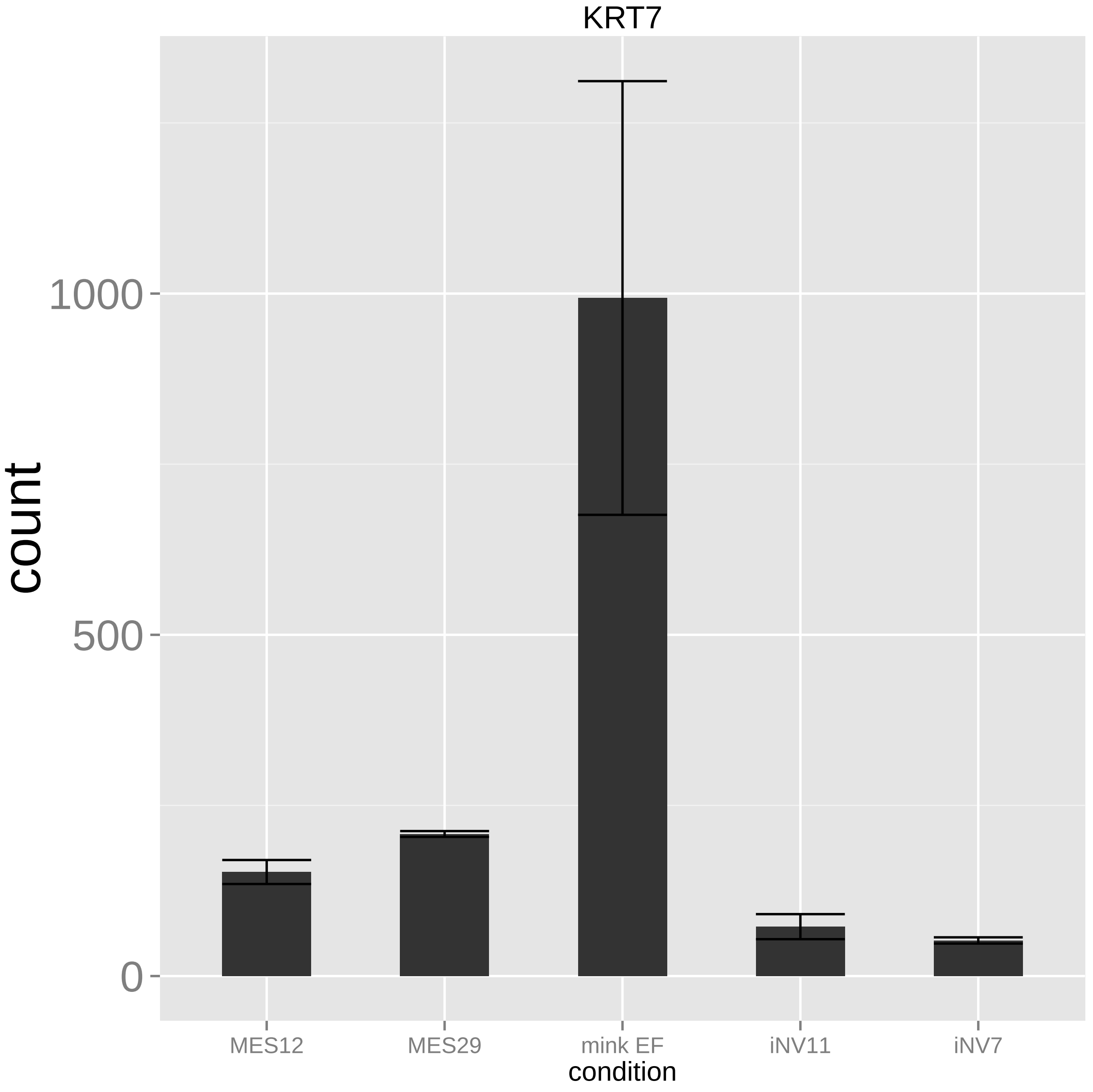
KRT7
count
1000
500
0
MES12
MES29
mink EF
iNV11
iNV7
condition

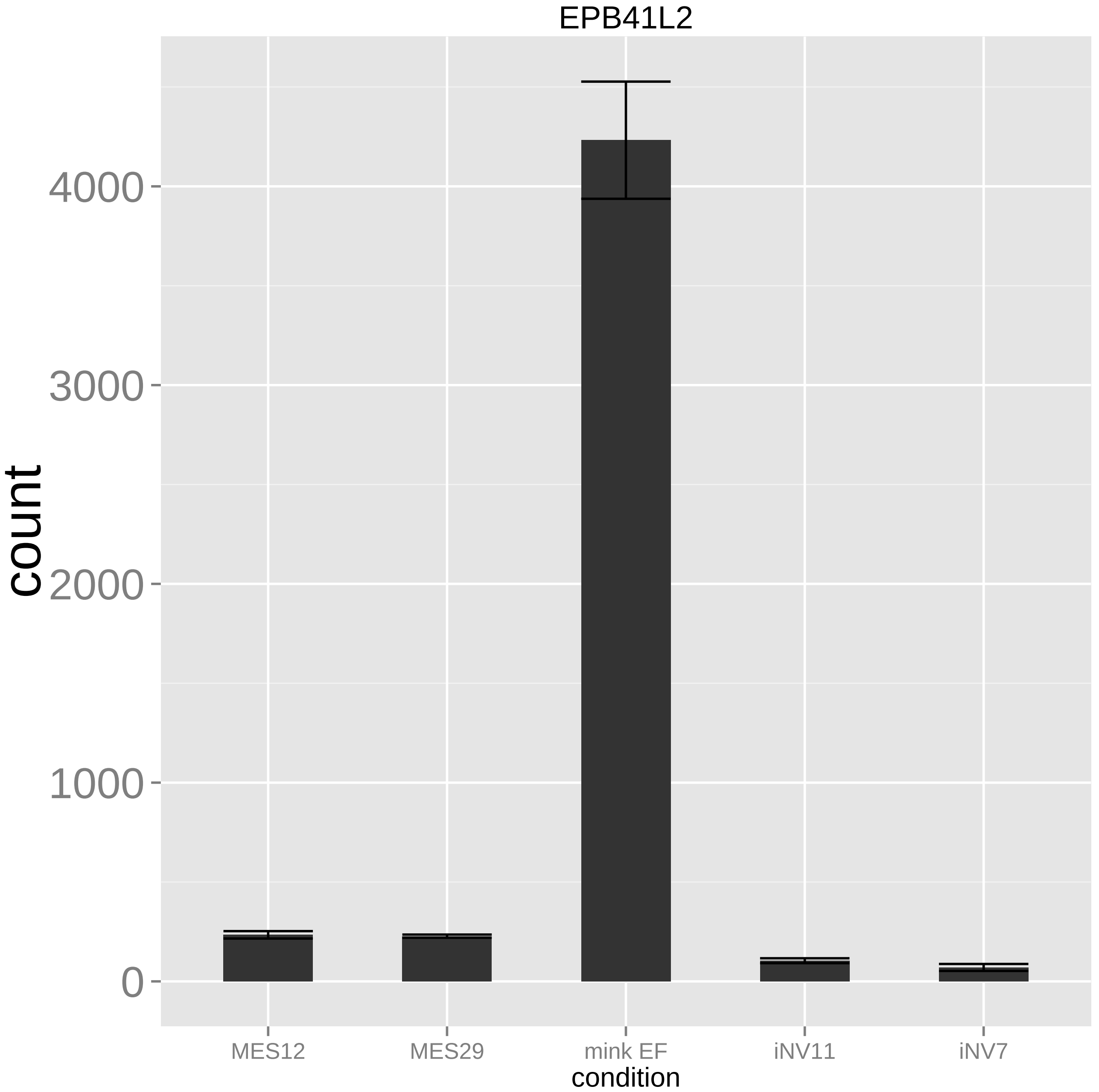
EPB41L2
count
4000
3000
2000
1000
0
MES12
MES29
mink EF
iNV11
iNV7
condition

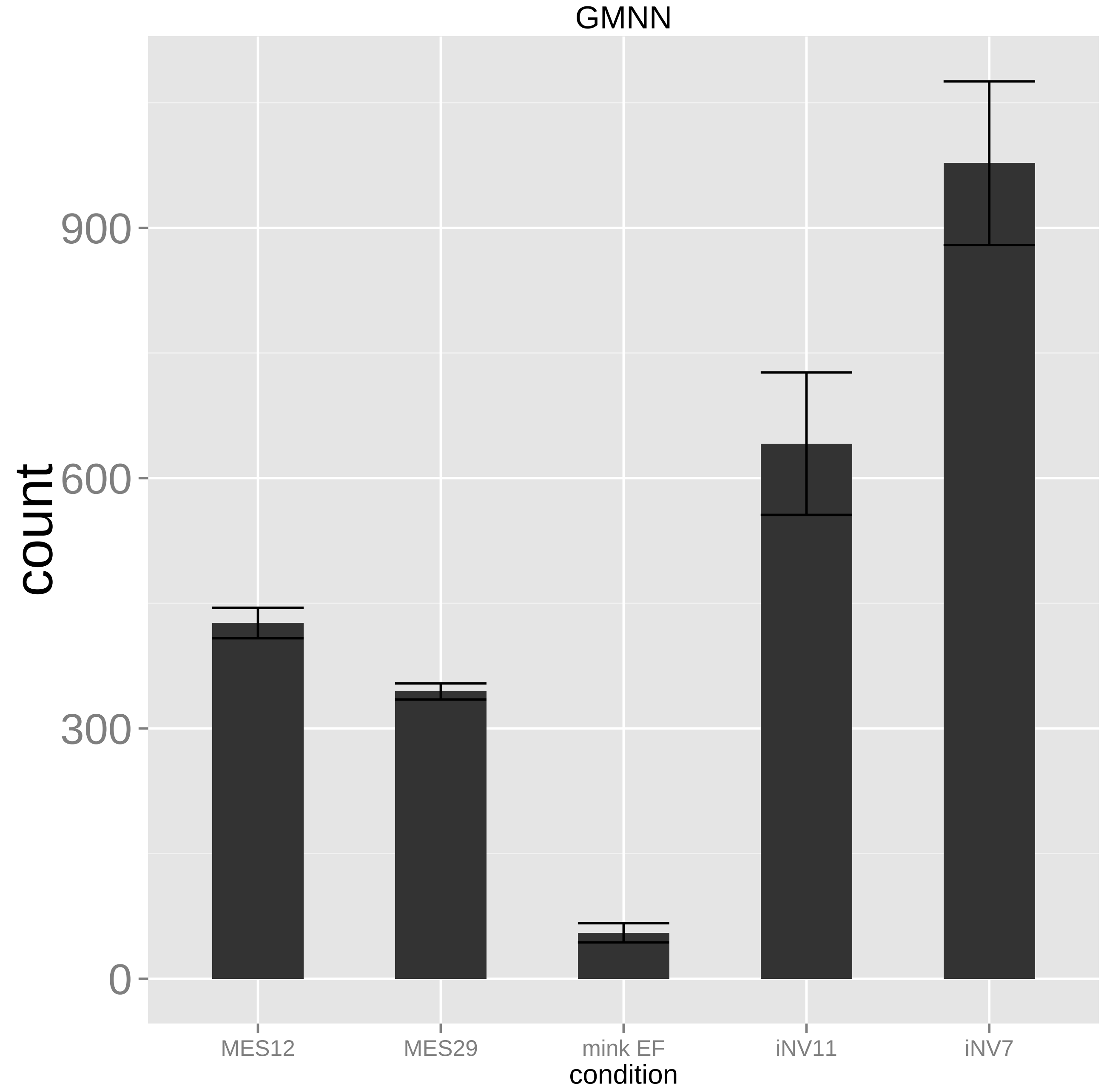
GMNN
count
900
600
300
0
MES12
MES29
mink EF
iNV11
iNV7
condition

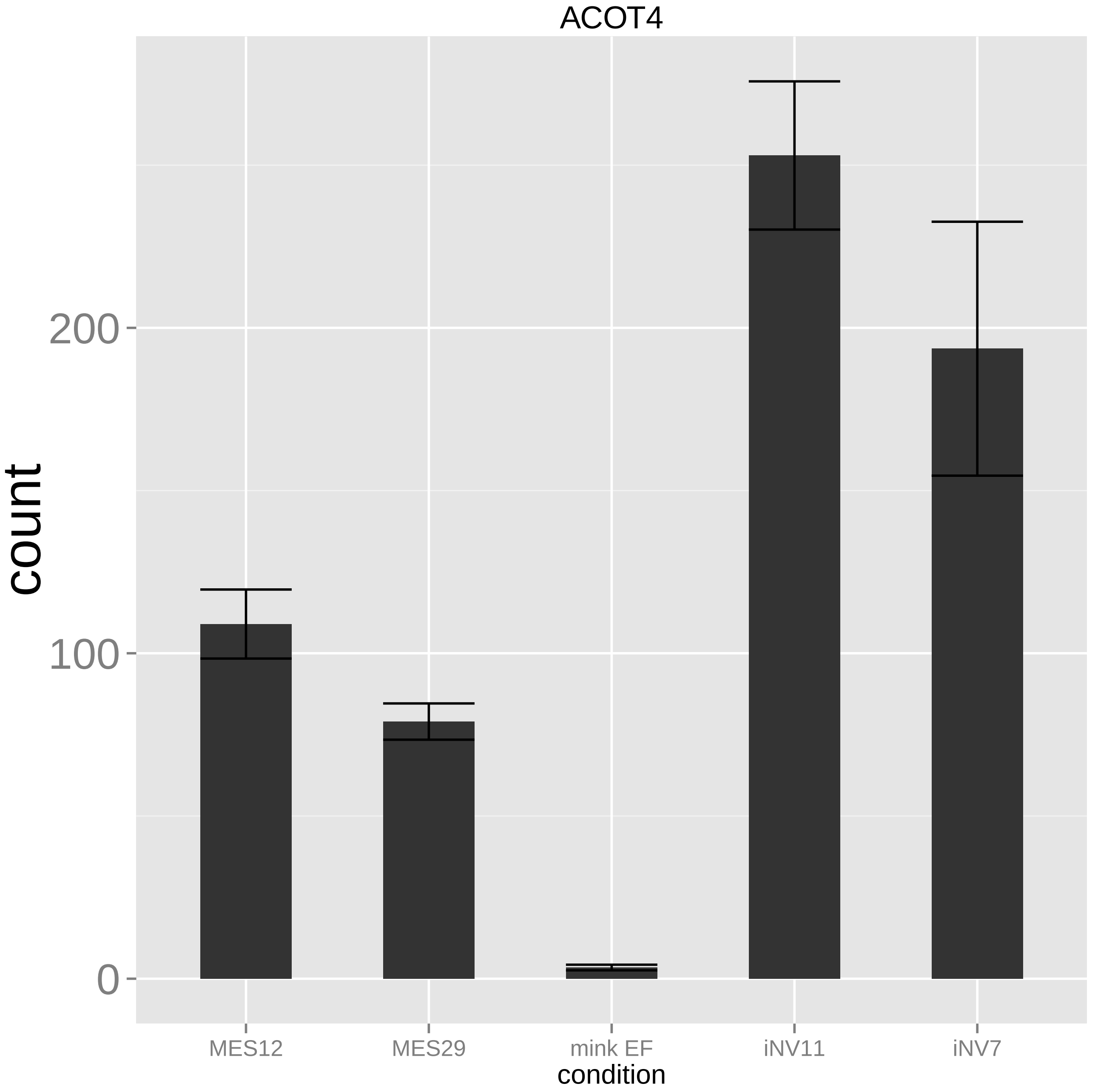
ACOT4
count
200
100
0
MES12
MES29
mink EF
iNV11
iNV7
condition

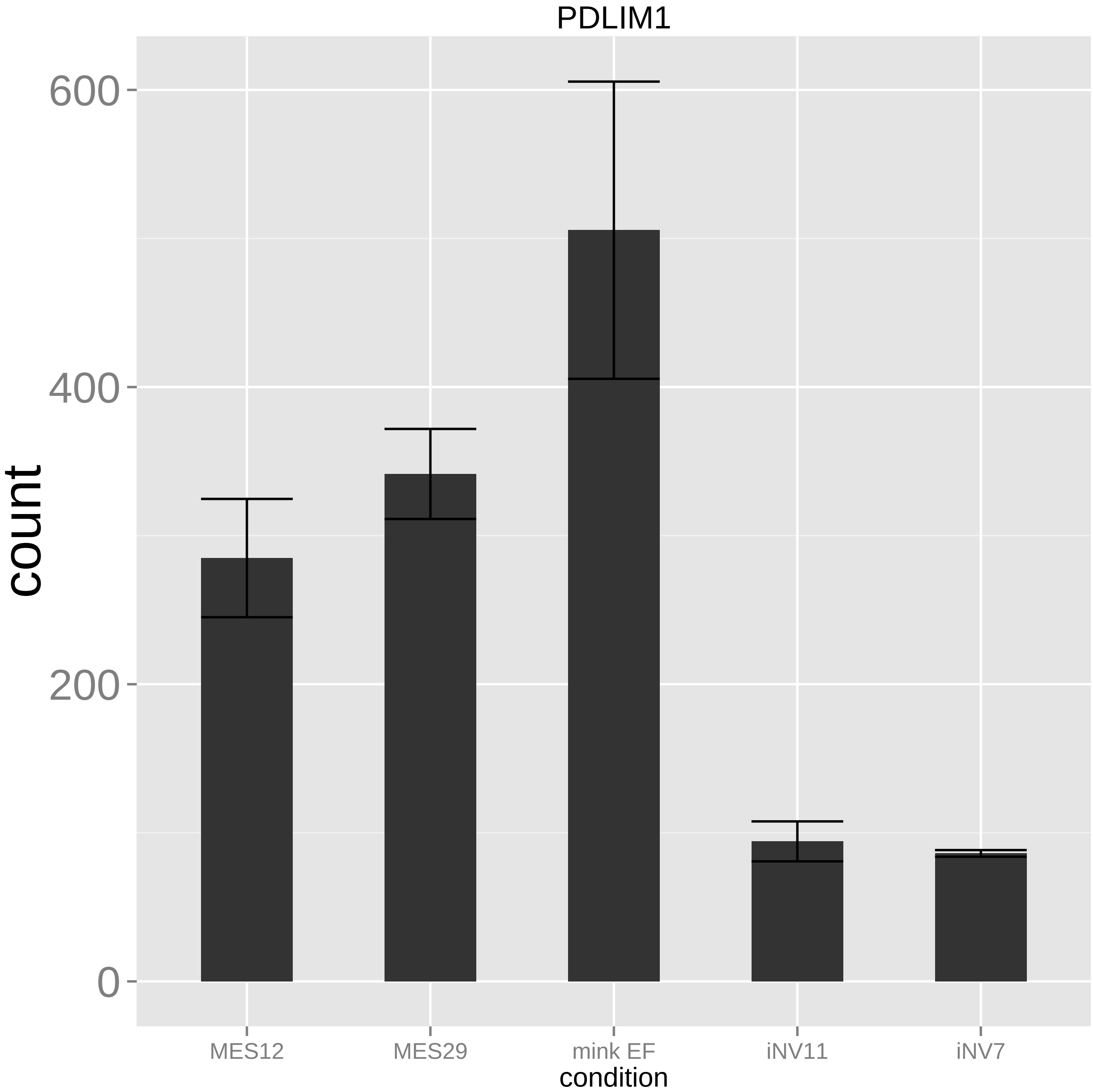
PDLIM1
600
400
200
0
count
MES12
MES29
mink EF
iNV11
iNV7
condition

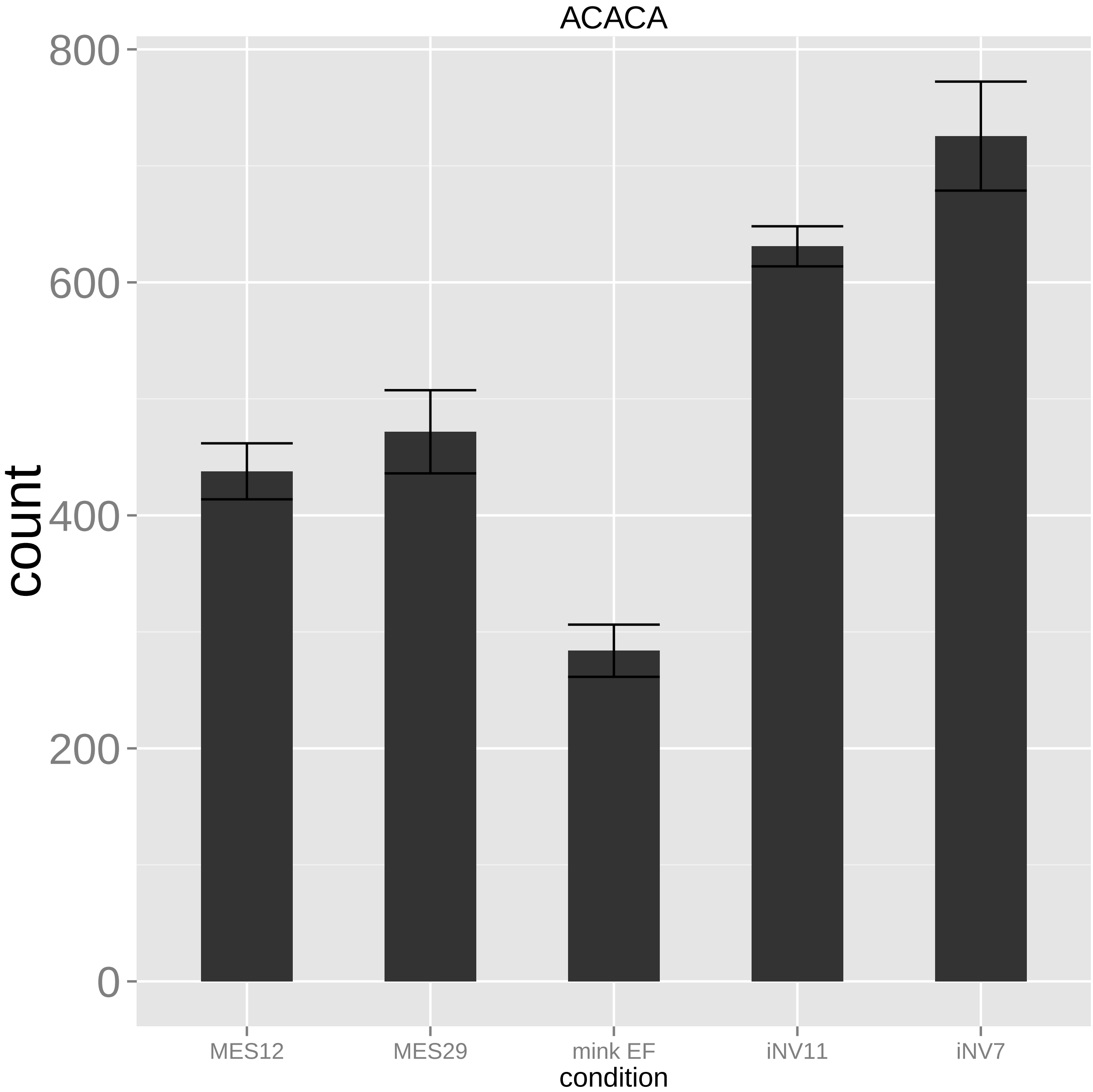
ACACA
count
800
600
400
200
0
MES12
MES29
mink EF
iNV11
iNV7
condition

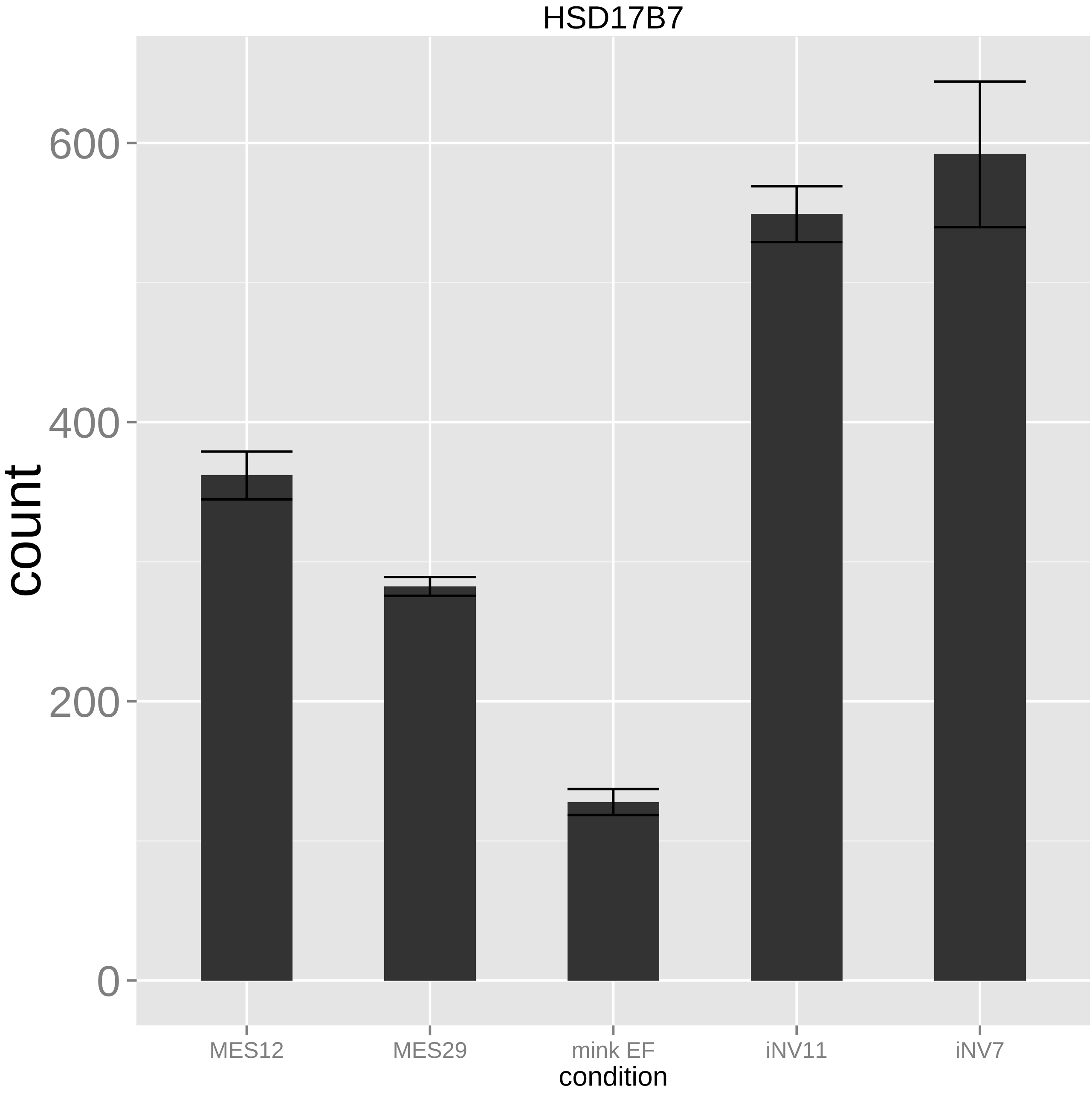

HSD17B7
600
400
200
0
count
MES12
MES29
mink EF
iNV11
iNV7
condition

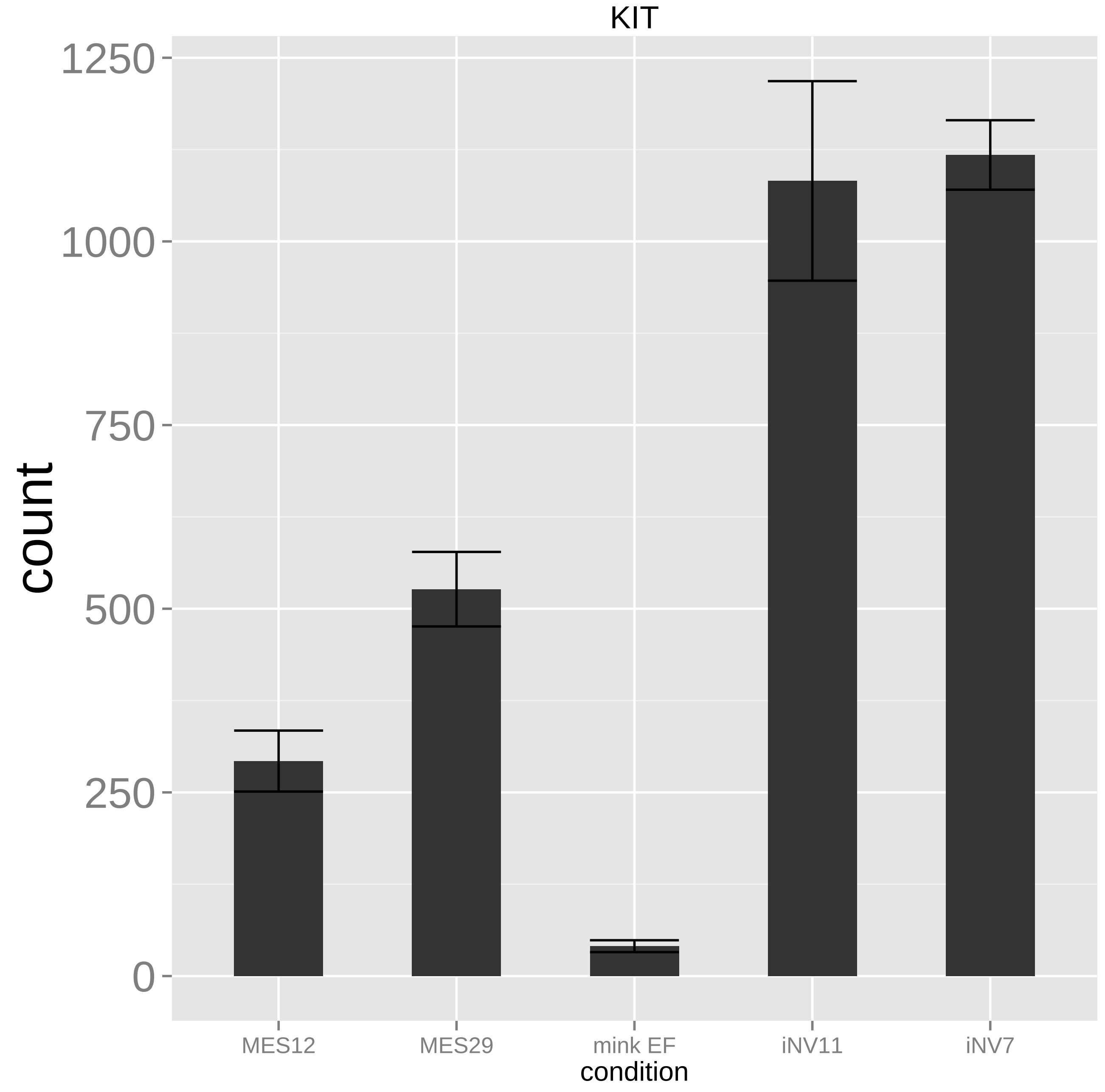
KIT
1250
1000
750
500
250
0
count
MES12
MES29
mink EF
iNV11
iNV7
condition

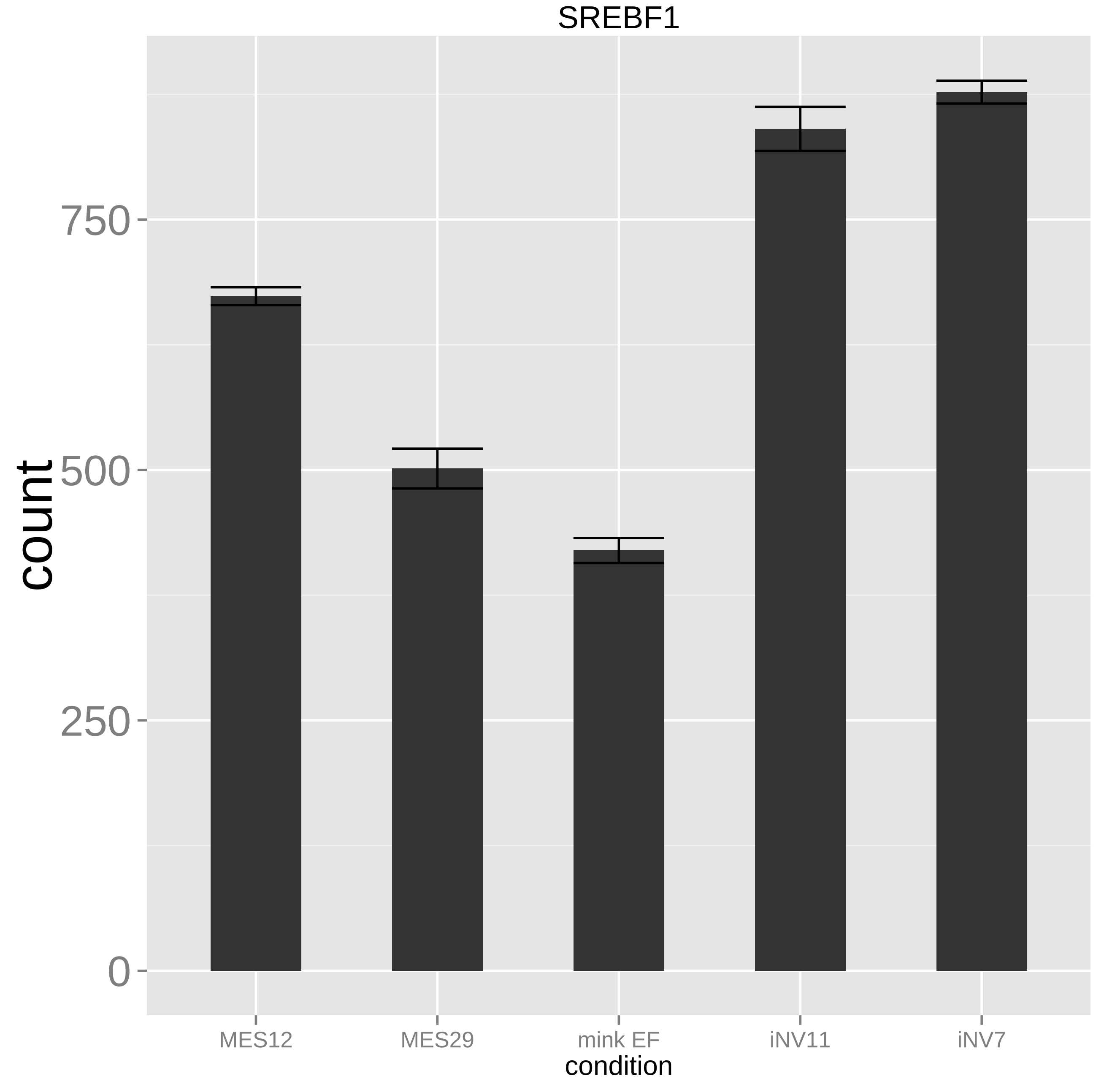

SREBF1
count
750
500
250
0
MES12
MES29
mink EF
iNV11
iNV7
condition

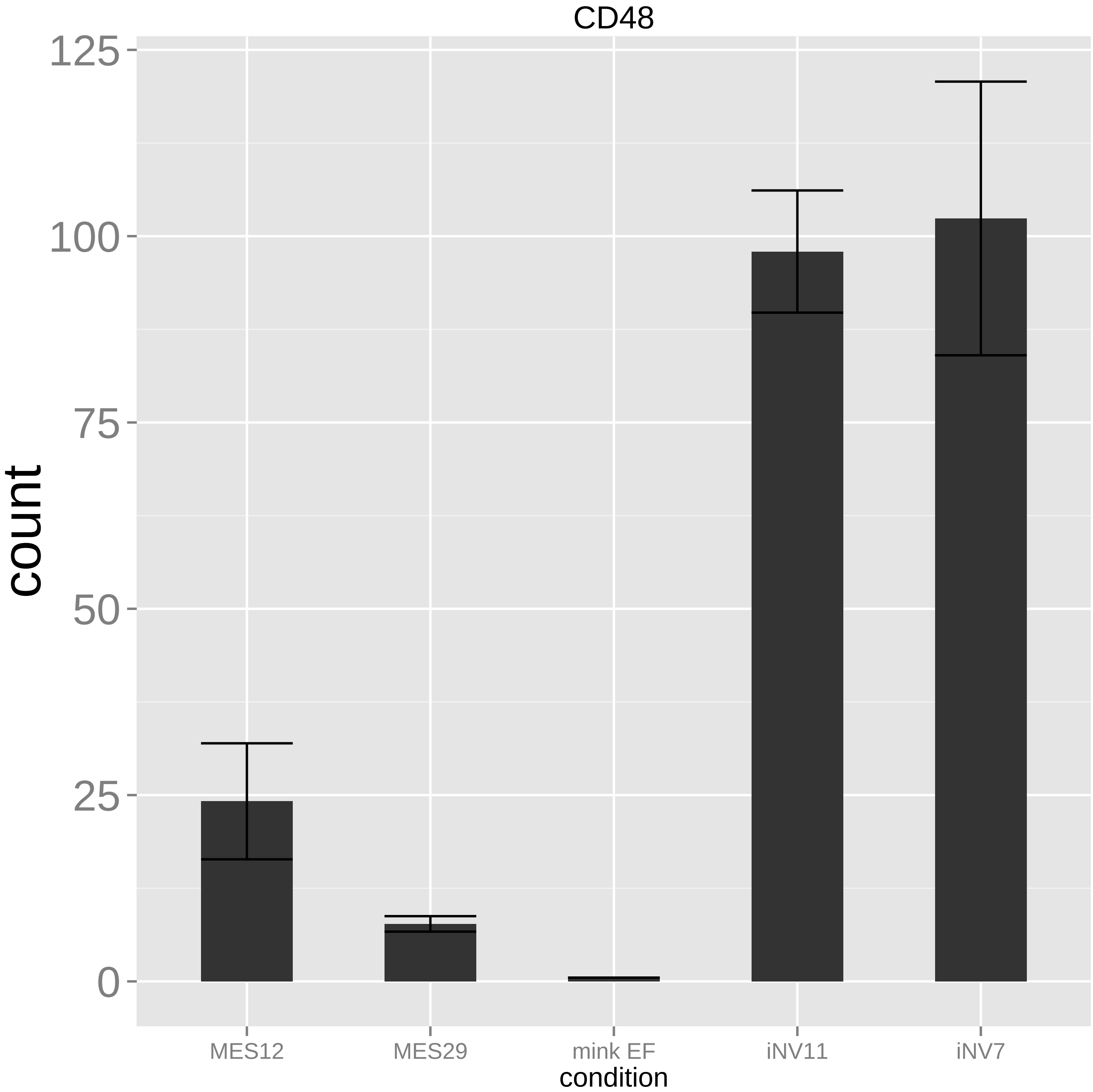

CD48
count
125
100
75
50
25
0
MES12
MES29
mink EF
iNV11
iNV7
condition

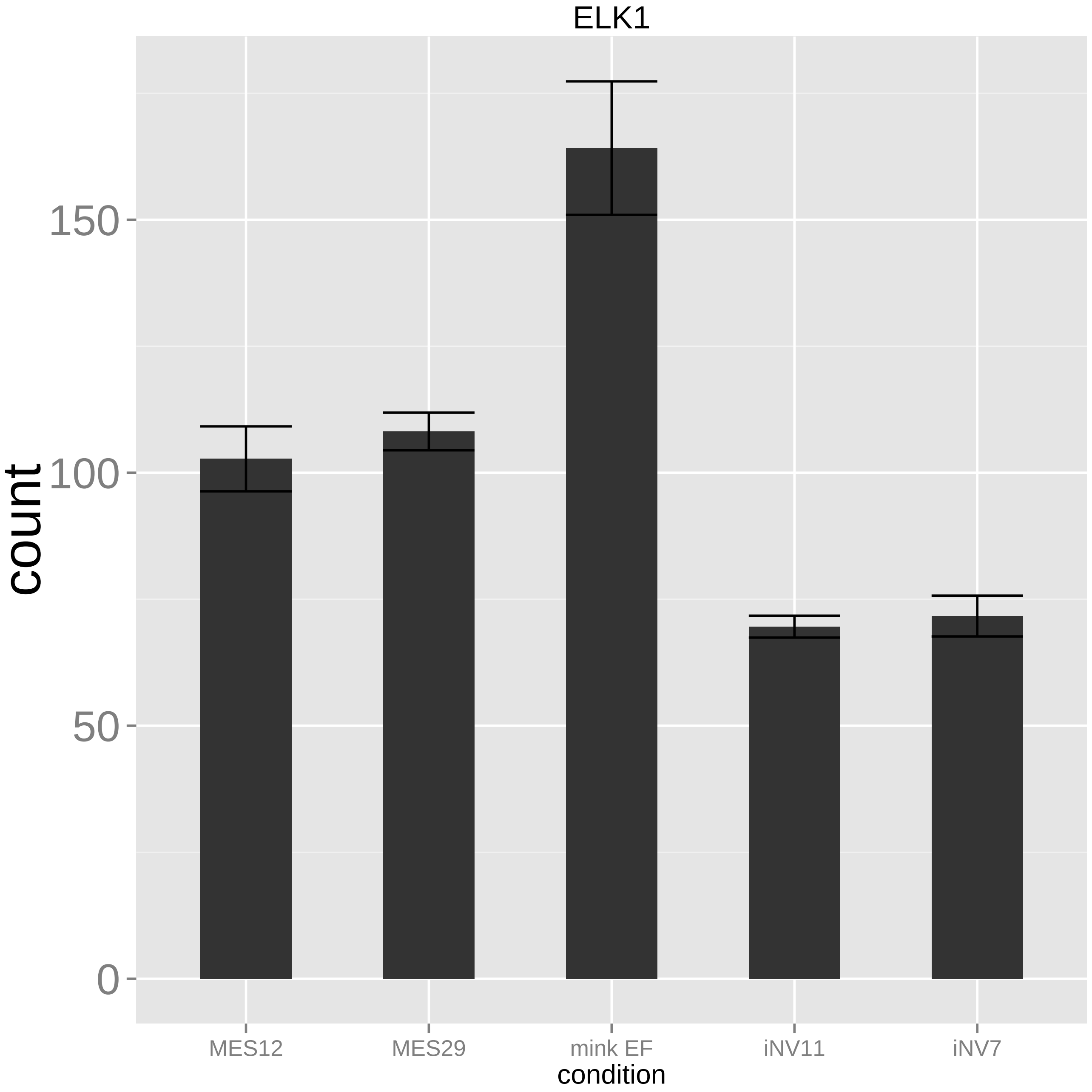

ELK1
count
150
100
50
0
MES12
MES29
mink EF
iNV11
iNV7
condition

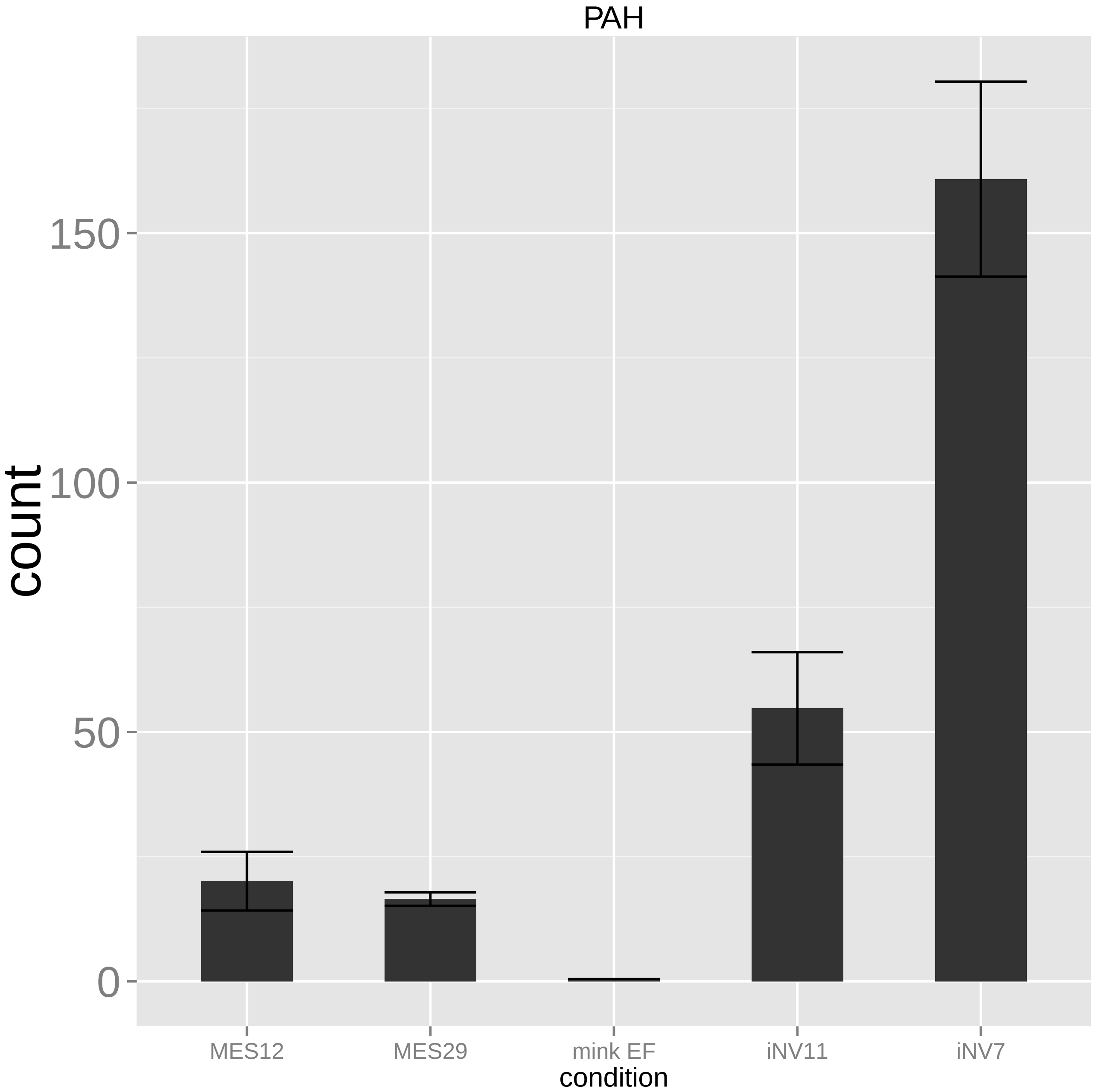
PAH
count
150
100
50
0
MES12
MES29
mink EF
iNV11
iNV7
condition

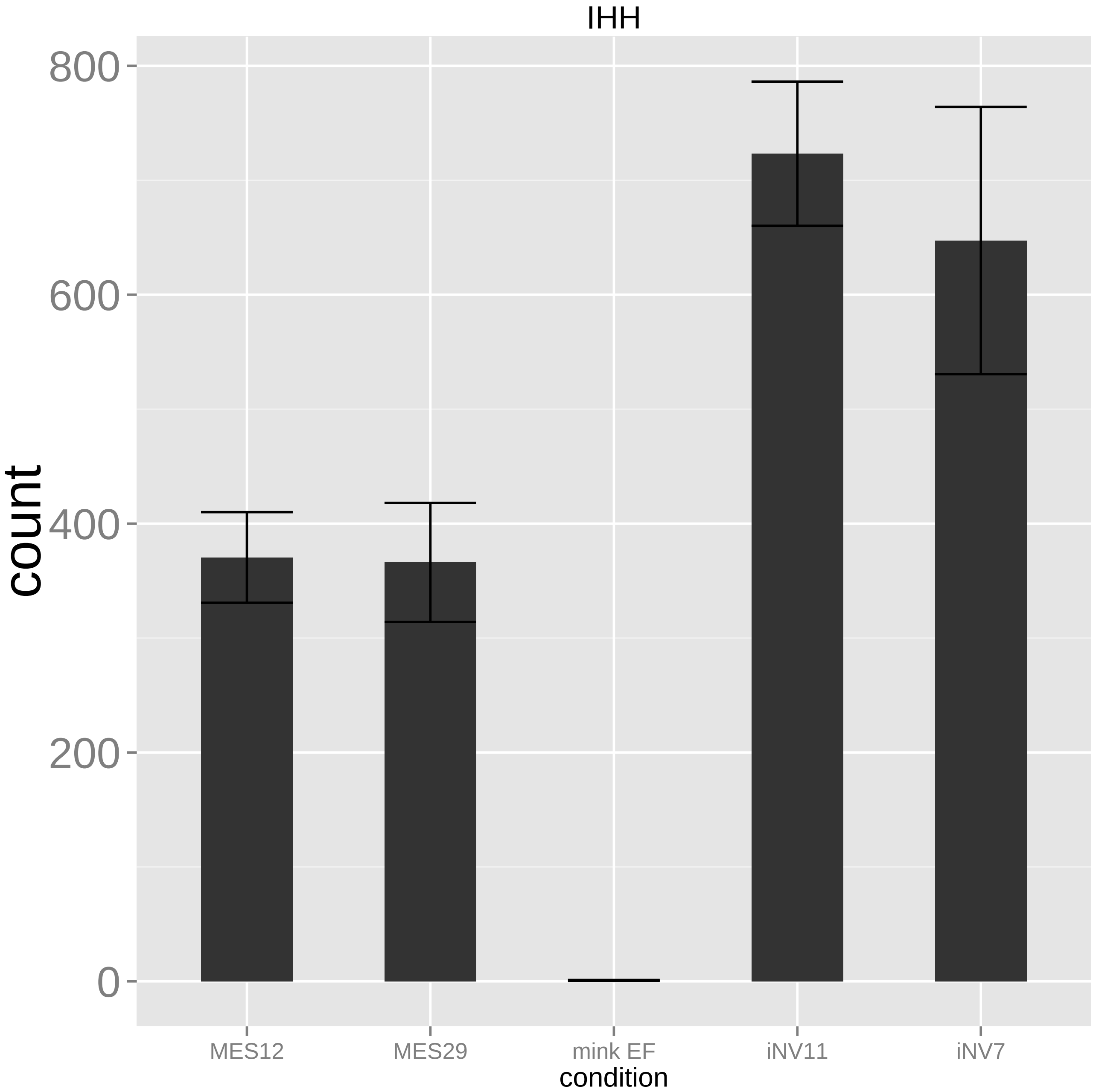

IHH
800
600
400
200
0
count
MES12
MES29
mink EF
iNV11
iNV7
condition

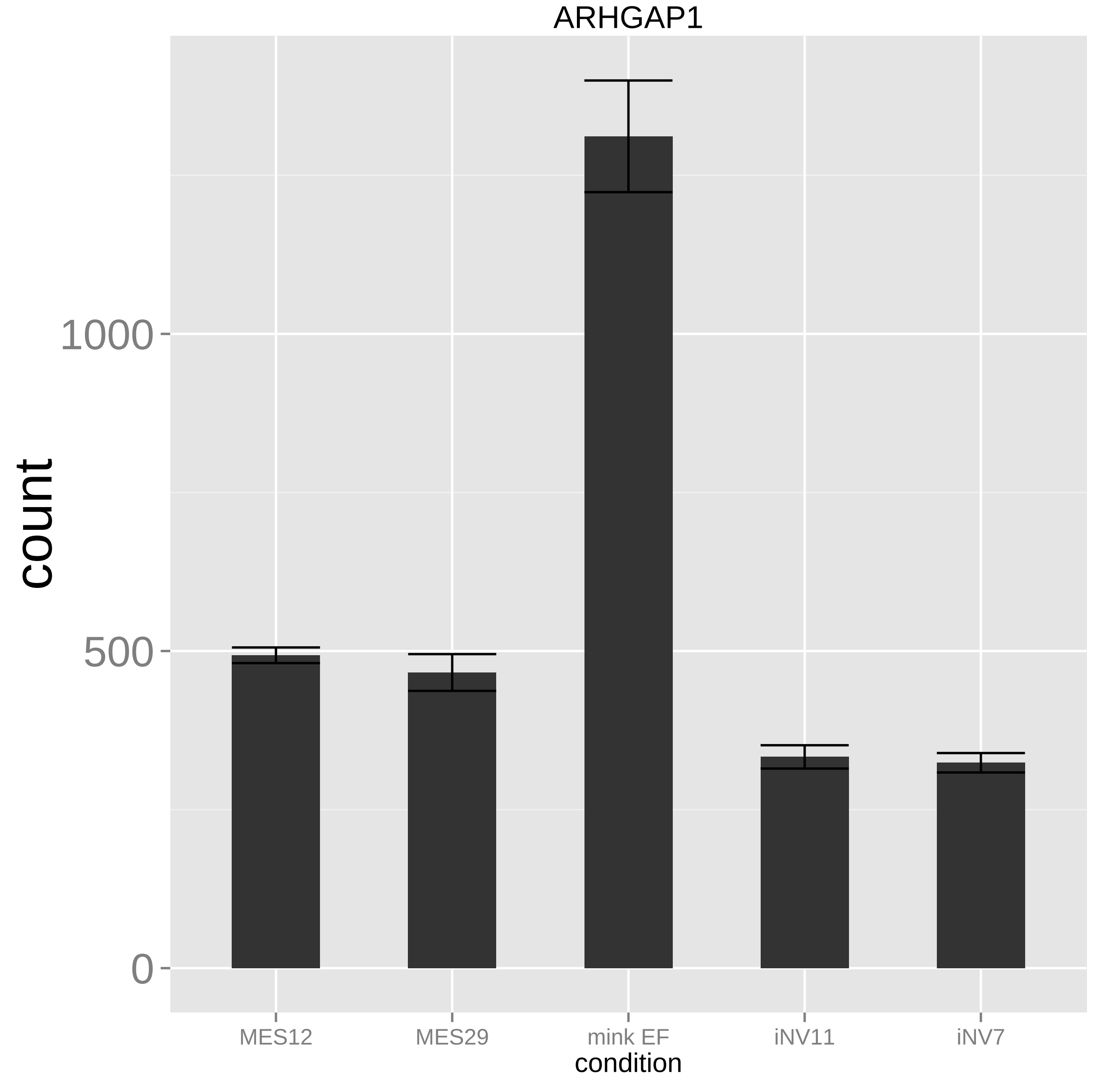

ARHGAP1
count
1000
500
0
MES12
MES29
mink EF
iNV11
iNV7
condition

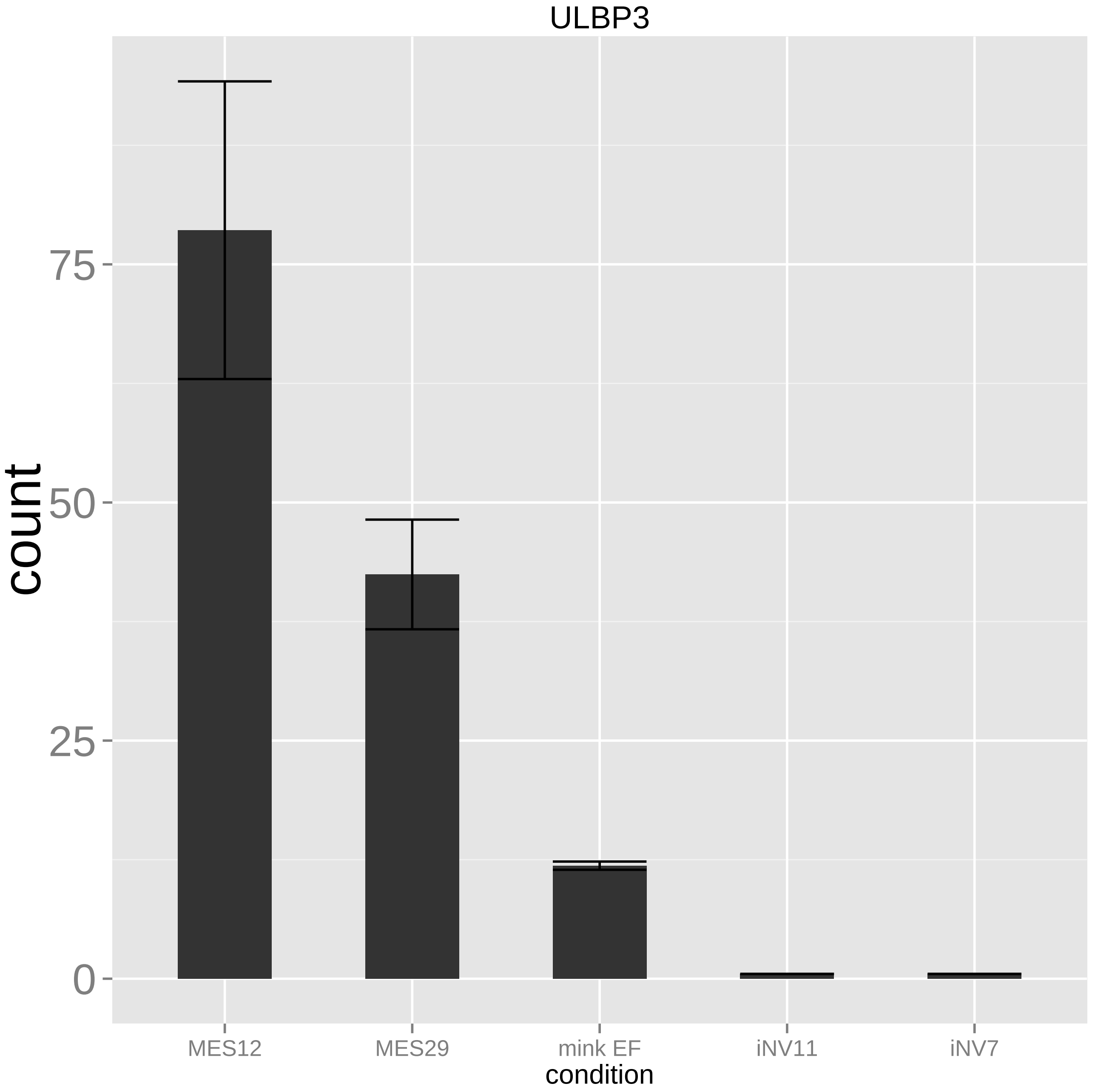
ULBP3
count
75
50
25
0
MES12
MES29
mink EF
iNV11
iNV7
condition

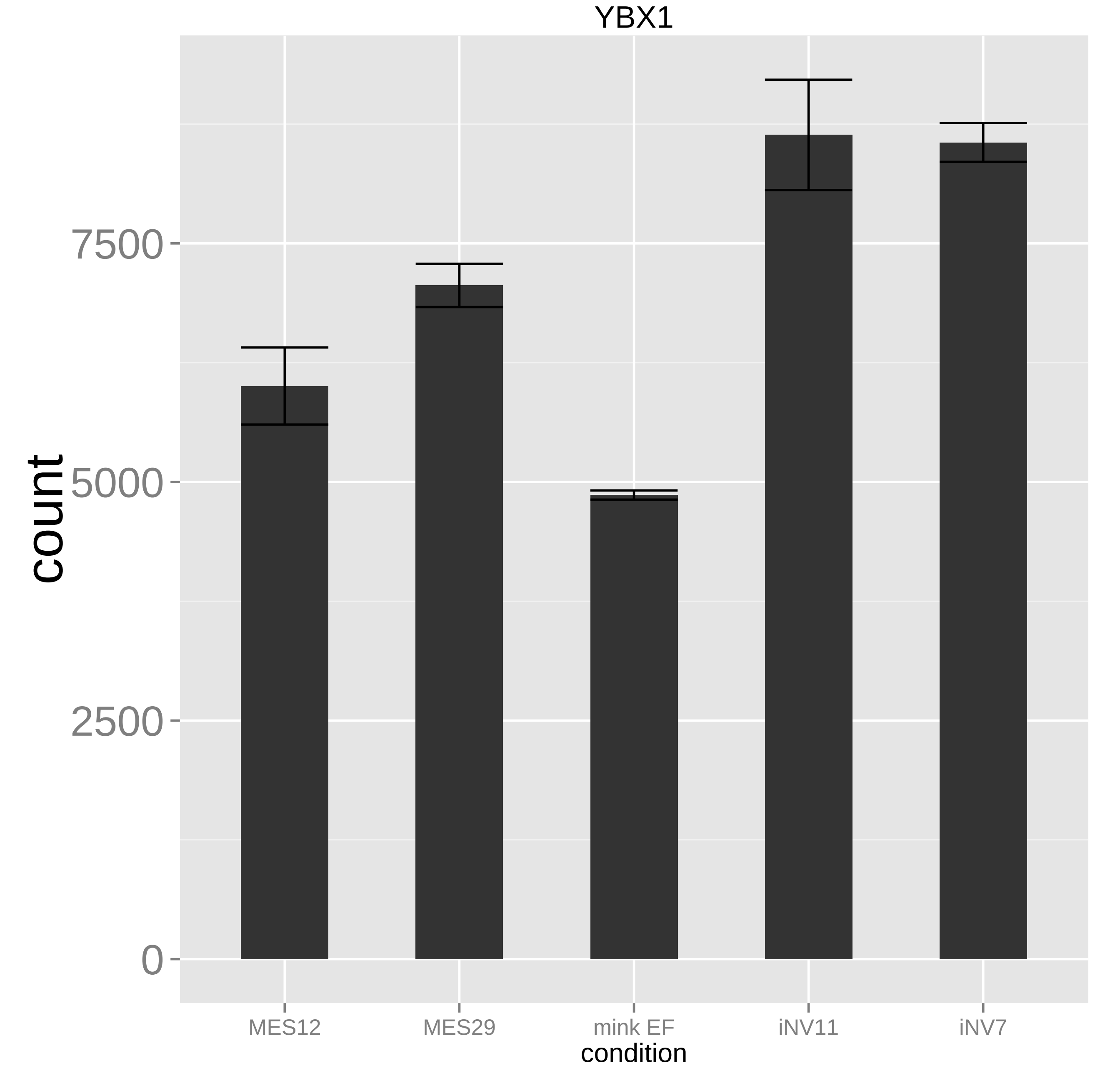
YBX1
count
7500
5000
2500
0
MES12
MES29
mink EF
iNV11
iNV7
condition

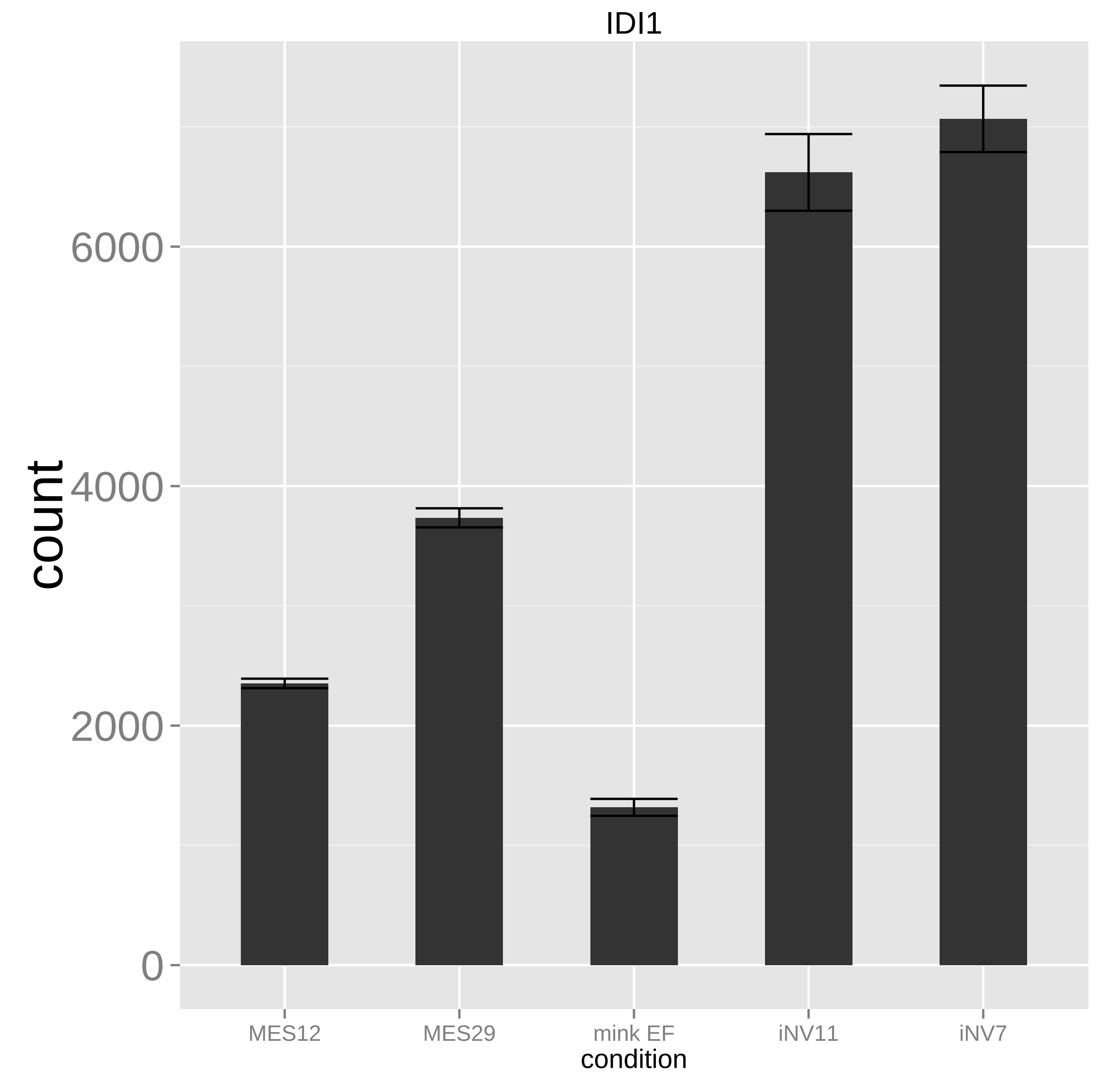
IDI1
count
6000
4000
2000
0
MES12
MES29
mink EF
iNV11
iNV7
condition

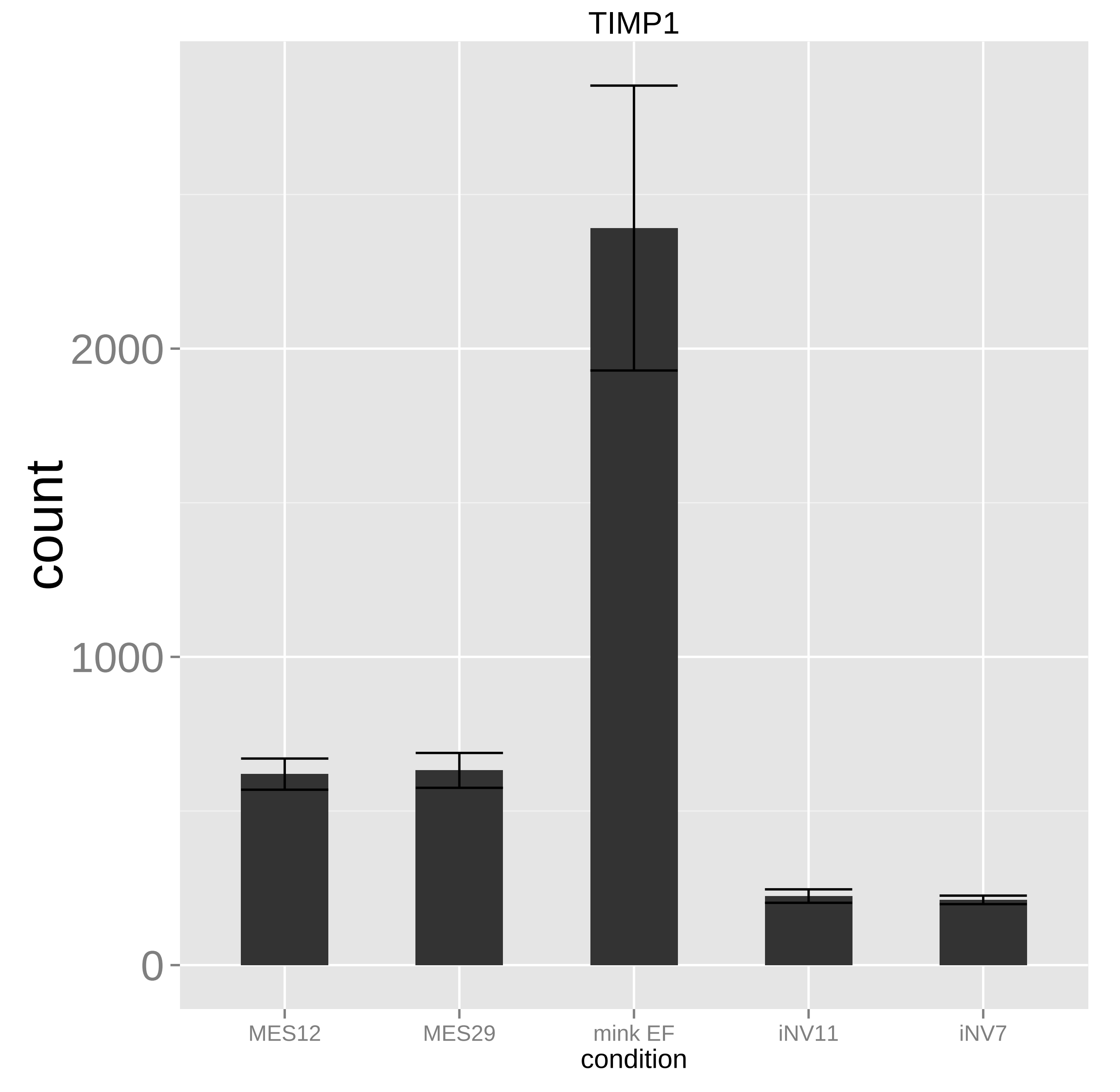
TIMP1
count
2000
1000
0
MES12
MES29
mink EF
iNV11
iNV7
condition

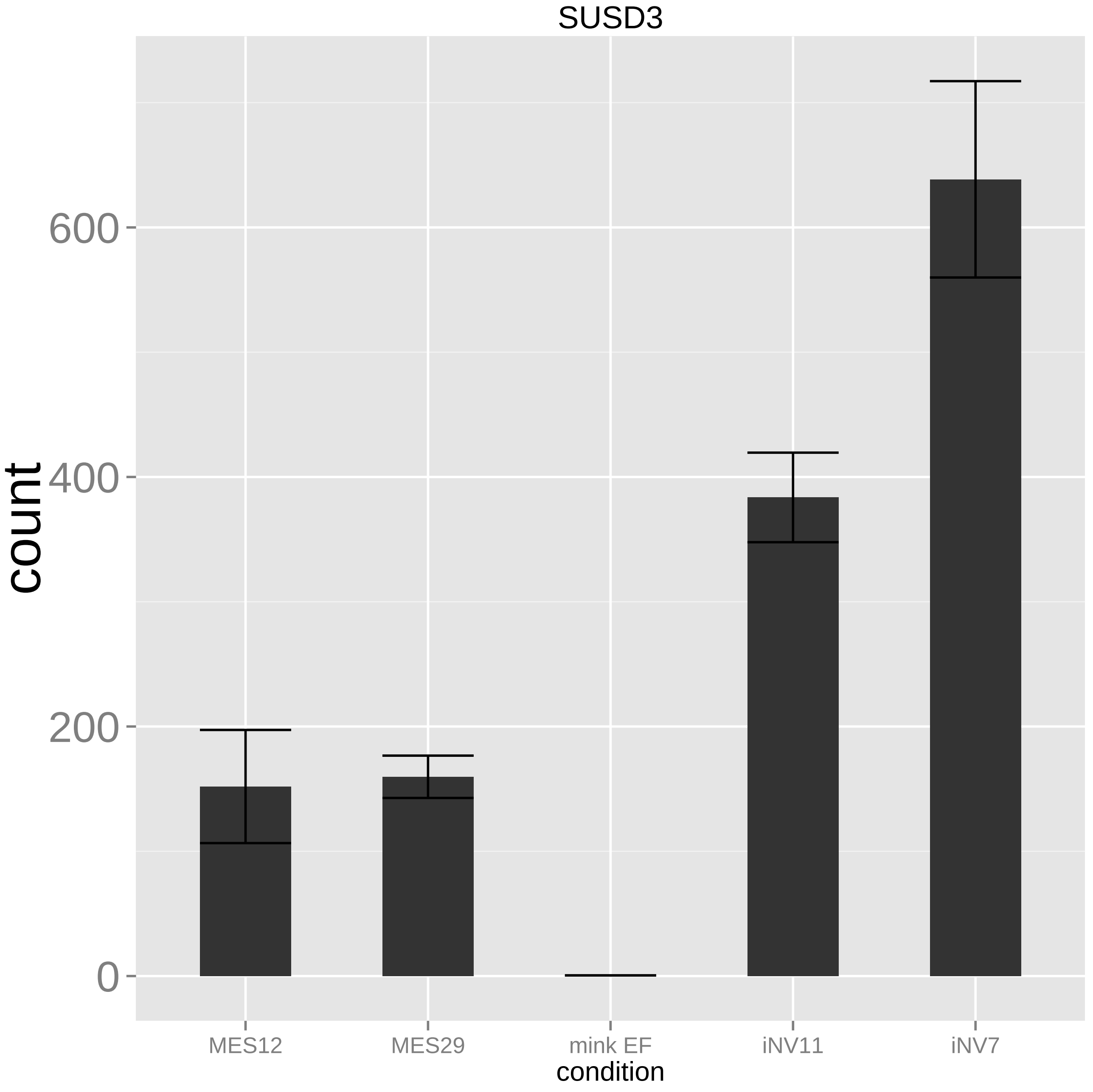

SUSD3
count
600
400
200
0
MES12
MES29
mink EF
iNV11
iNV7
condition

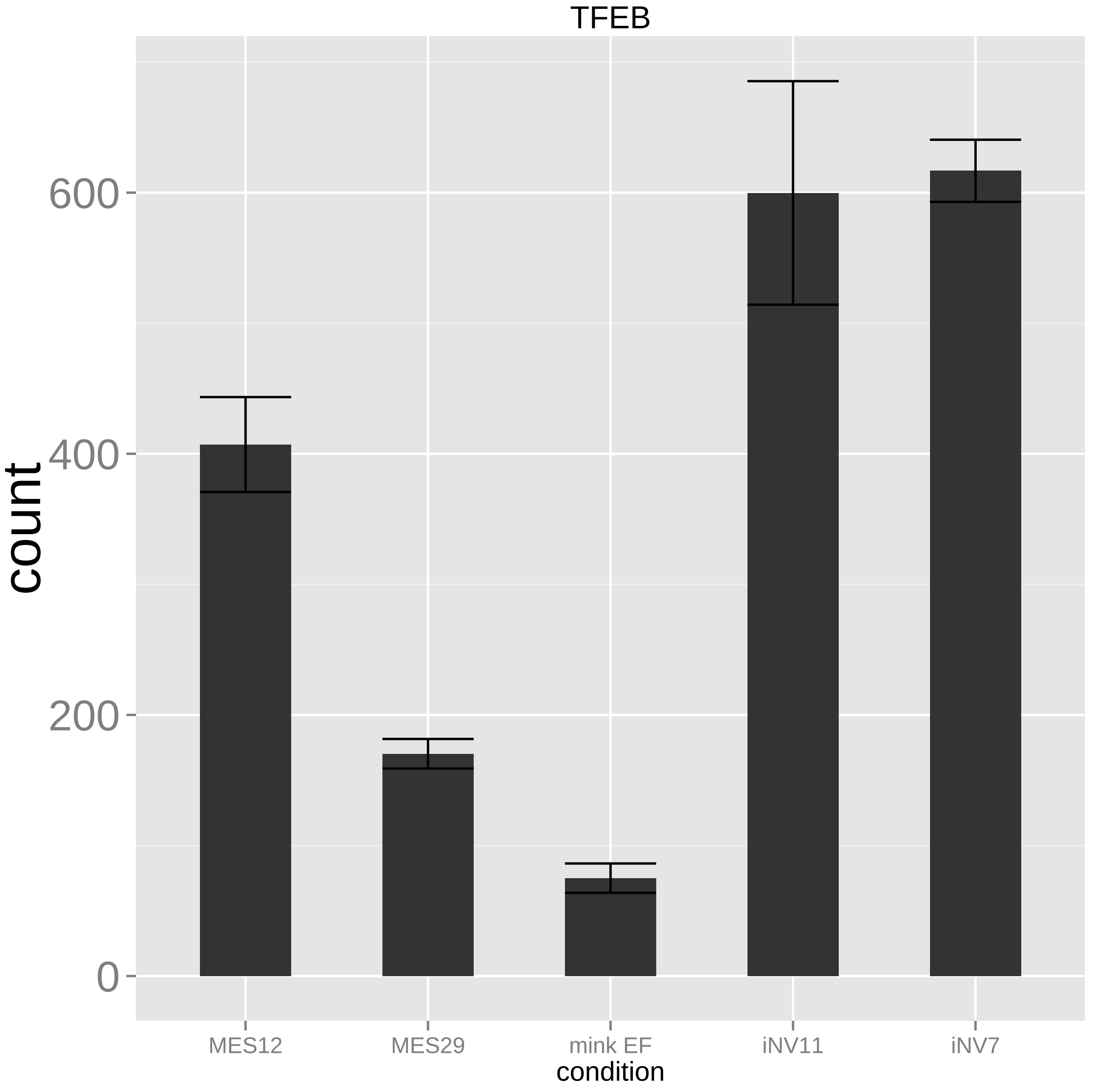
TFEB
count
600
400
200
0
MES12
MES29
mink EF
iNV11
iNV7
condition

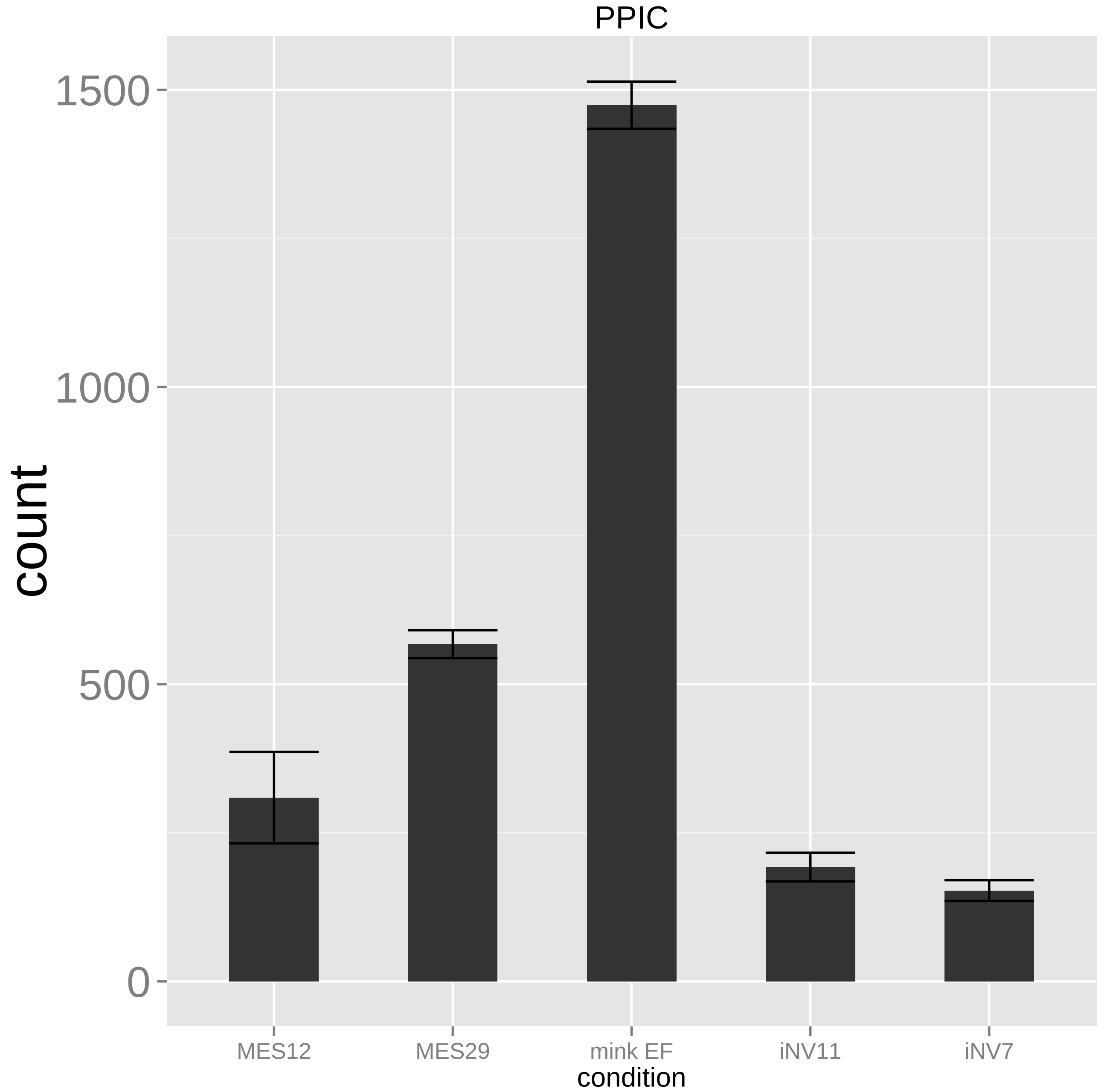
PPIC
count
1500
1000
500
0
MES12
MES29
mink EF
iNV11
iNV7
condition

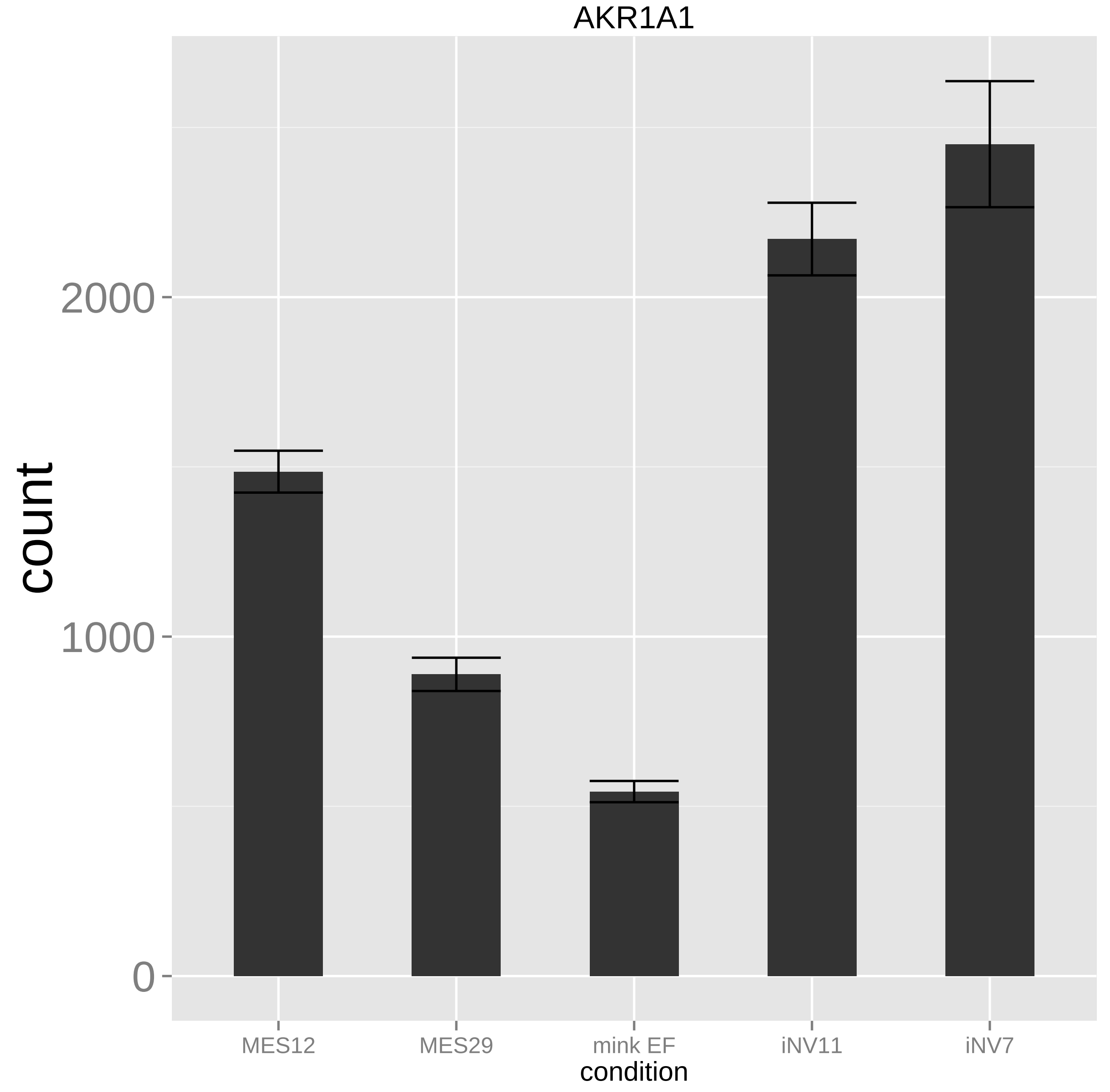
AKR1A1
count
2000
1000
0
MES12
MES29
mink EF
iNV11
iNV7
condition

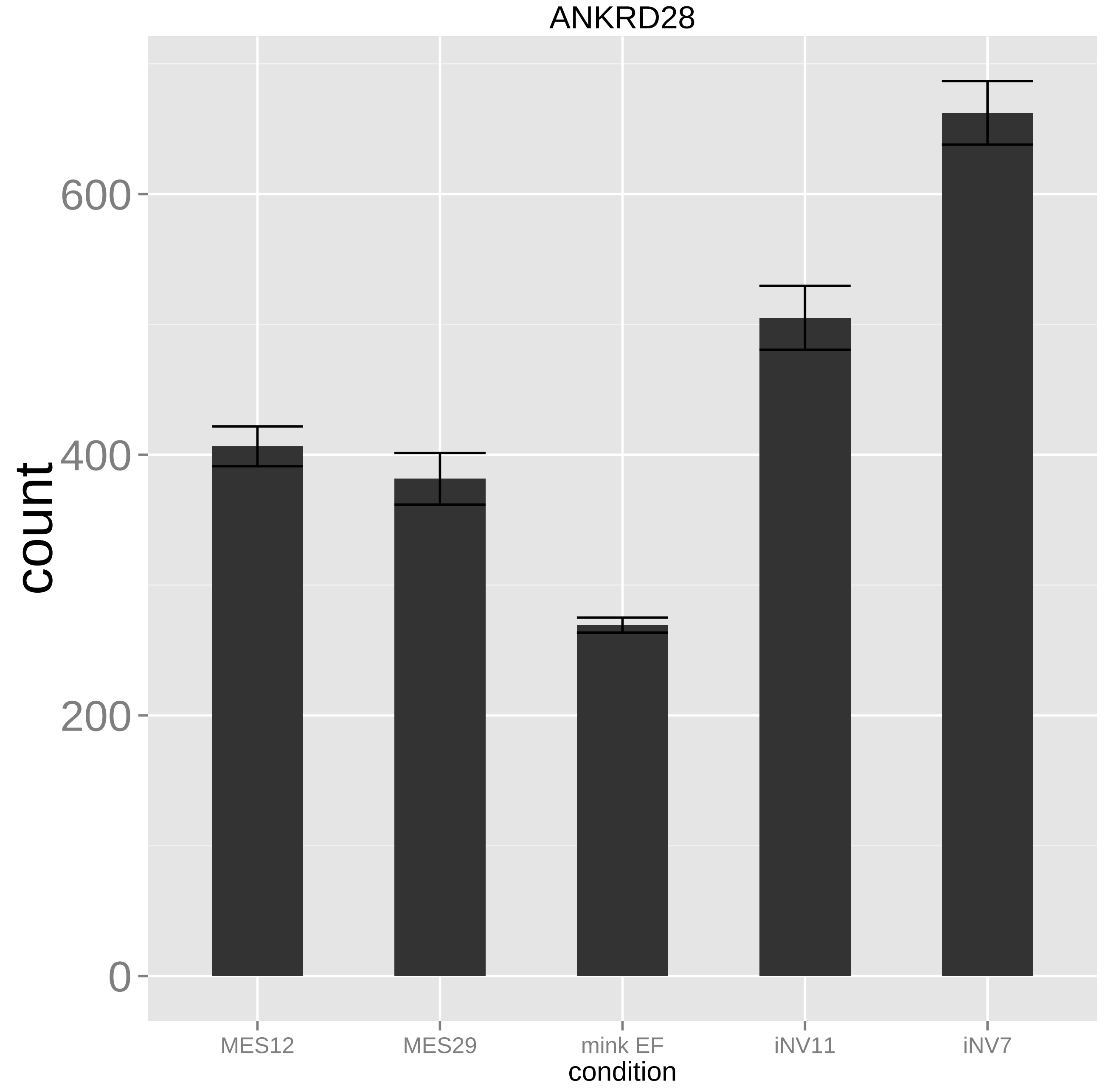

ANKRD28
count
600
400
200
0
MES12
MES29
mink EF
iNV11
iNV7
condition

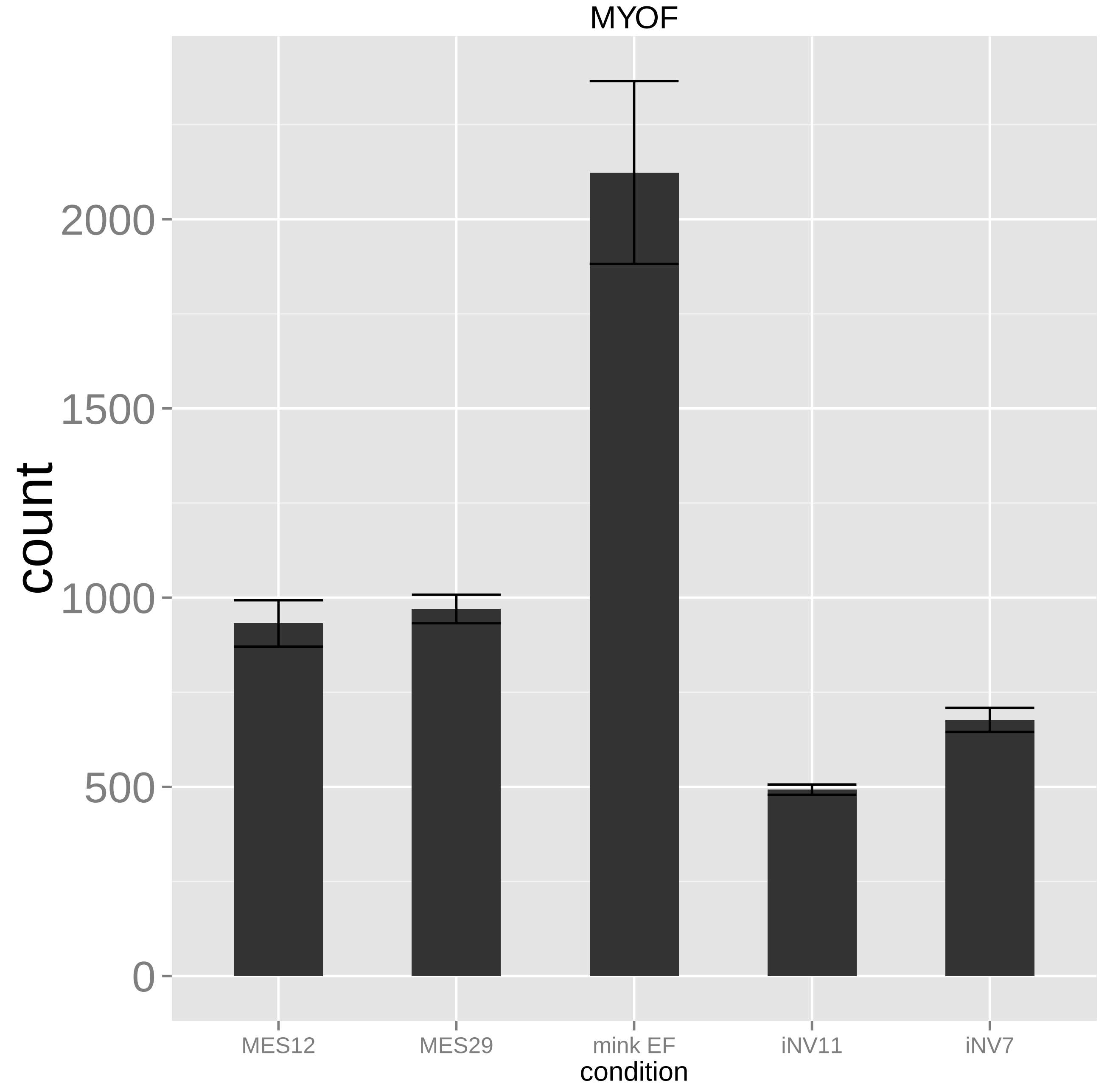
MYOF
count
2000
1500
1000
500
0
MES12
MES29
mink EF
iNV11
iNV7
condition

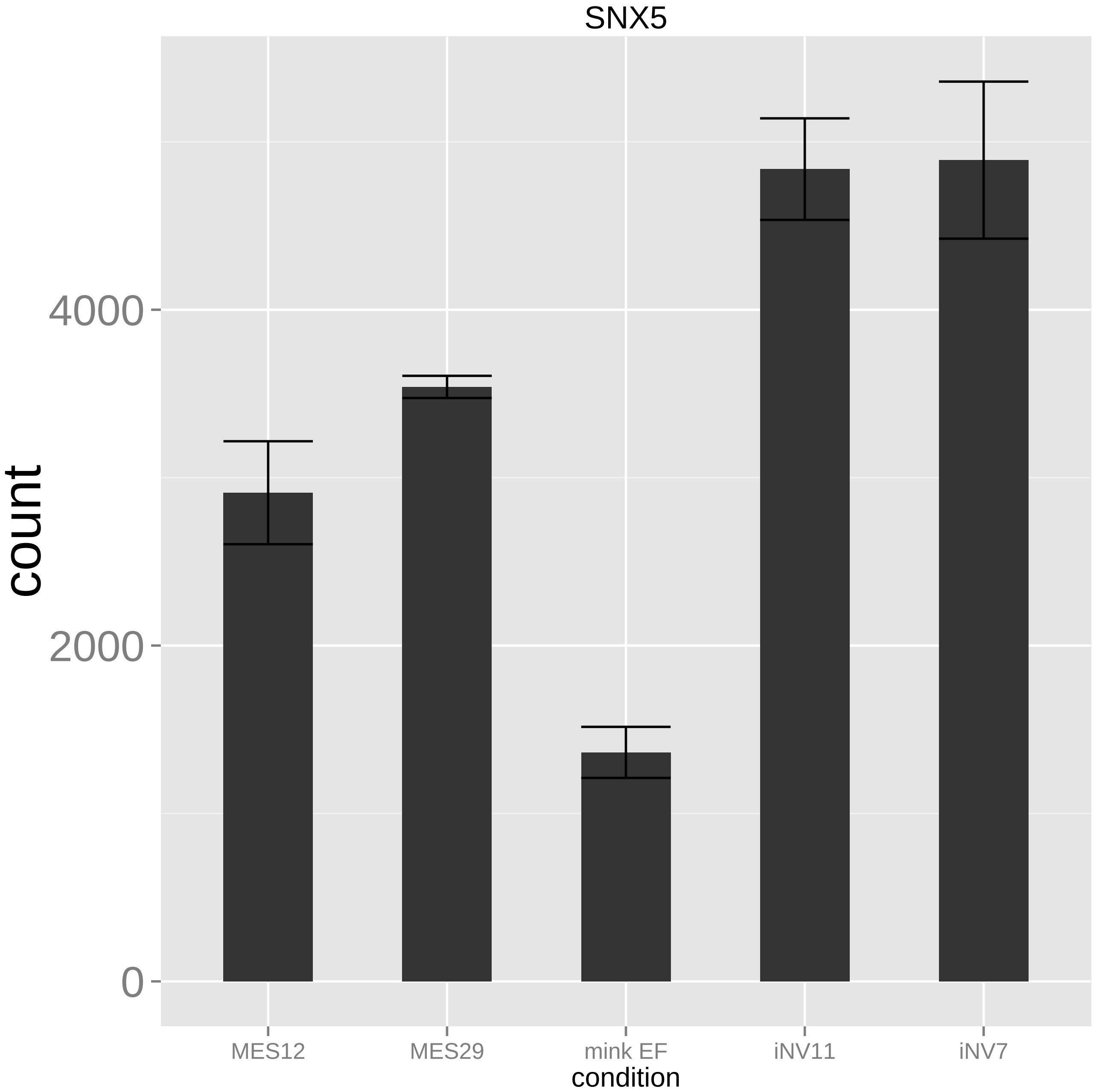
SNX5
count
4000
2000
0
MES12
MES29
mink EF
iNV11
iNV7
condition

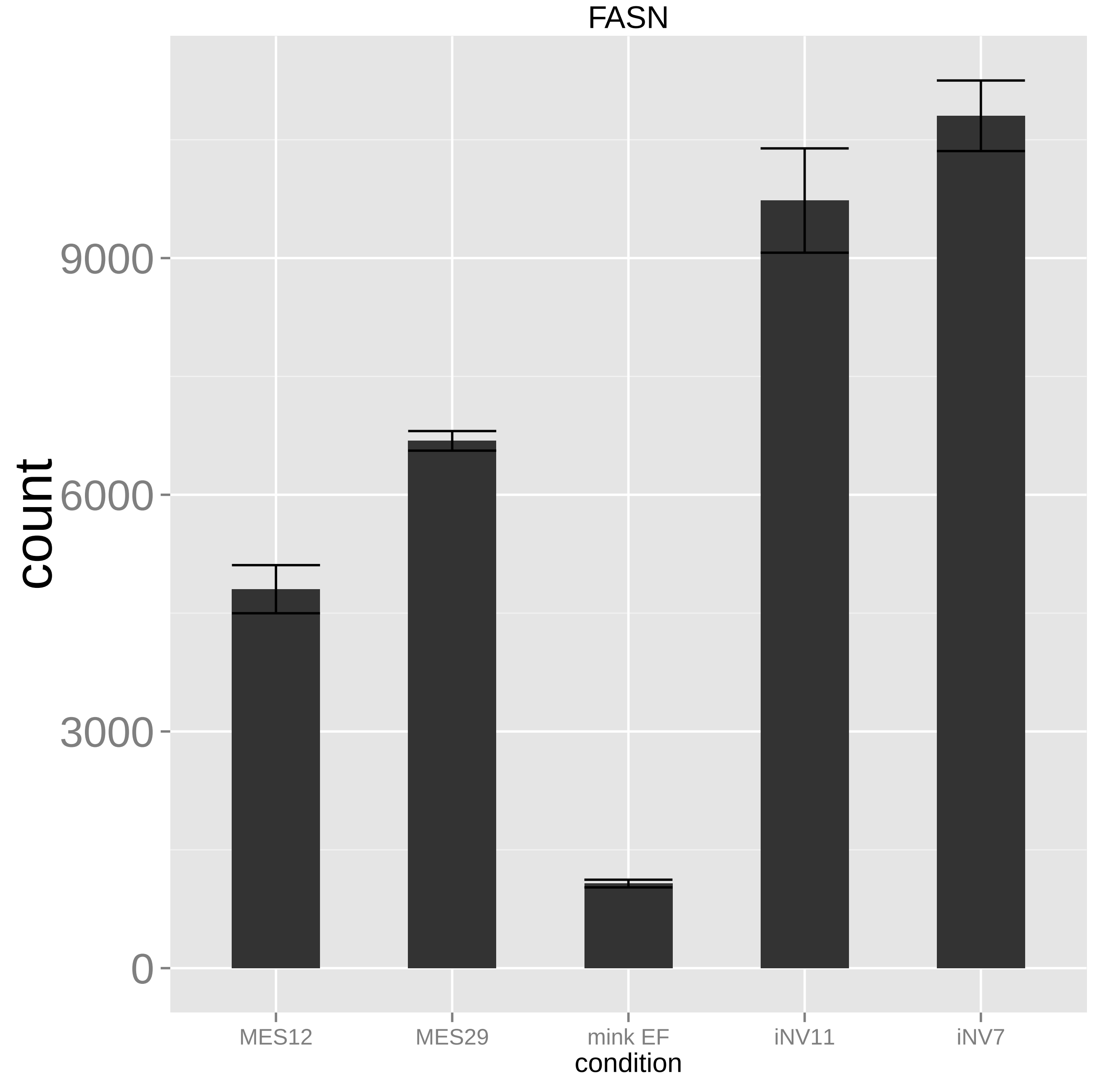
FASN
count
9000
6000
3000
0
MES12
MES29
mink EF
iNV11
iNV7
condition

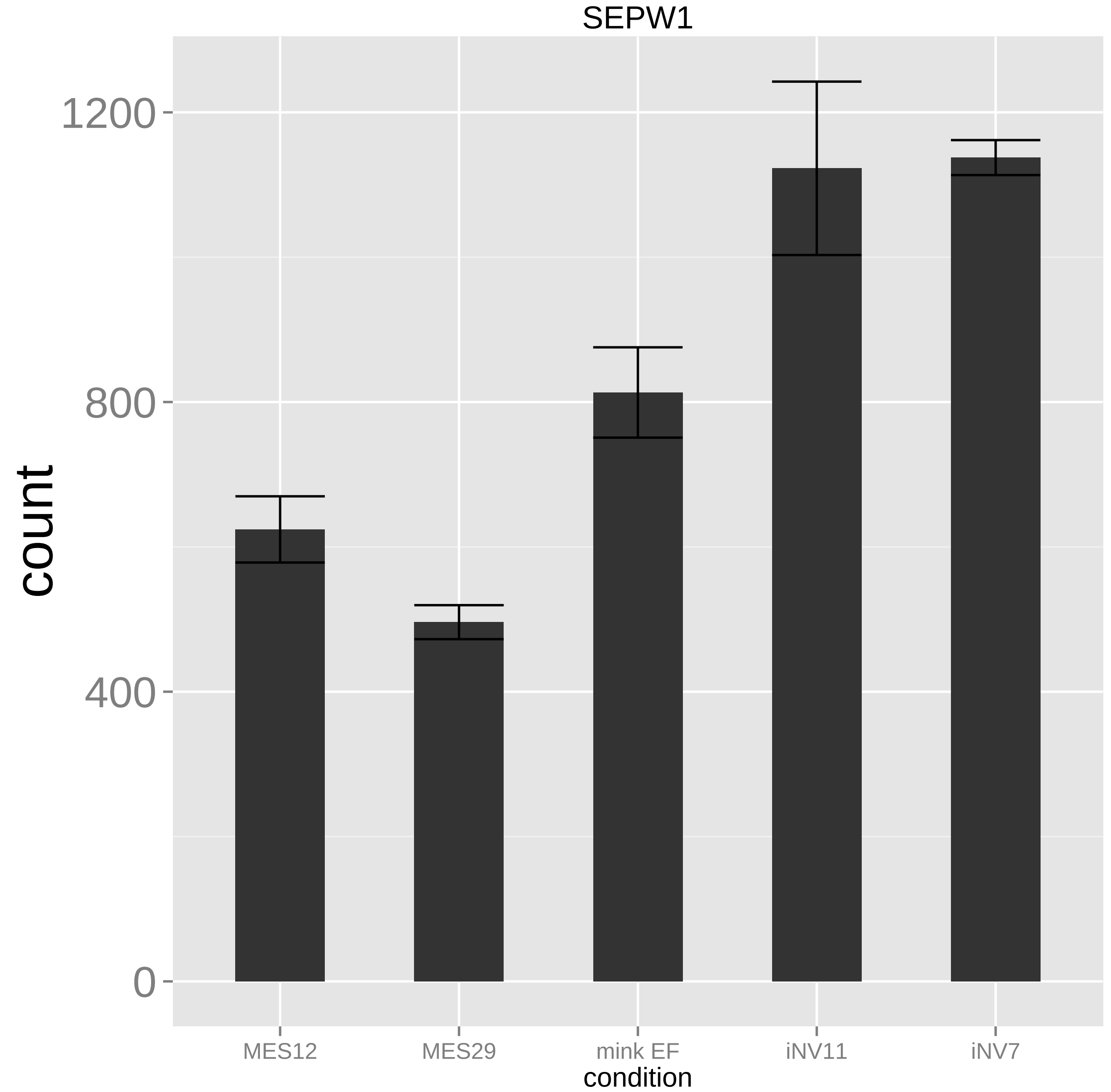
SEPW1
1200
800
400
0
count
MES12
MES29
mink EF
iNV11
iNV7
condition

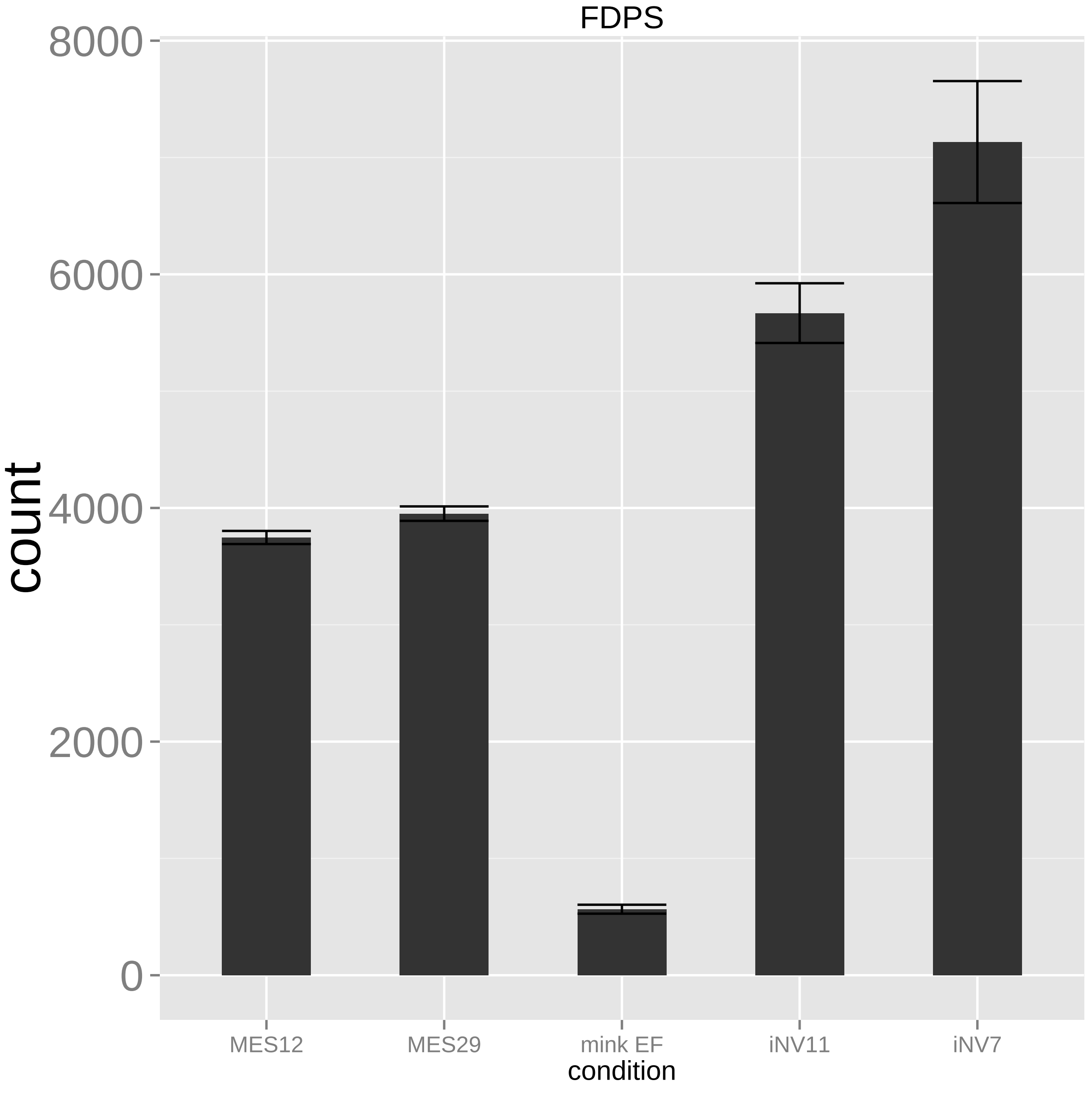
FDPS
8000
6000
4000
2000
0
count
MES12
MES29
mink EF
iNV11
iNV7
condition

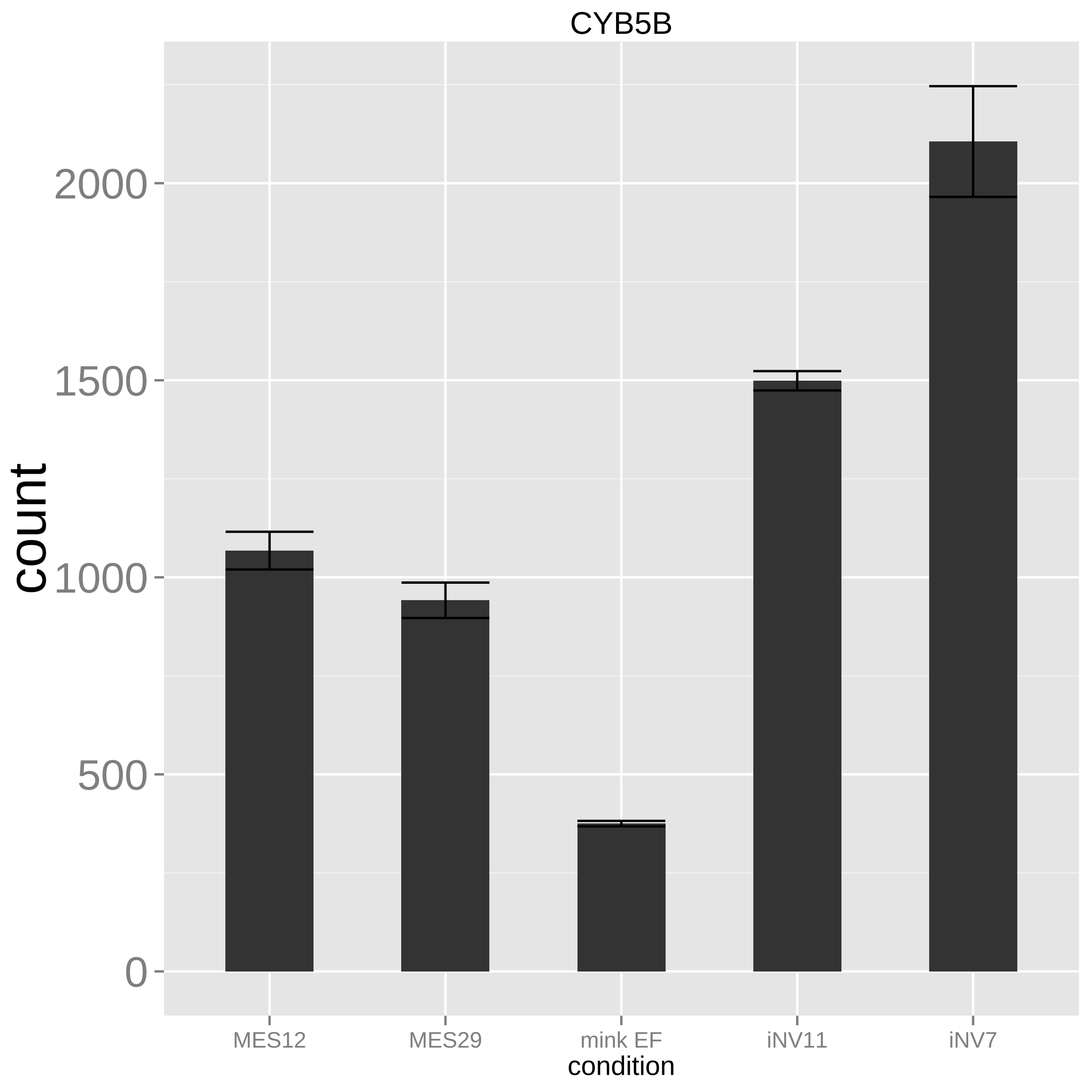
CYB5B
count
2000
1500
1000
500
0
MES12
MES29
mink EF
iNV11
iNV7
condition

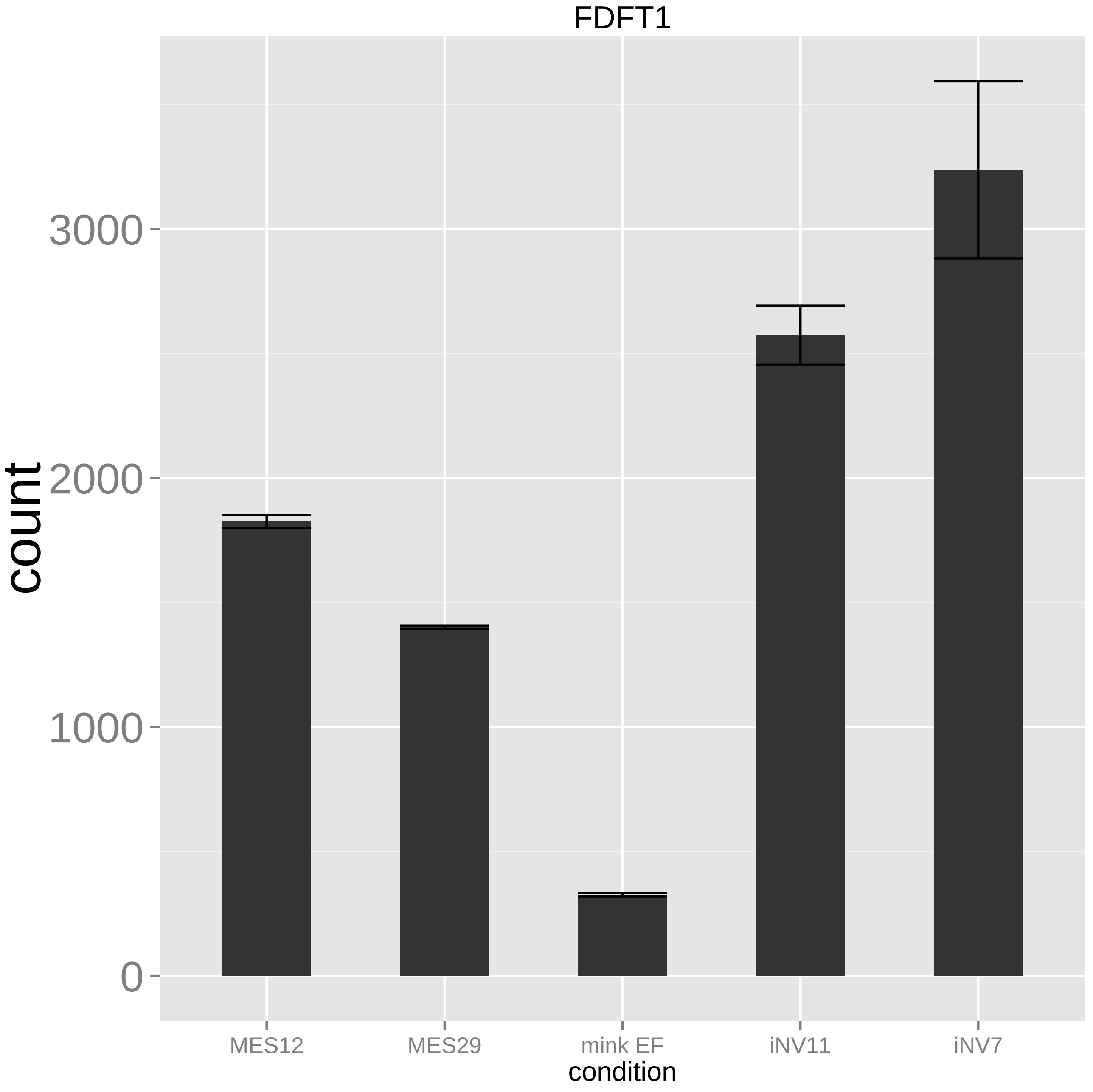
FDFT1
count
3000
2000
1000
0
MES12
MES29
mink EF
iNV11
iNV7
condition

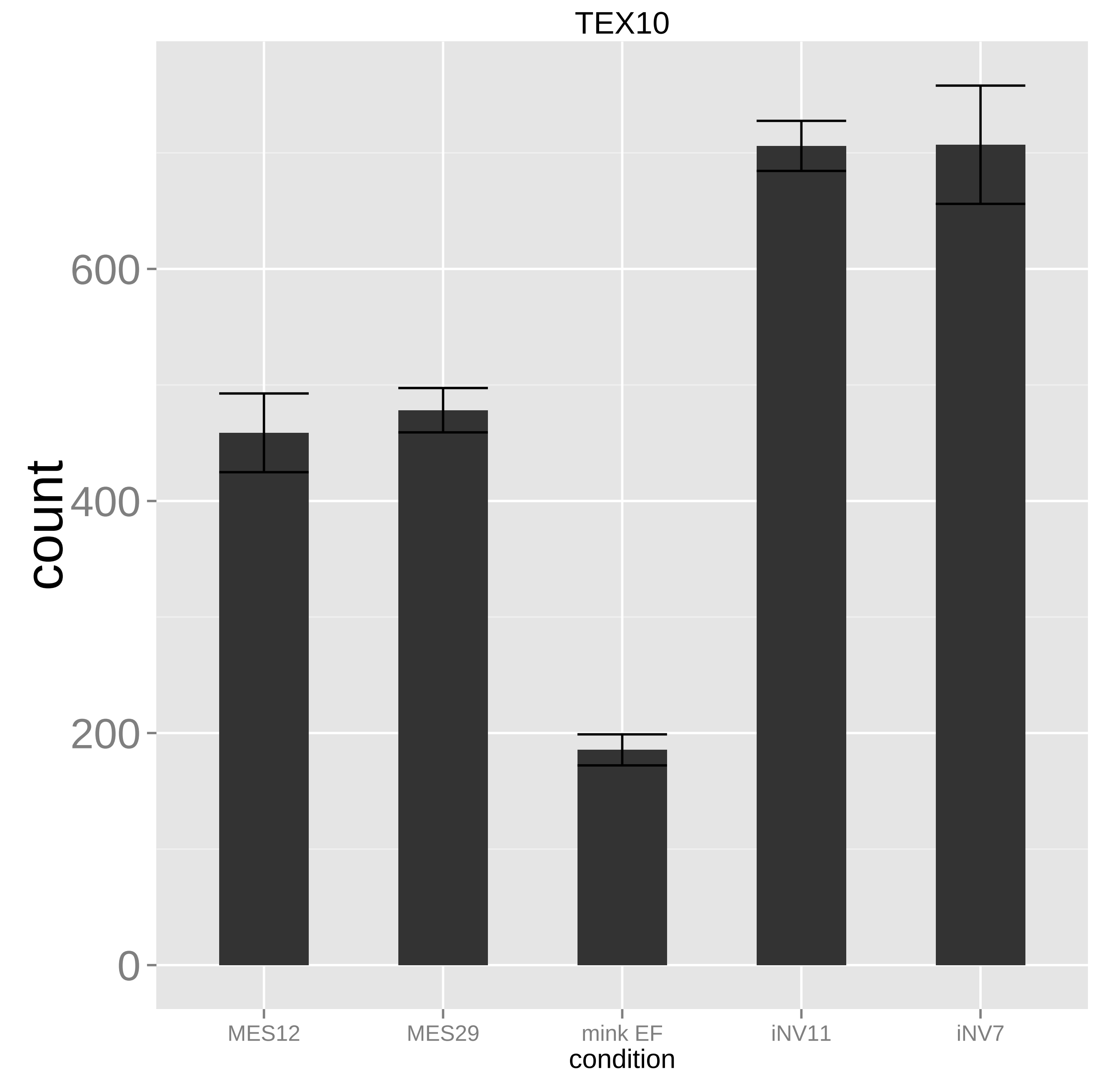
TEX10
count
600
400
200
0
MES12
MES29
mink EF
iNV11
iNV7
condition

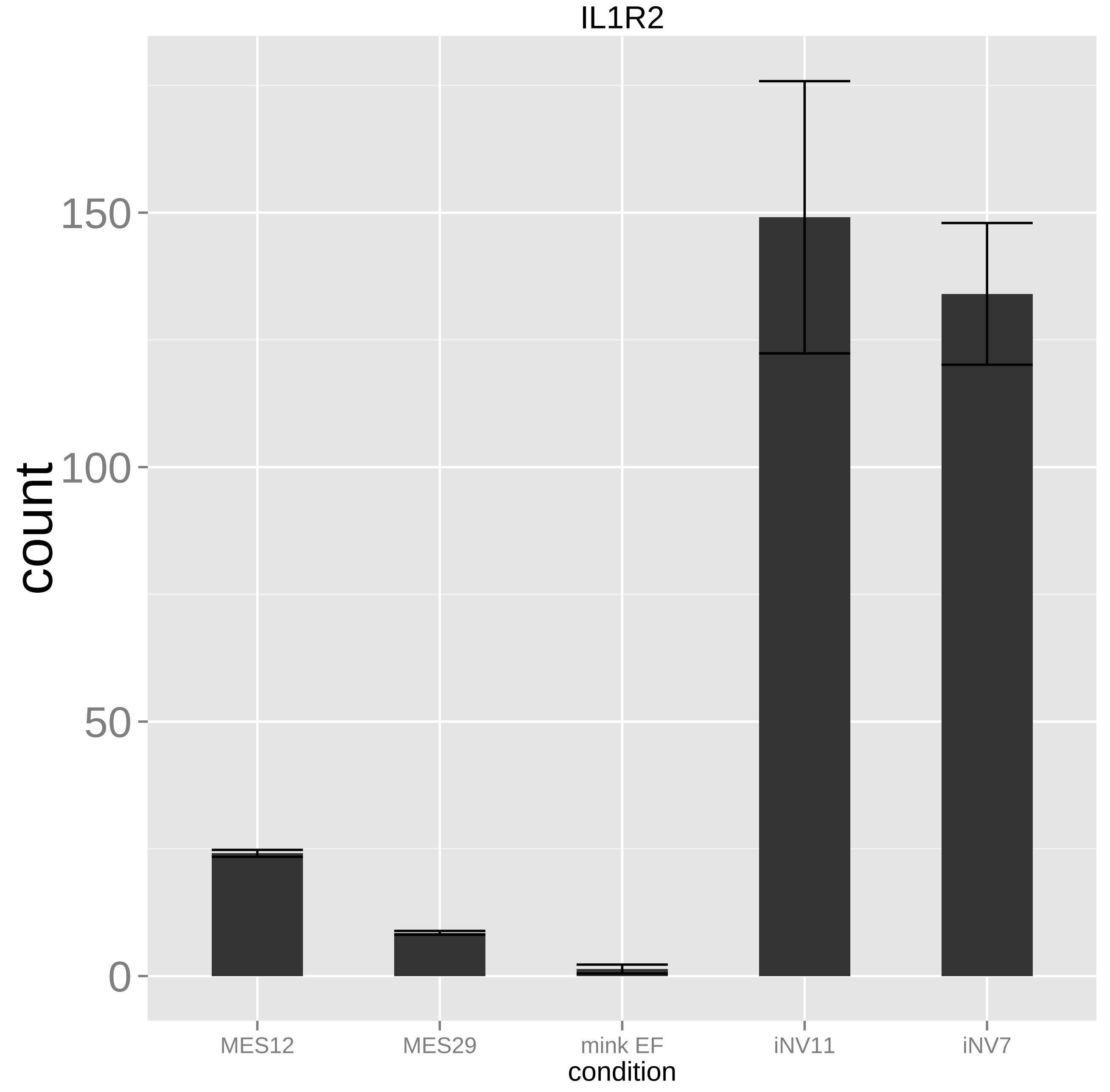
IL1R2
count
150
100
50
0
MES12
MES29
mink EF
iNV11
iNV7
condition

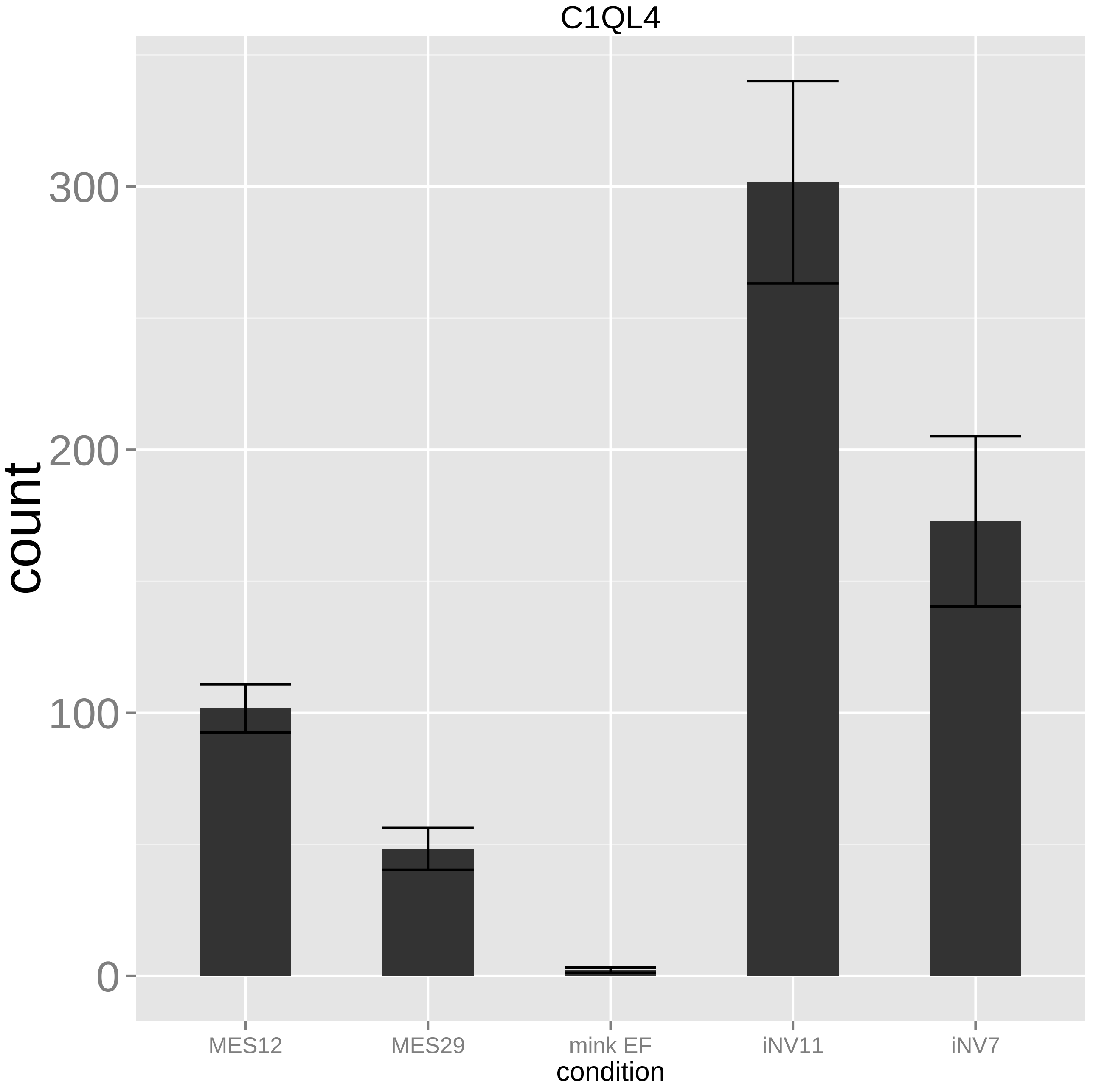
C1QL4
count
300
200
100
0
MES12
MES29
mink EF
iNV11
iNV7
condition

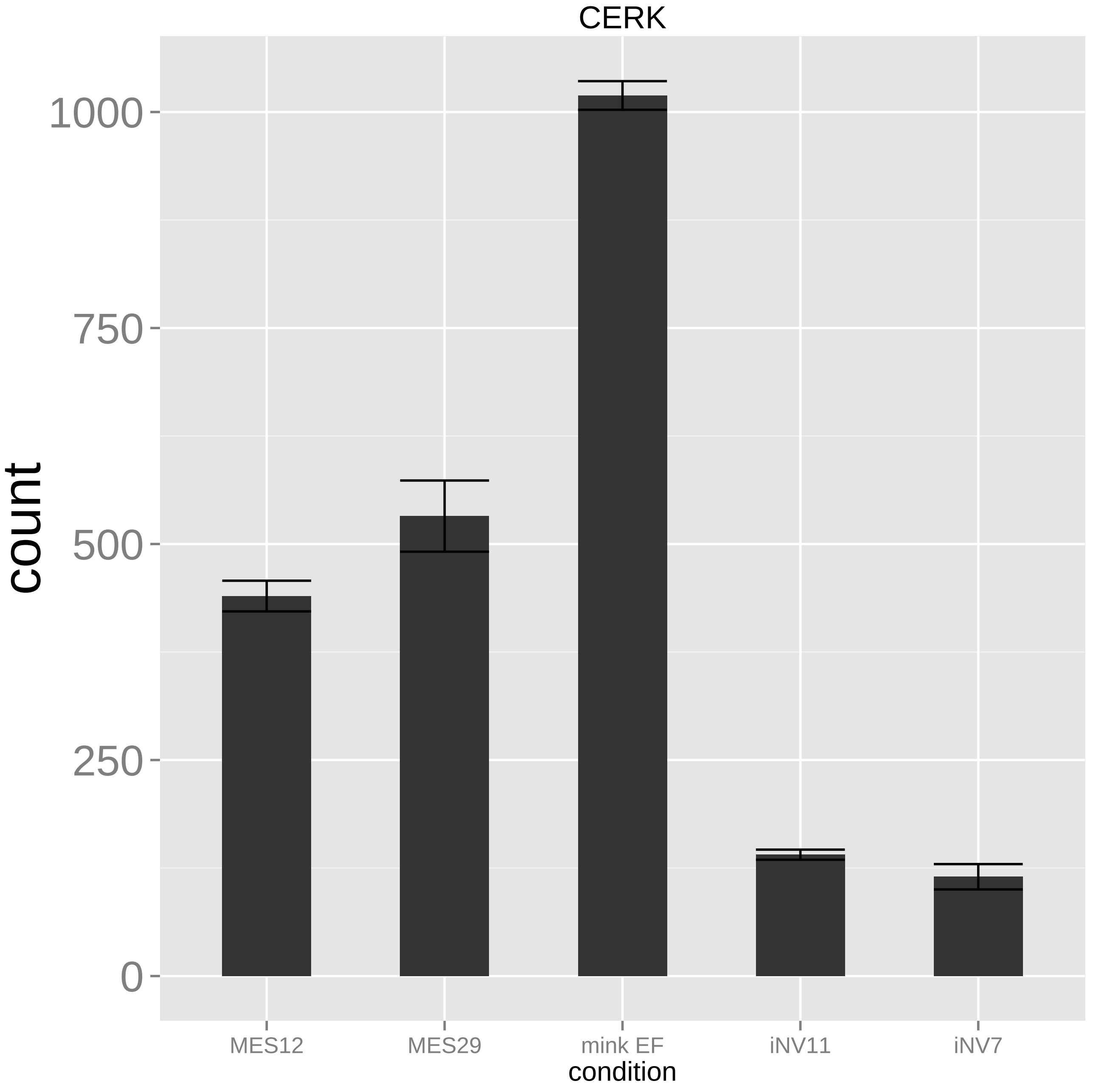
CERK
count
1000
750
500
250
0
MES12
MES29
mink EF
iNV11
iNV7
condition

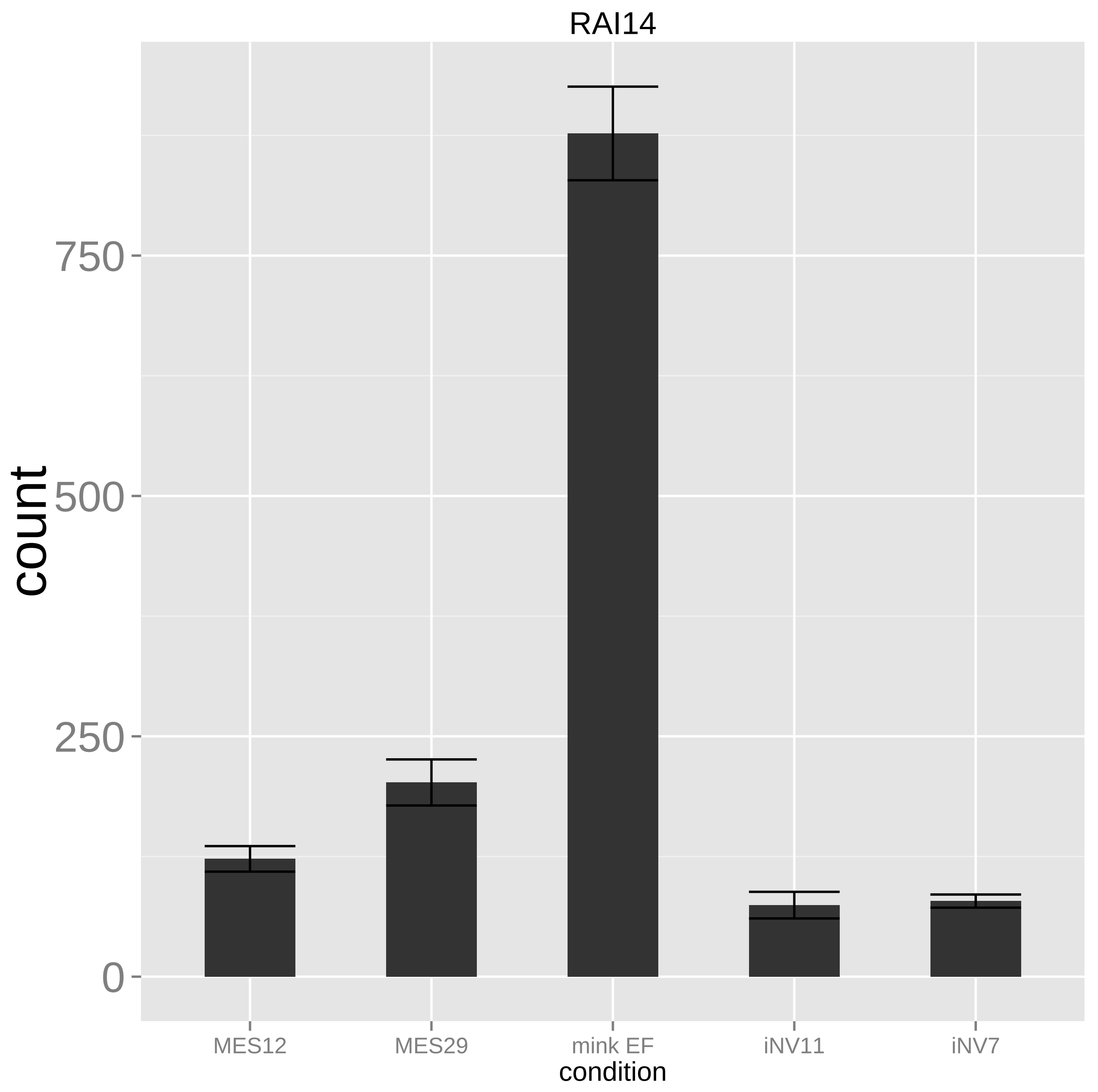

RAI14
count
750
500
250
0
MES12
MES29
mink EF
iNV11
iNV7
condition

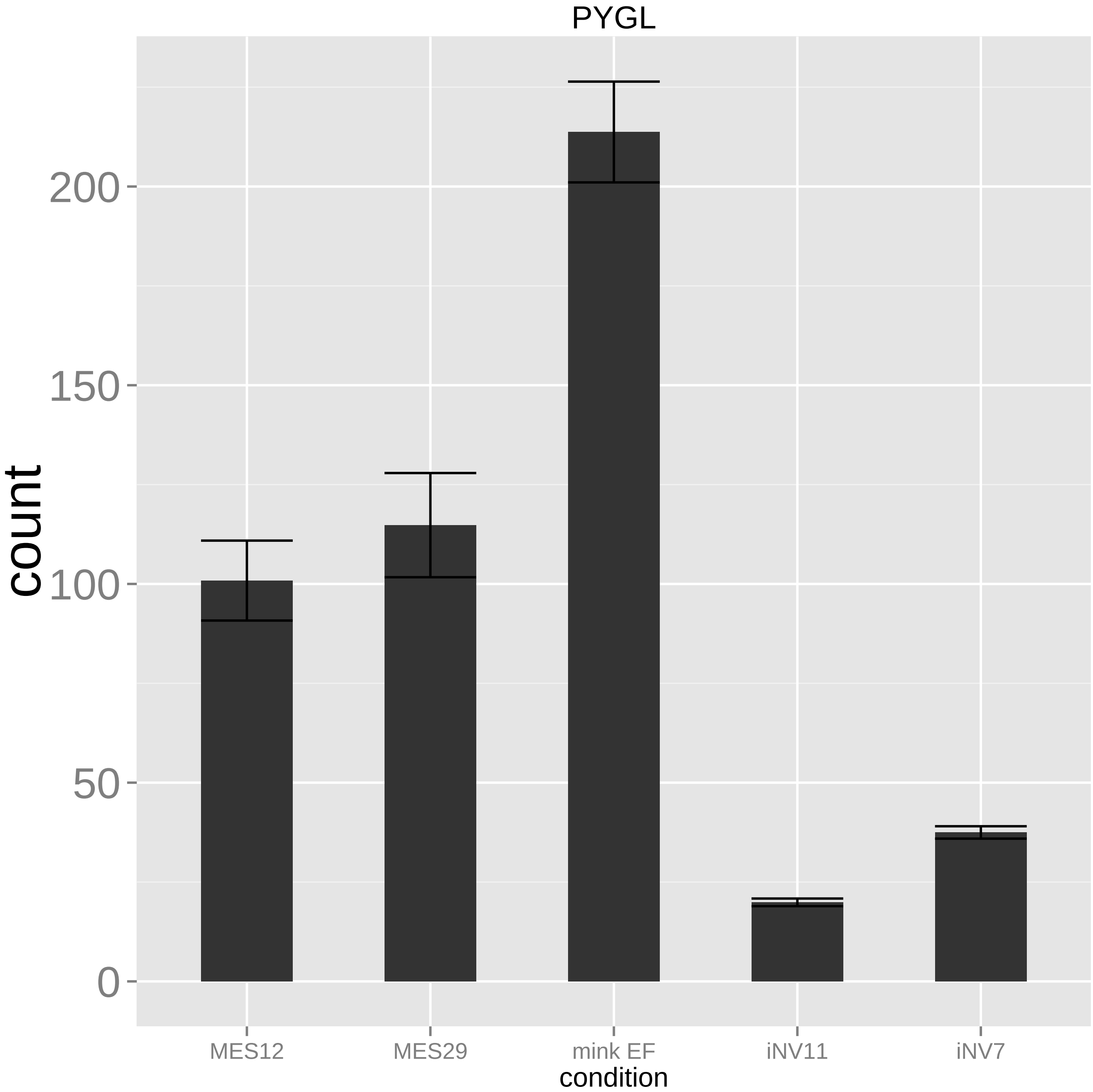
PYGL
count
200
150
100
50
0
MES12
MES29
mink EF
iNV11
iNV7
condition

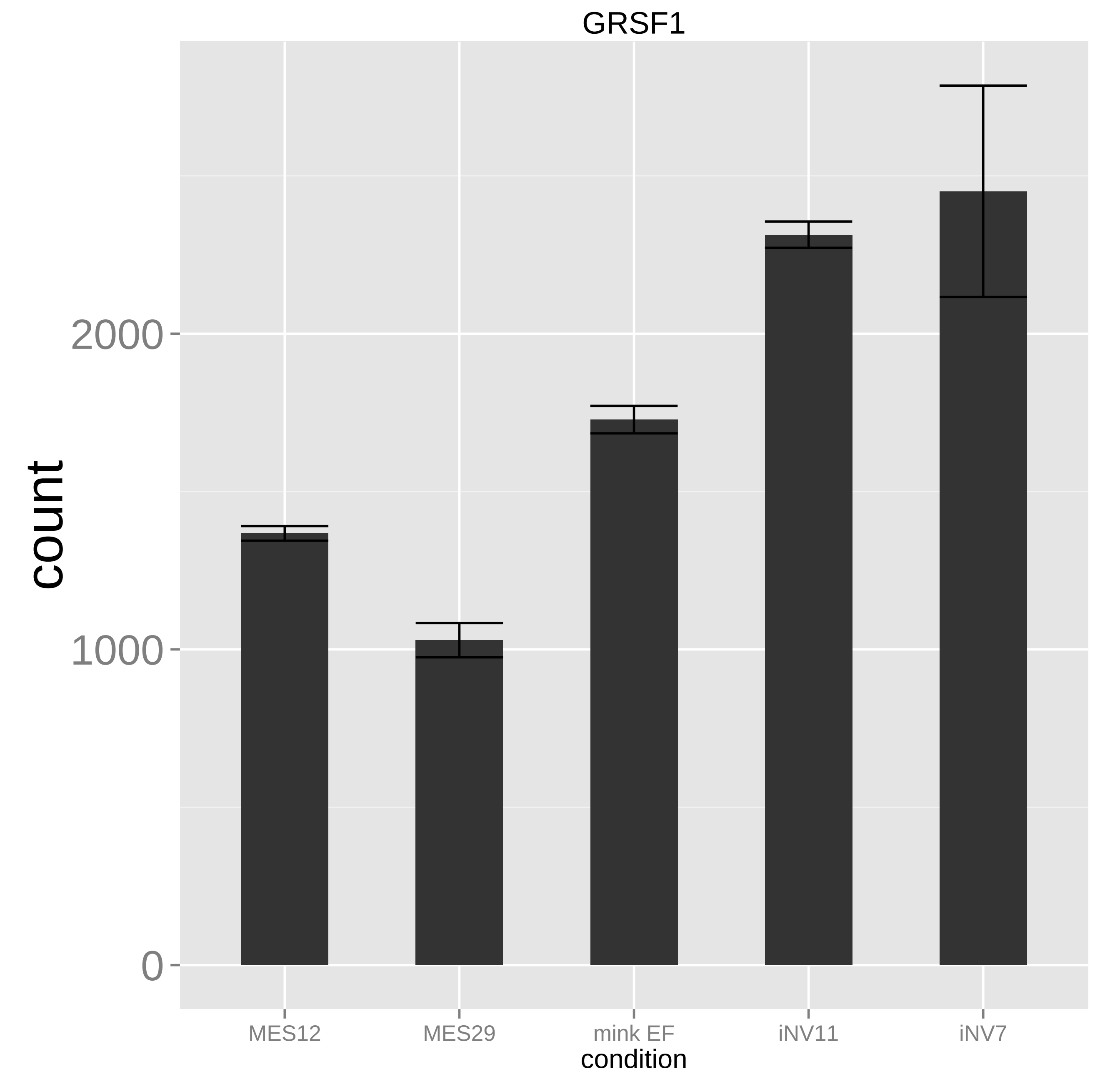
GRSF1
count
2000
1000
0
MES12
MES29
mink EF
iNV11
iNV7
condition

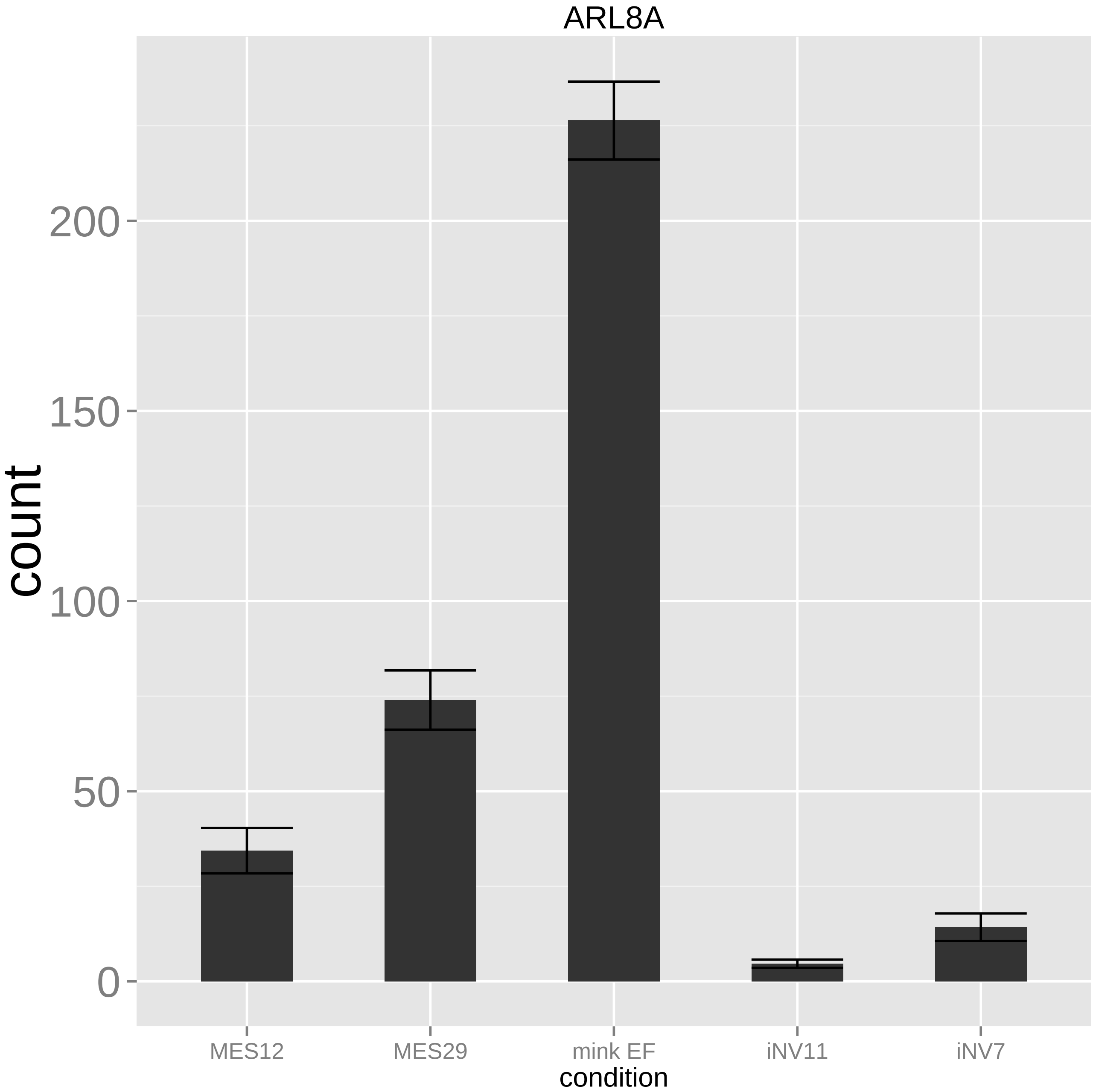
ARL8A
count
200
150
100
50
0
MES12
MES29
mink EF
iNV11
iNV7
condition

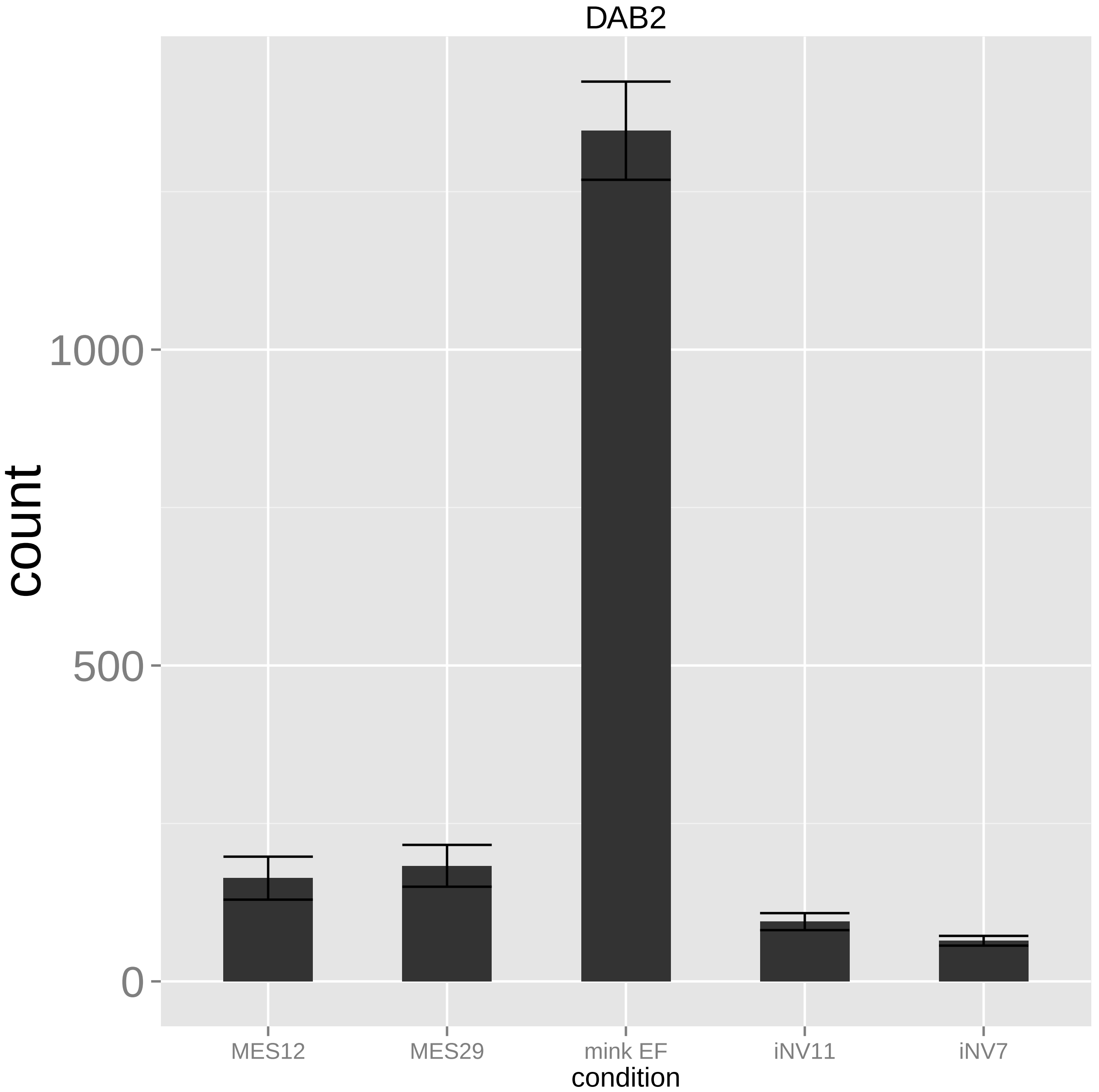
DAB2
count
1000
500
0
MES12
MES29
mink EF
iNV11
iNV7
condition

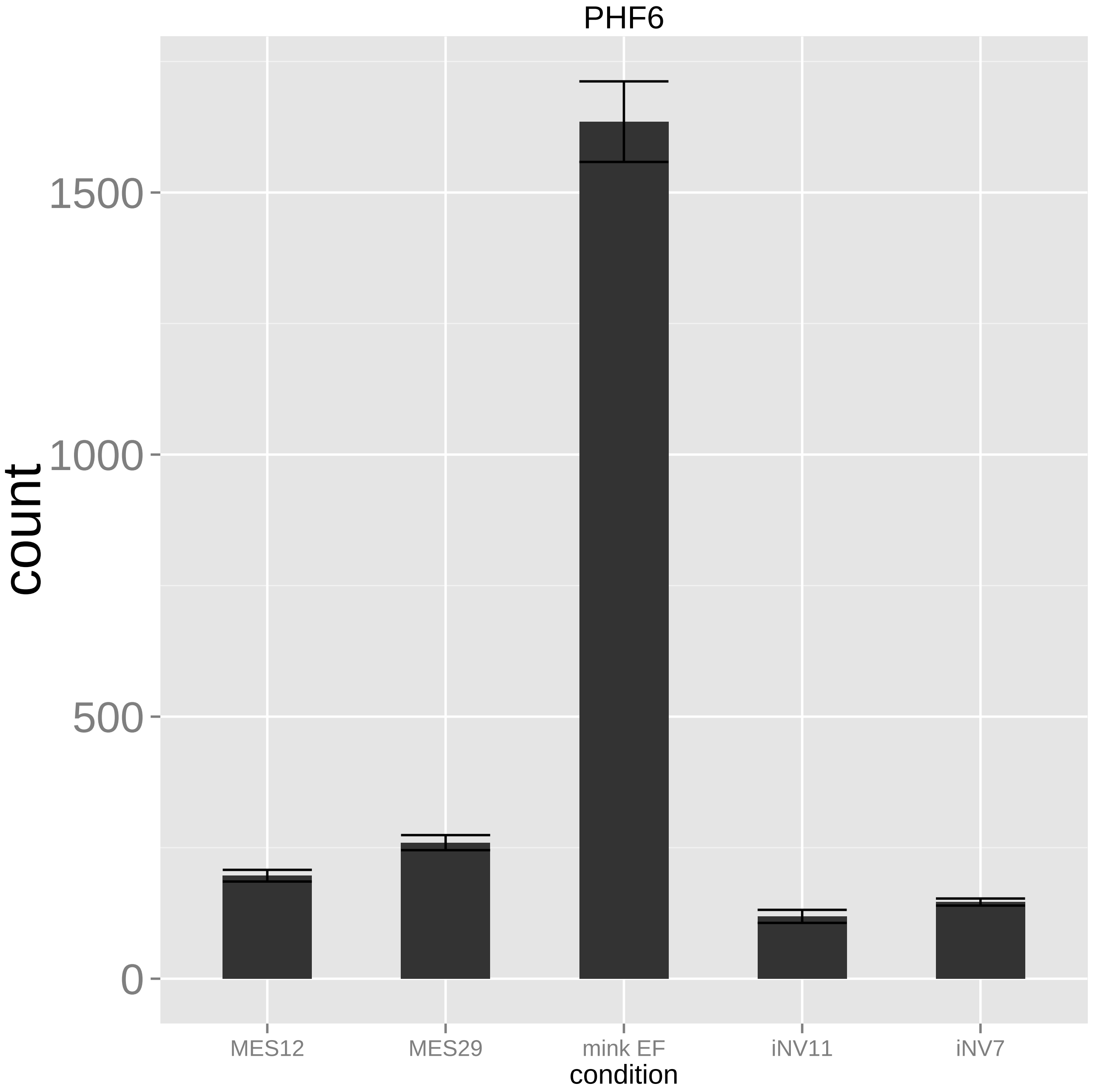

PHF6
count
1500
1000
500
0
MES12
MES29
mink EF
iNV11
iNV7
condition

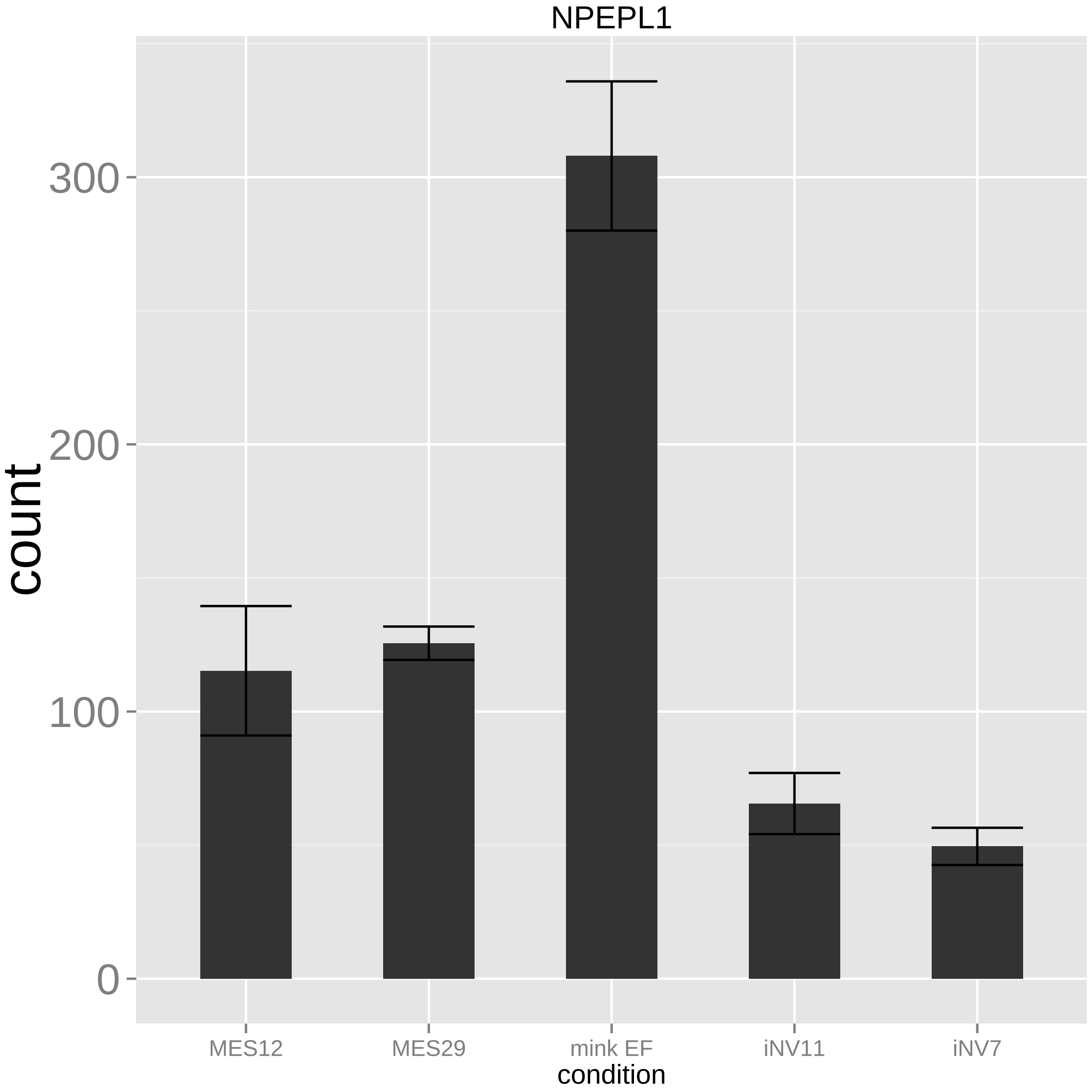
NPEPL1
count
300
200
100
0
MES12
MES29
mink EF
iNV11
iNV7
condition

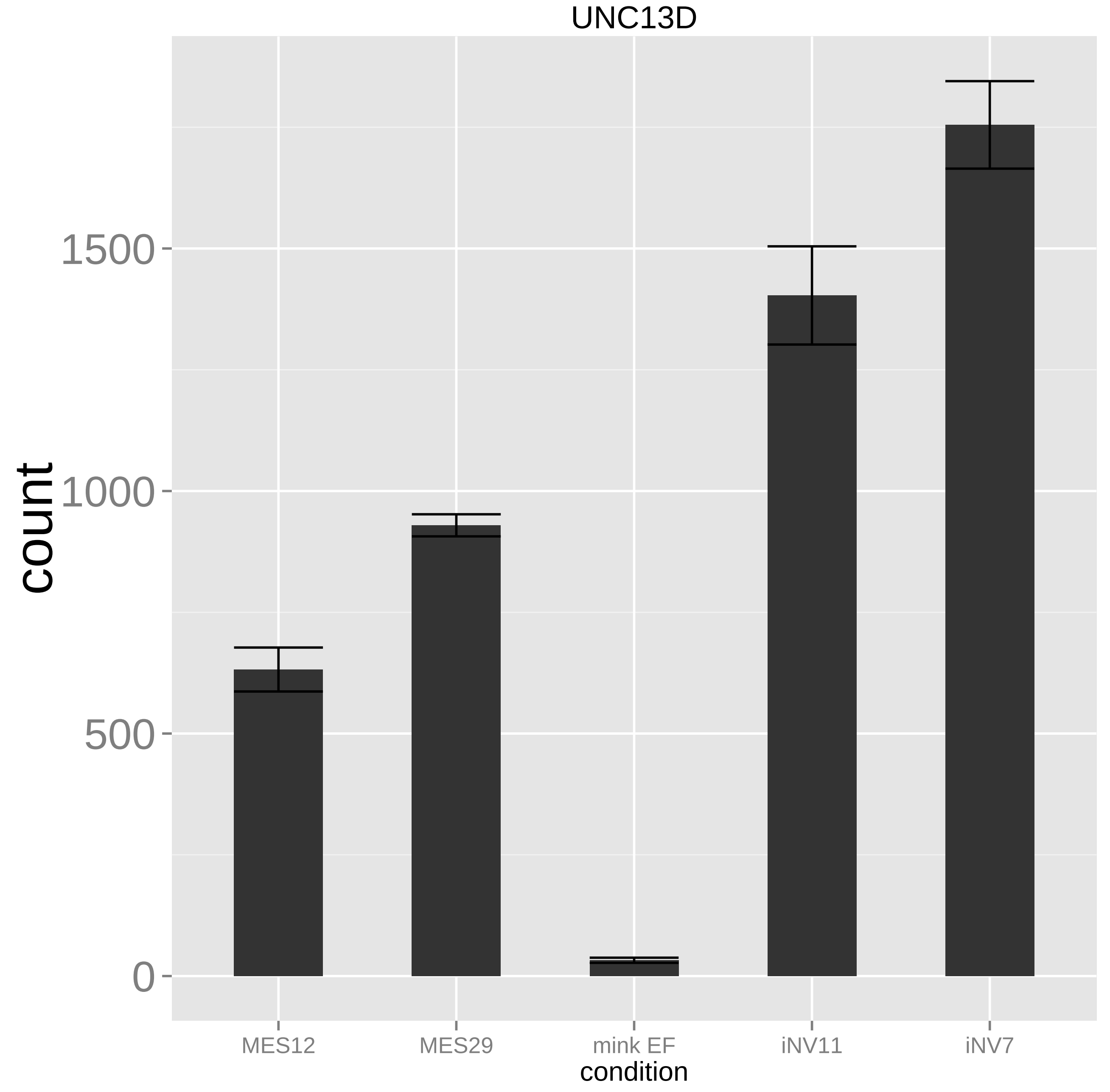

UNC13D
count
1500
1000
500
0
MES12
MES29
mink EF
iNV11
iNV7
condition

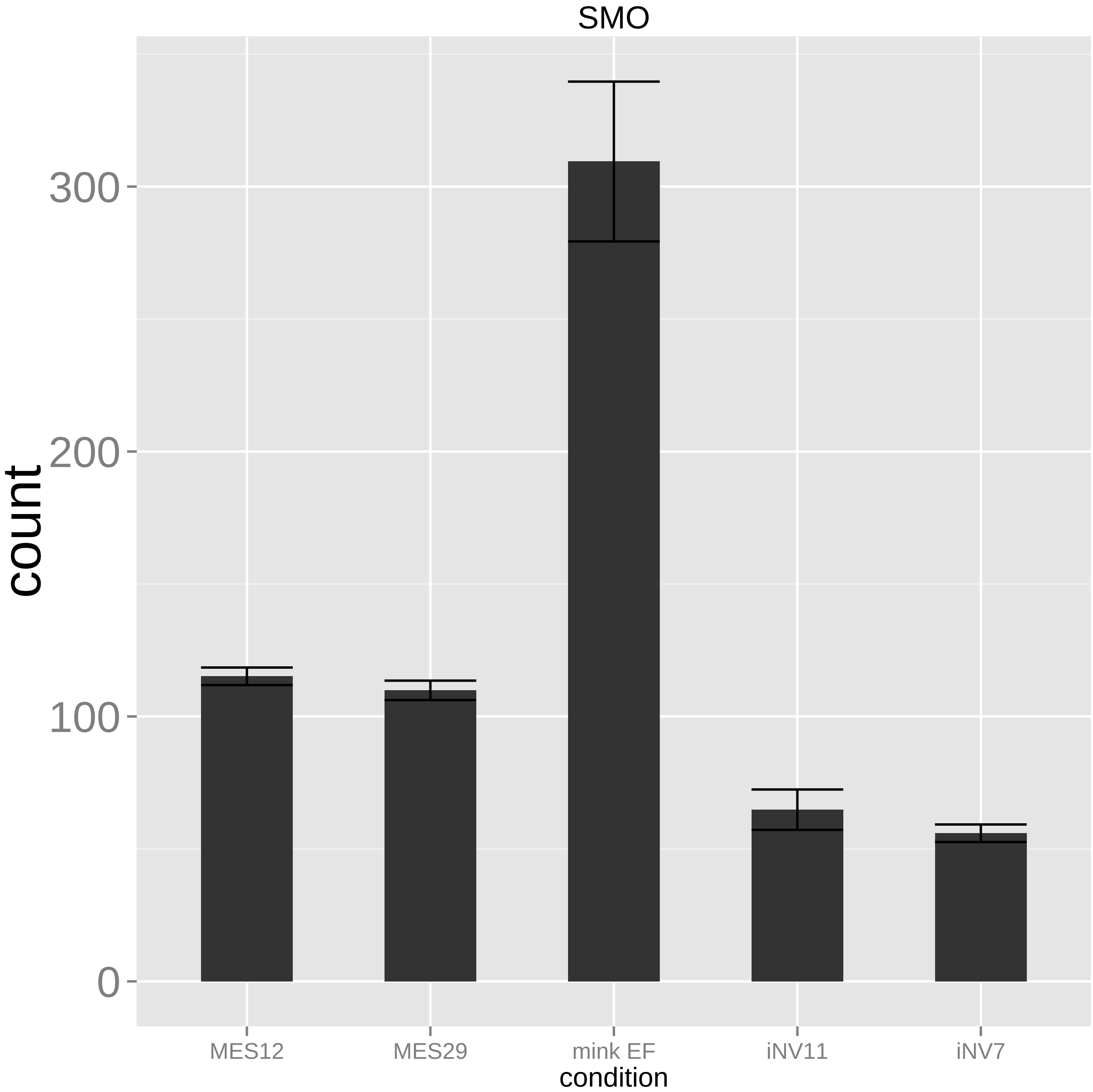

SMO
count
300
200
100
0
MES12
MES29
mink EF
iNV11
iNV7
condition

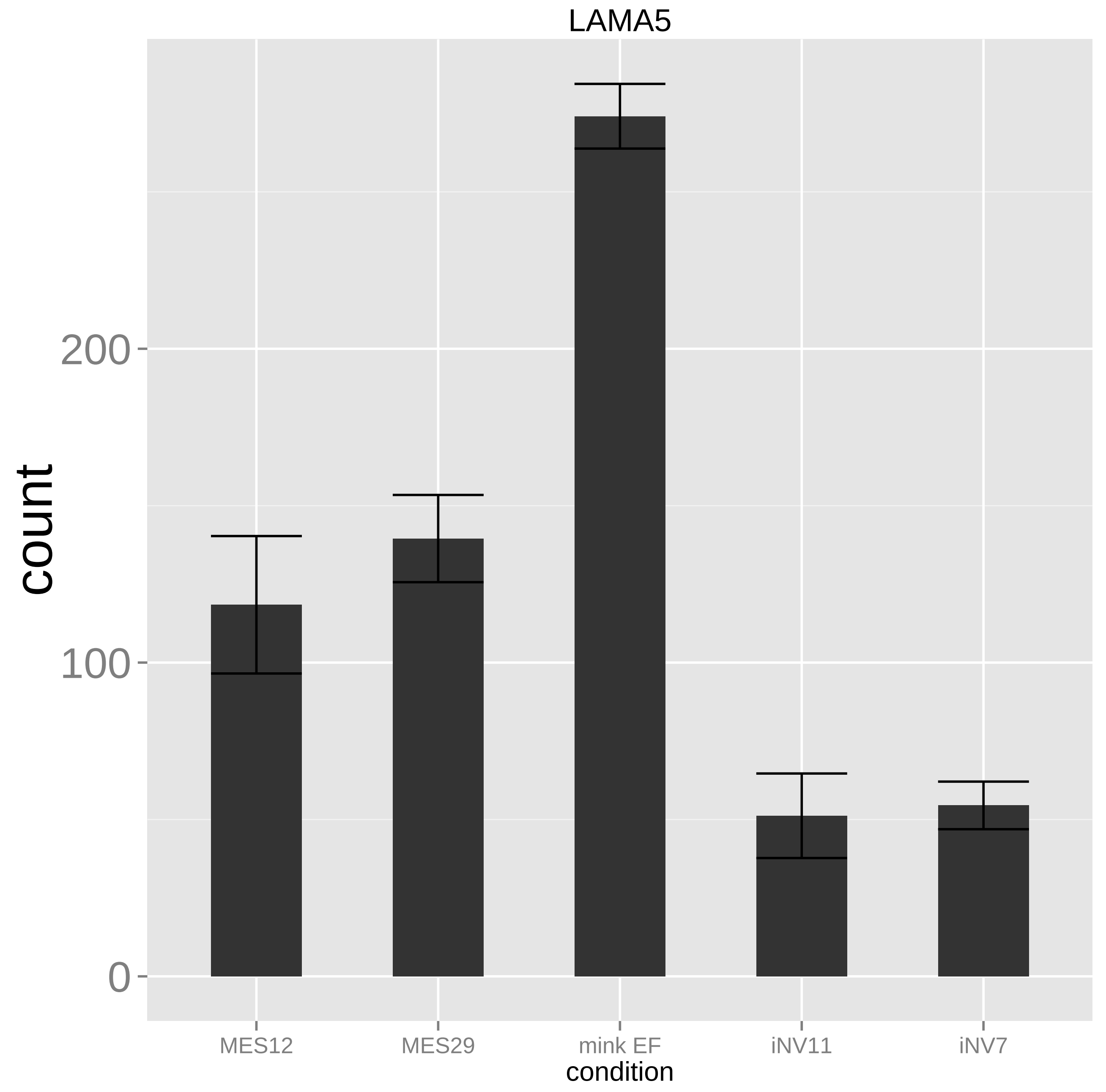
LAMA5
count
200
100
0
MES12
MES29
mink EF
iNV11
iNV7
condition

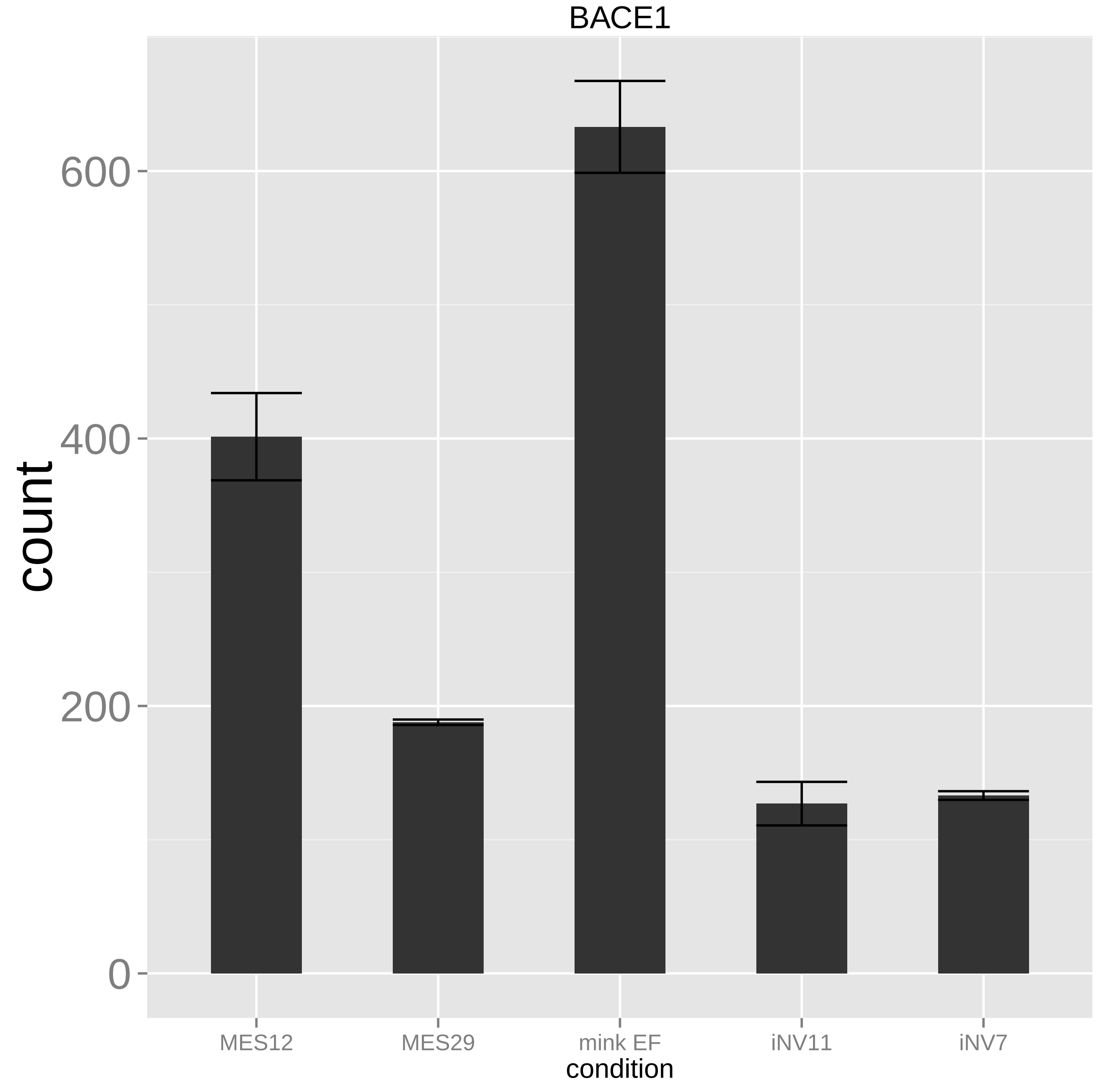
BACE1
count
600
400
200
0
MES12
MES29
mink EF
iNV11
iNV7
condition

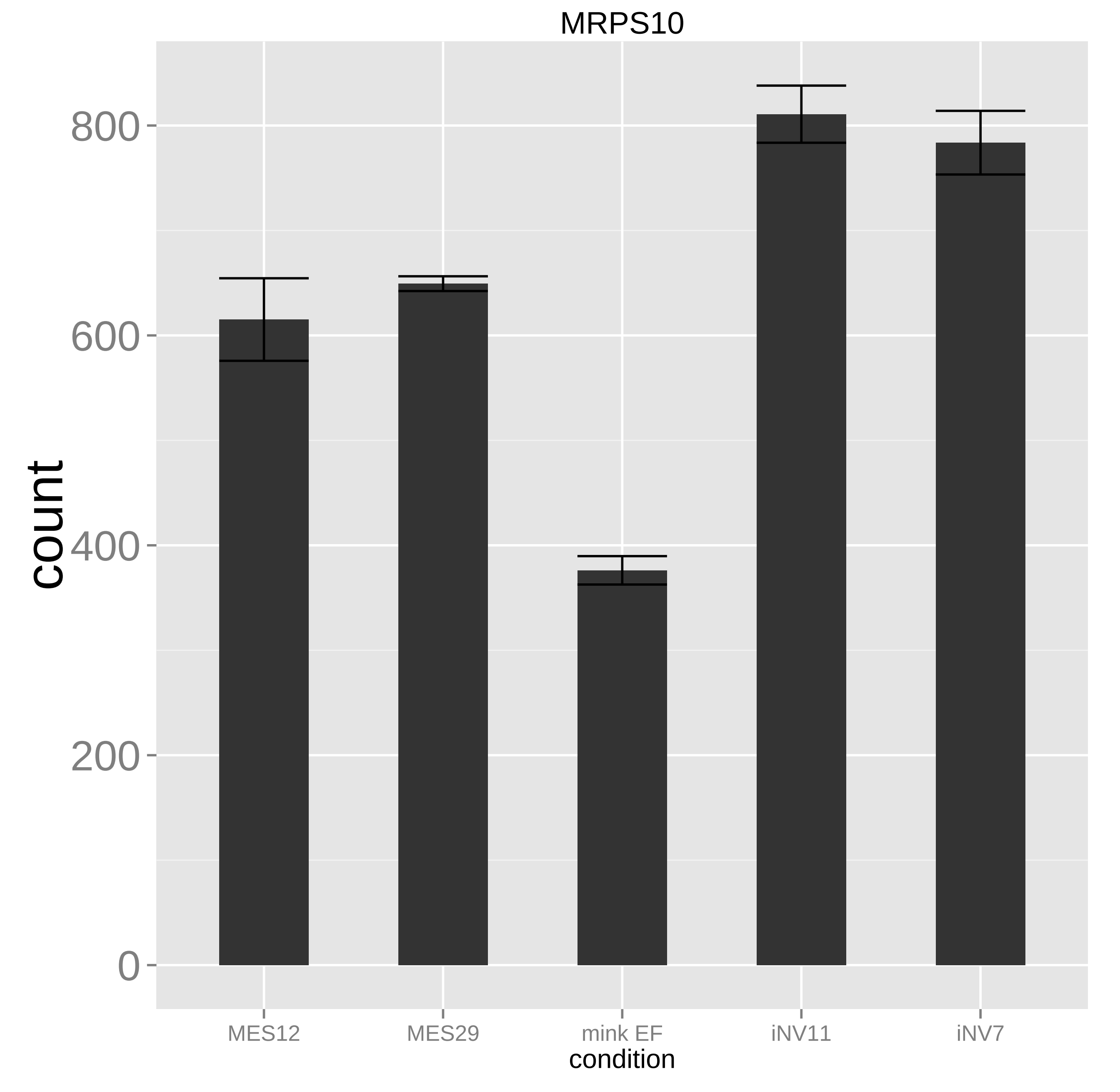
MRPS10
count
800
600
400
200
0
MES12
MES29
mink EF
iNV11
iNV7
condition

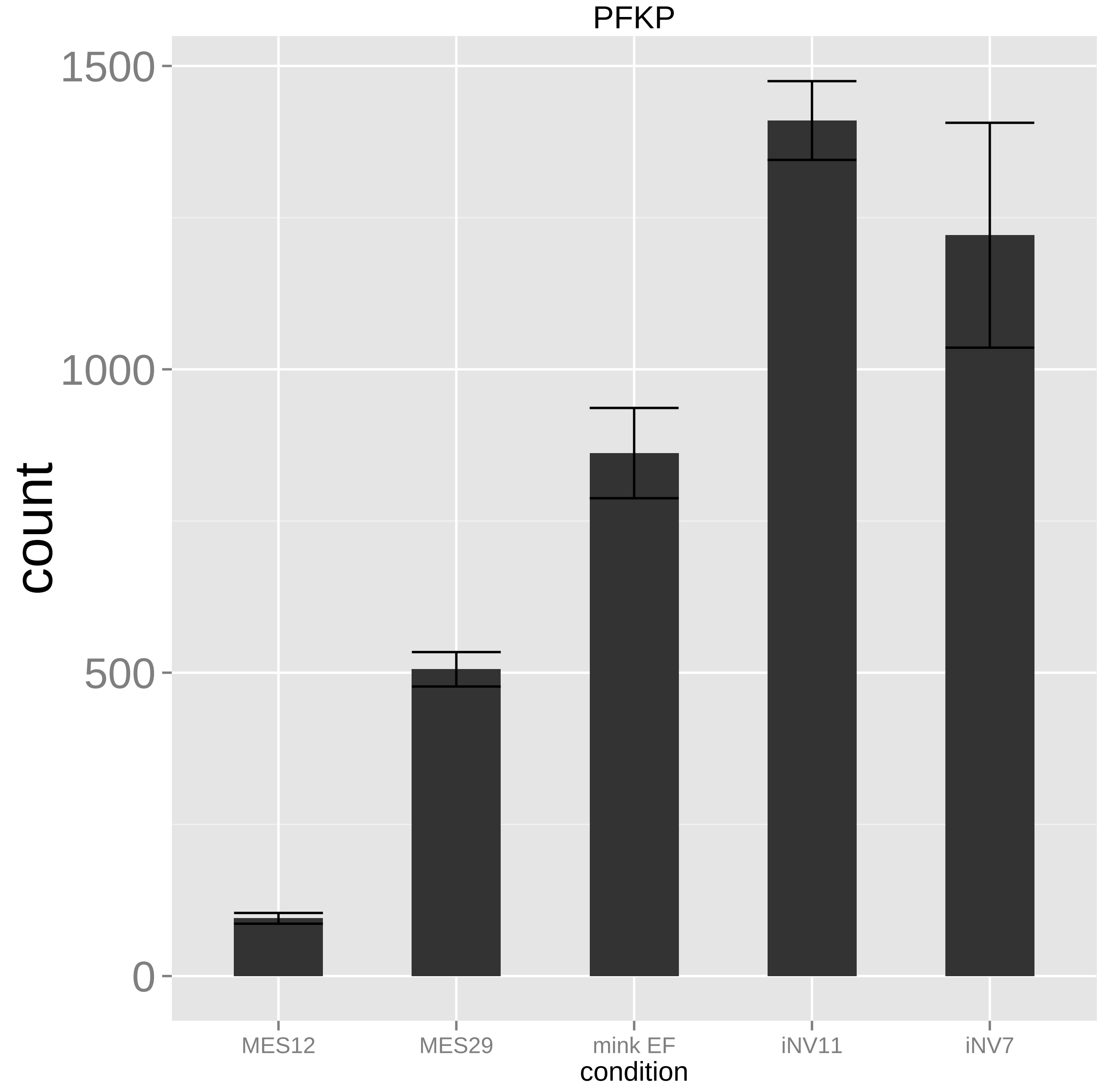
PFKP
count
1500
1000
500
0
MES12
MES29
mink EF
iNV11
iNV7
condition

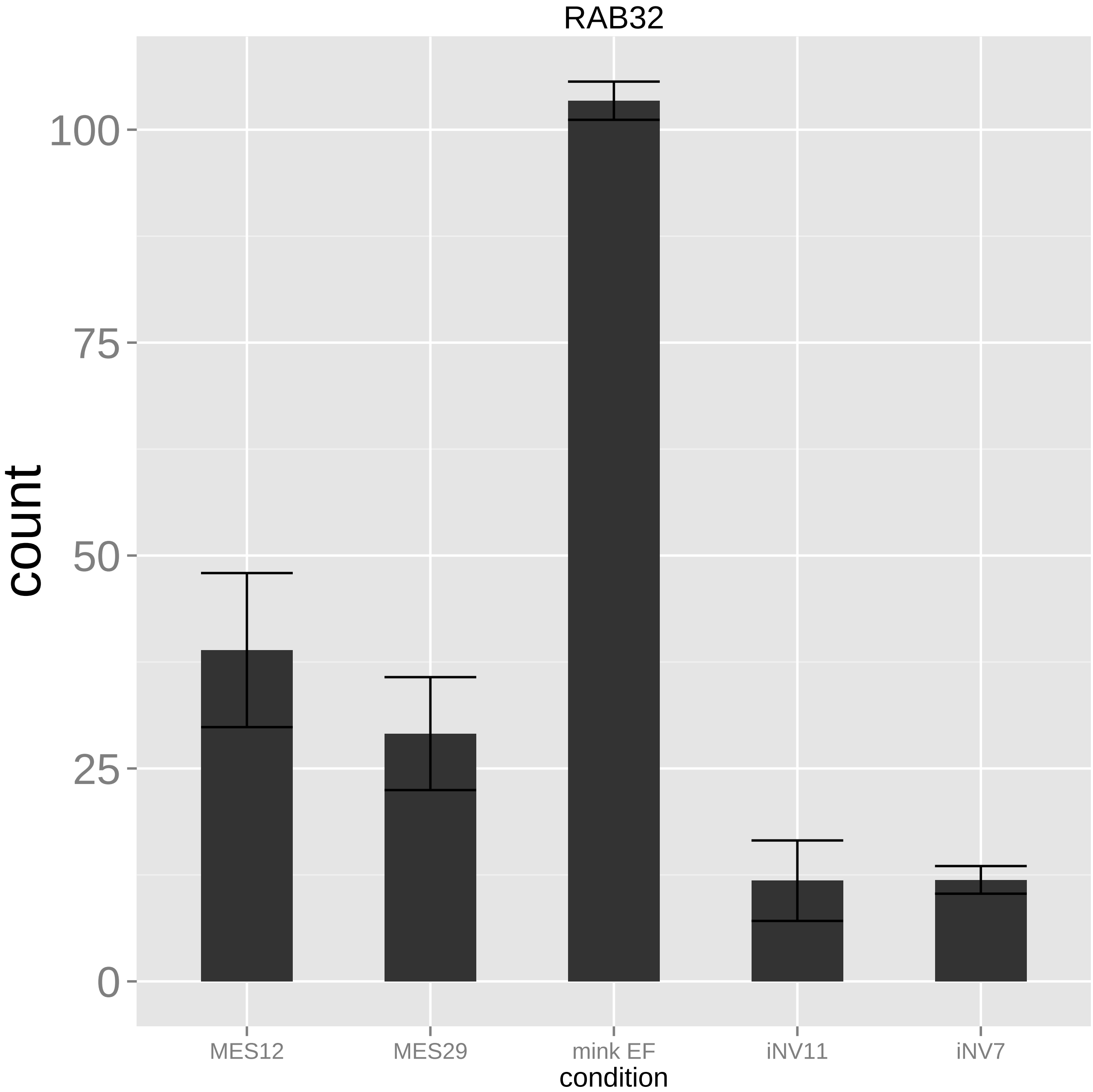
RAB32
count
100
75
50
25
0
MES12
MES29
mink EF
iNV11
iNV7
condition

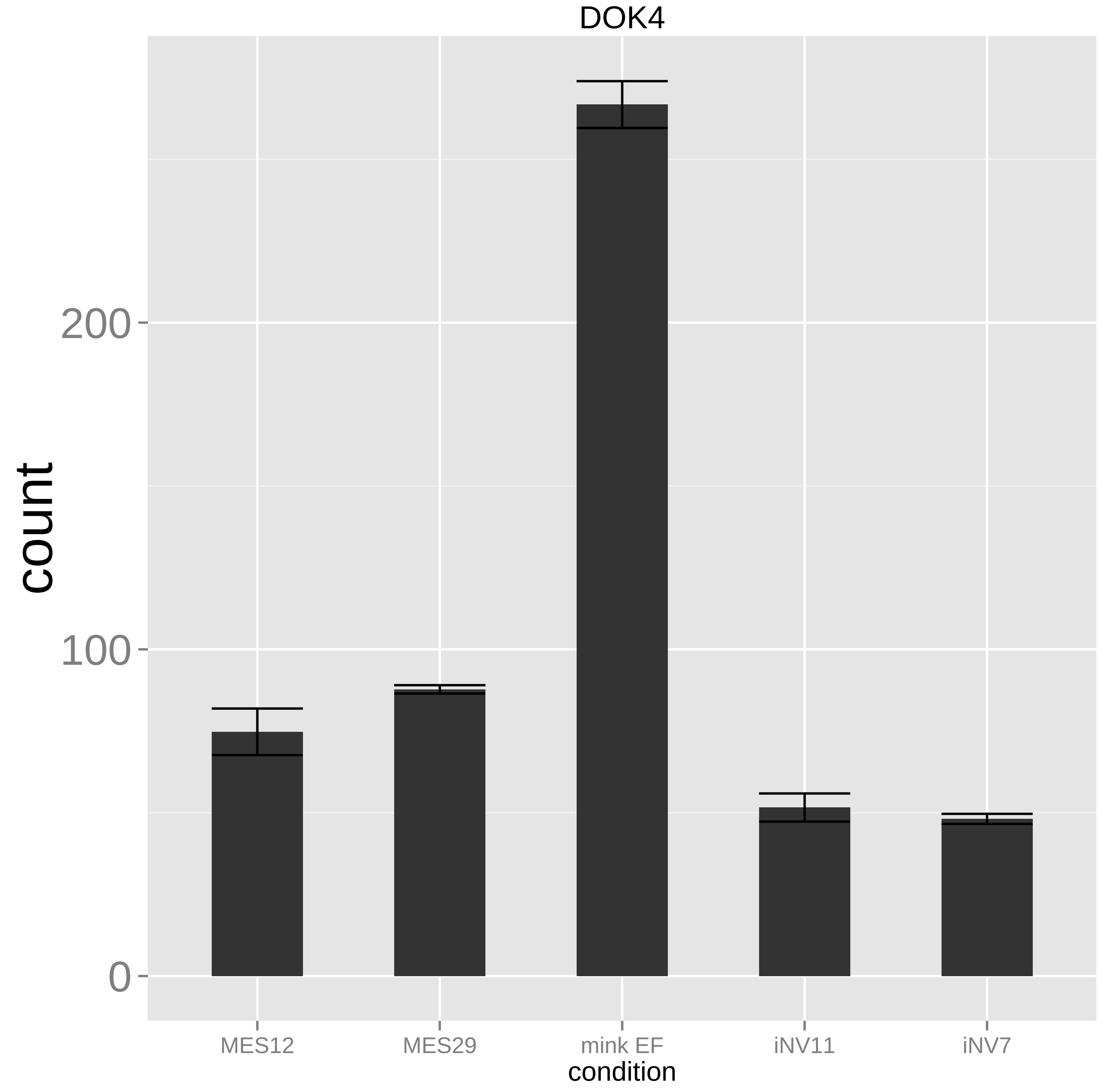

DOK4
count
200
100
0
MES12
MES29
mink EF
iNV11
iNV7
condition

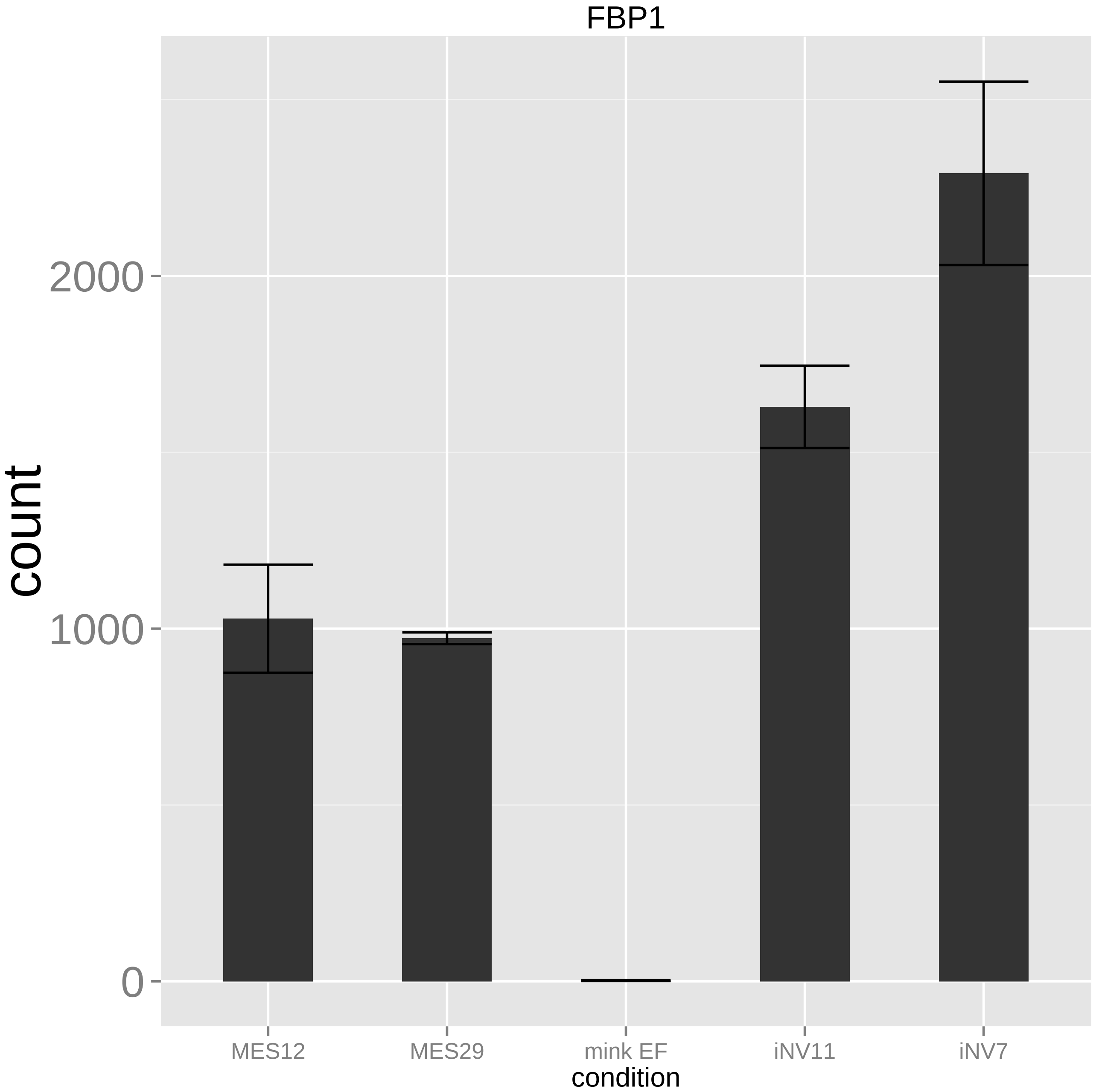
FBP1
count
2000
1000
0
MES12
MES29
mink EF
iNV11
iNV7
condition

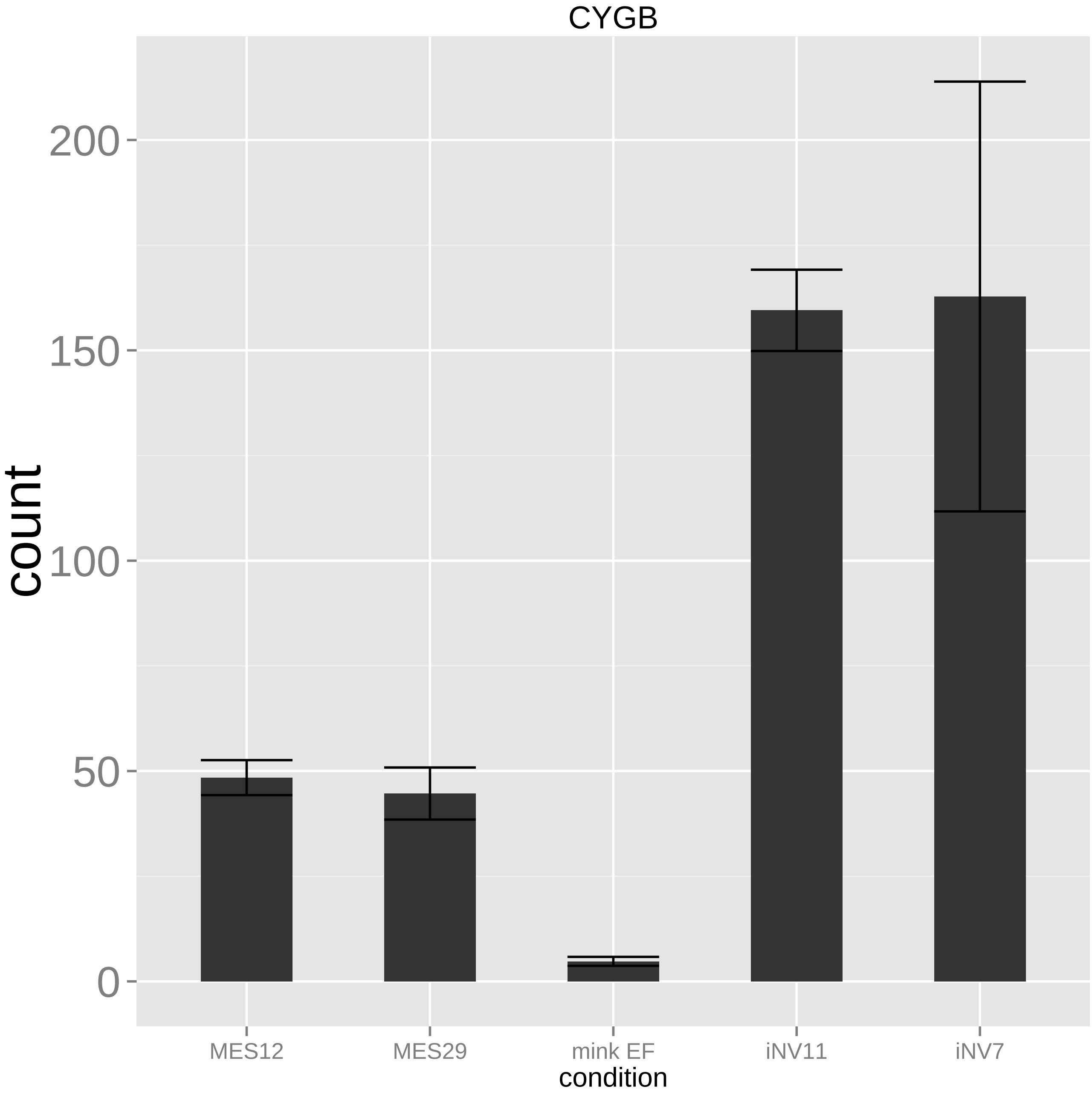

CYGB
count
200
150
100
50
0
MES12
MES29
mink EF
iNV11
iNV7
condition

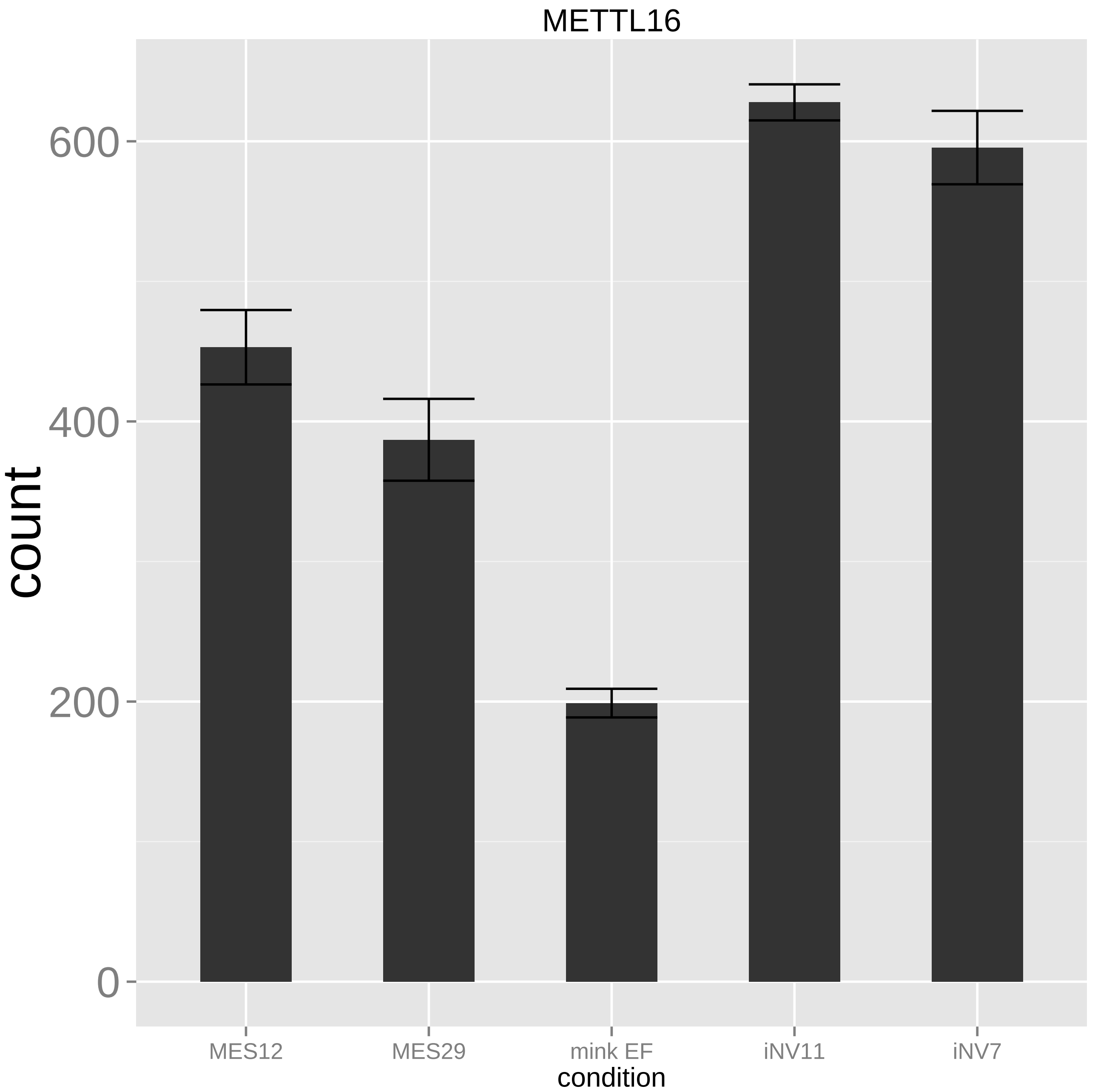

METTL16
600
400
200
0
count
MES12
MES29
mink EF
iNV11
iNV7
condition

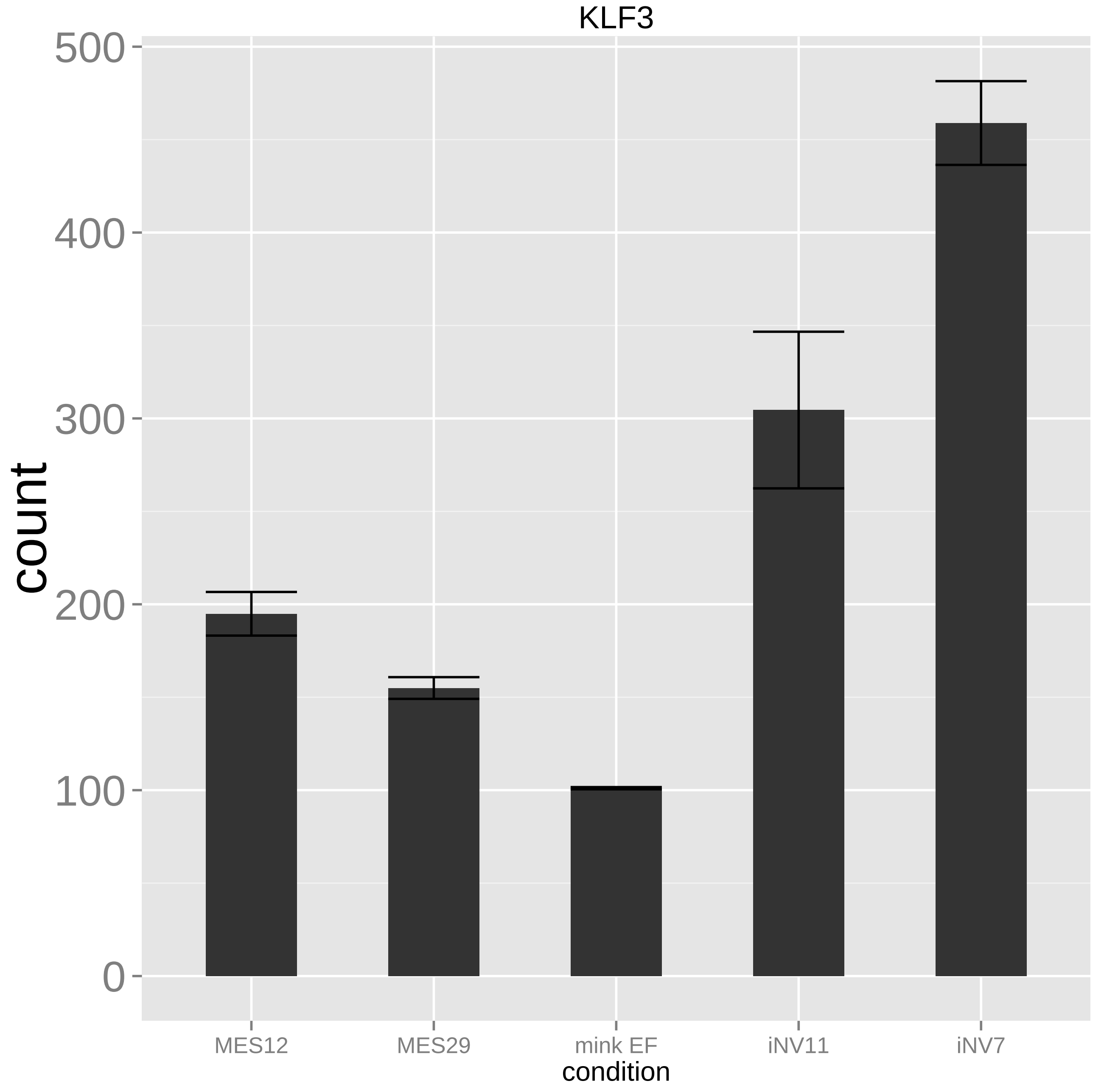
KLF3
count
500
400
300
200
100
0
MES12
MES29
mink EF
iNV11
iNV7
condition

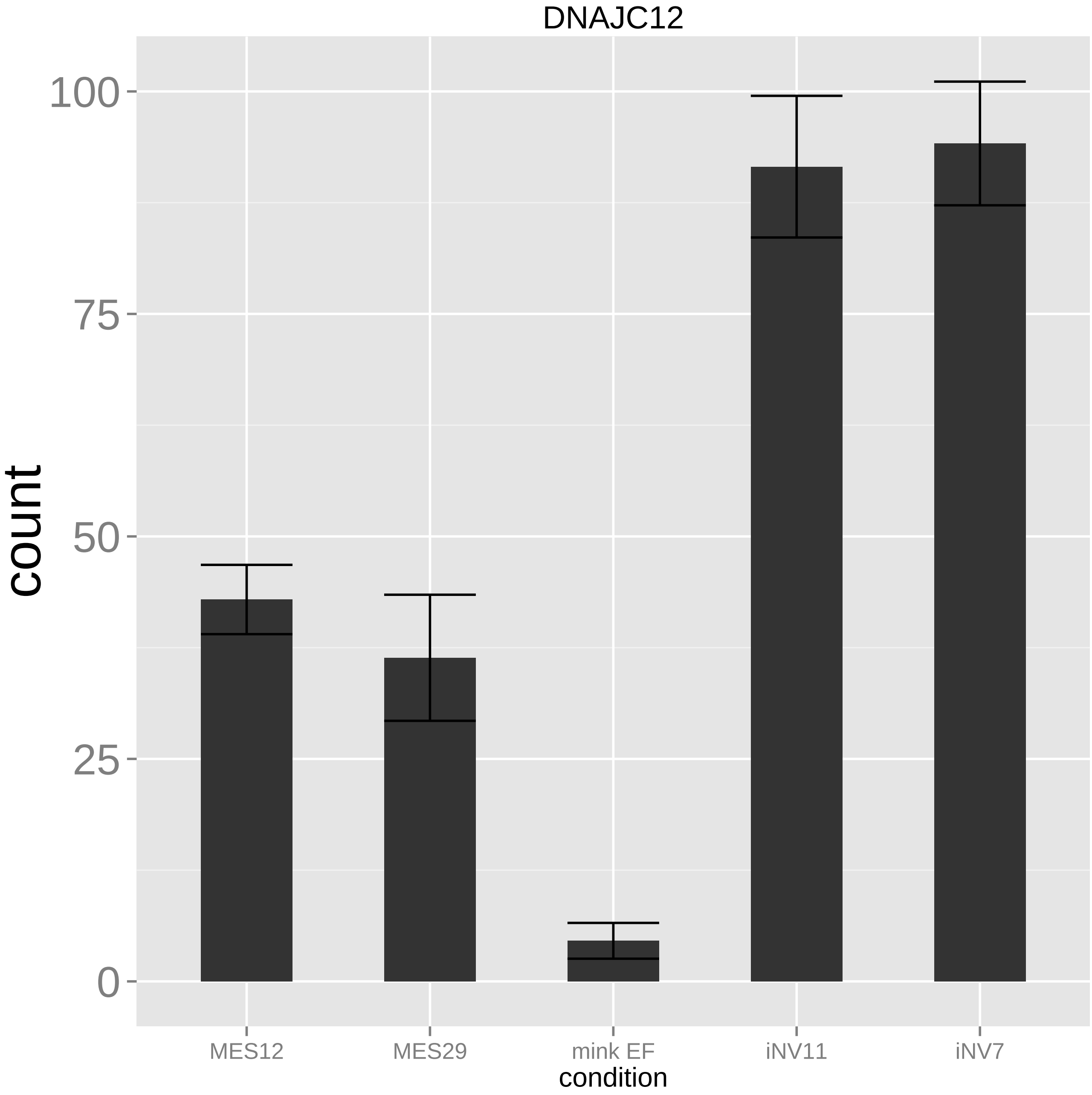
DNAJC12
count
100
75
50
25
0
MES12
MES29
mink EF
iNV11
iNV7
condition

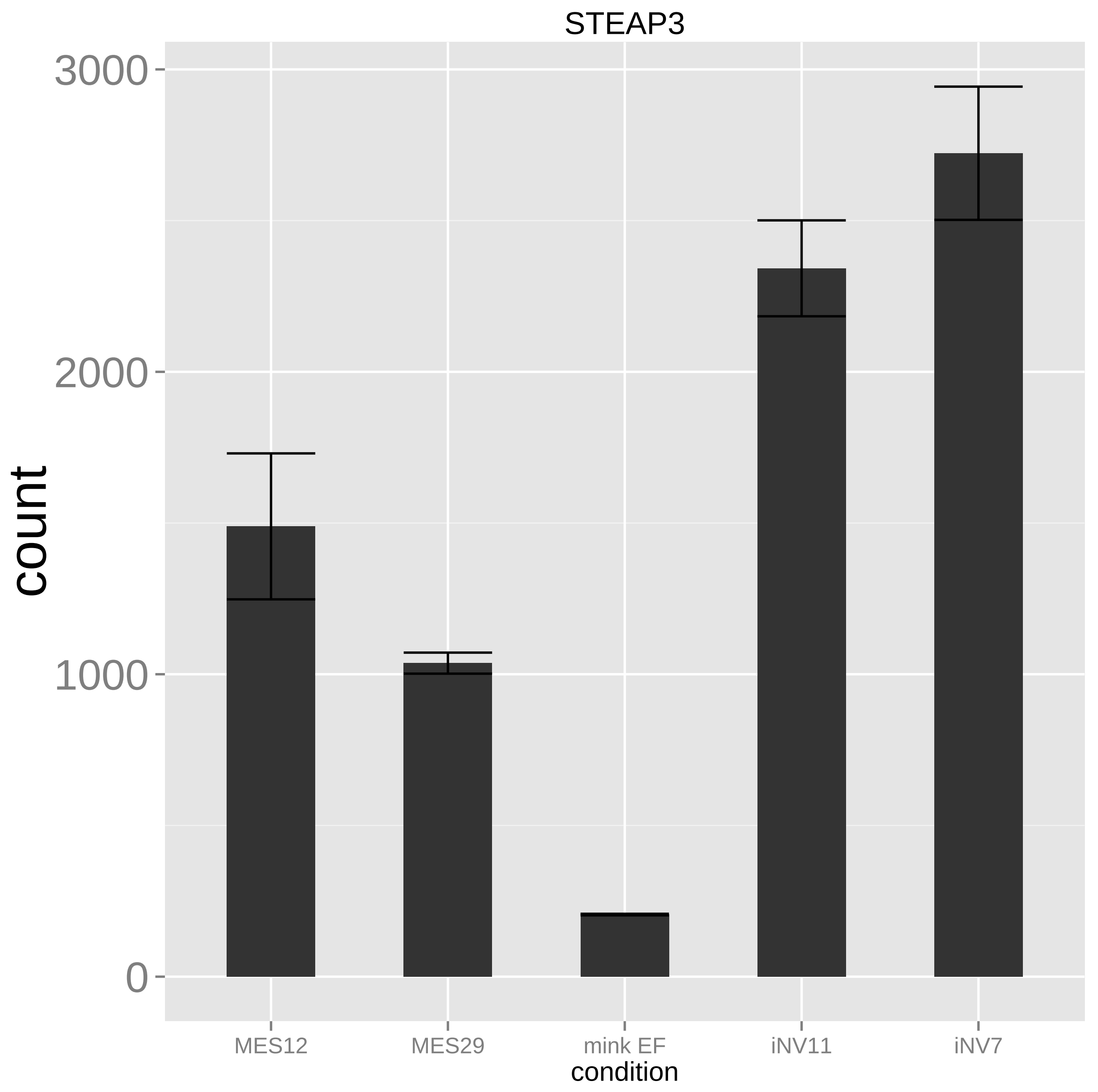
STEAP3
count
3000
2000
1000
0
MES12
MES29
mink EF
iNV11
iNV7
condition

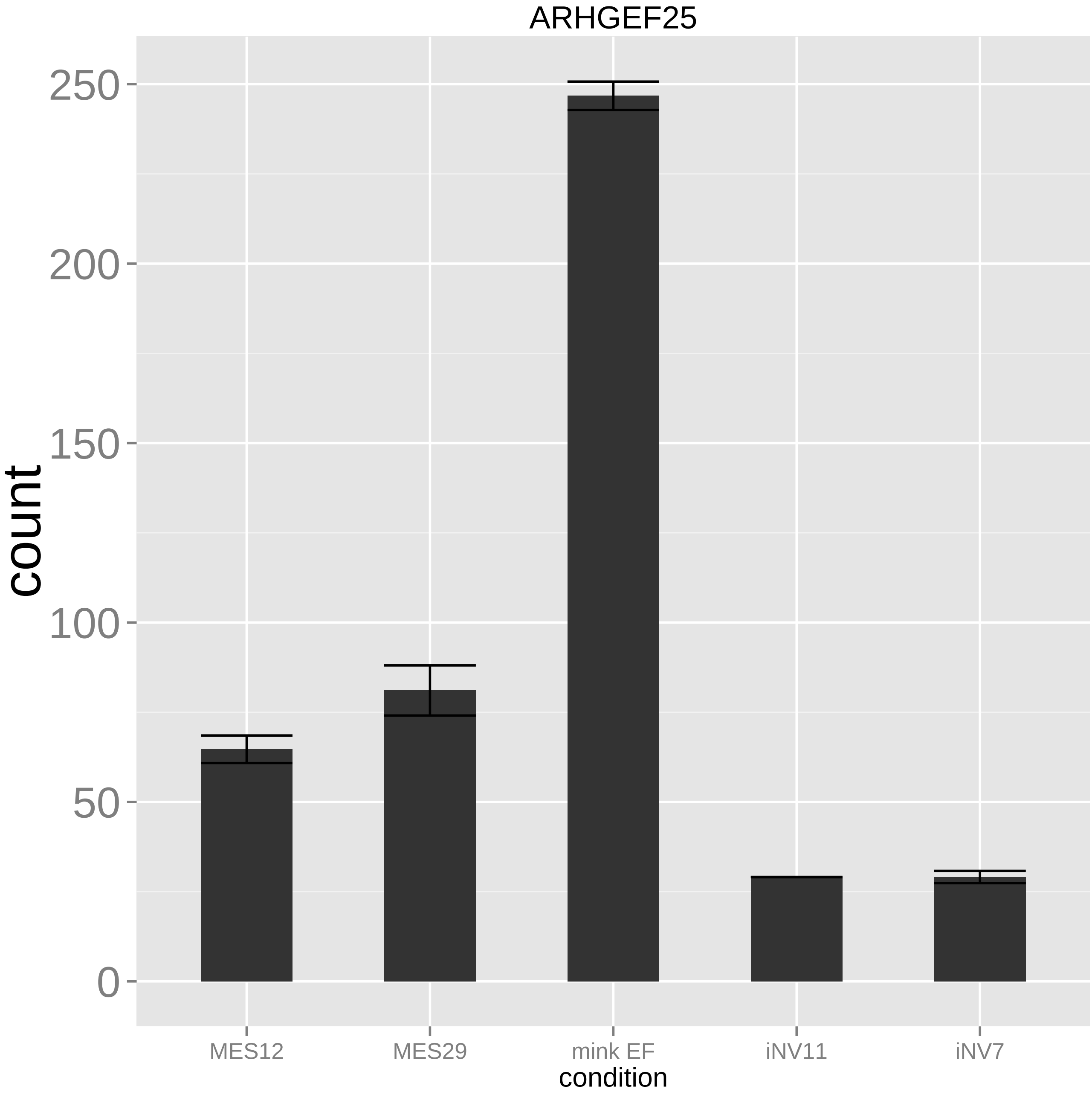
ARHGEF25
count
250
200
150
100
50
0
MES12
MES29
mink EF
iNV11
iNV7
condition

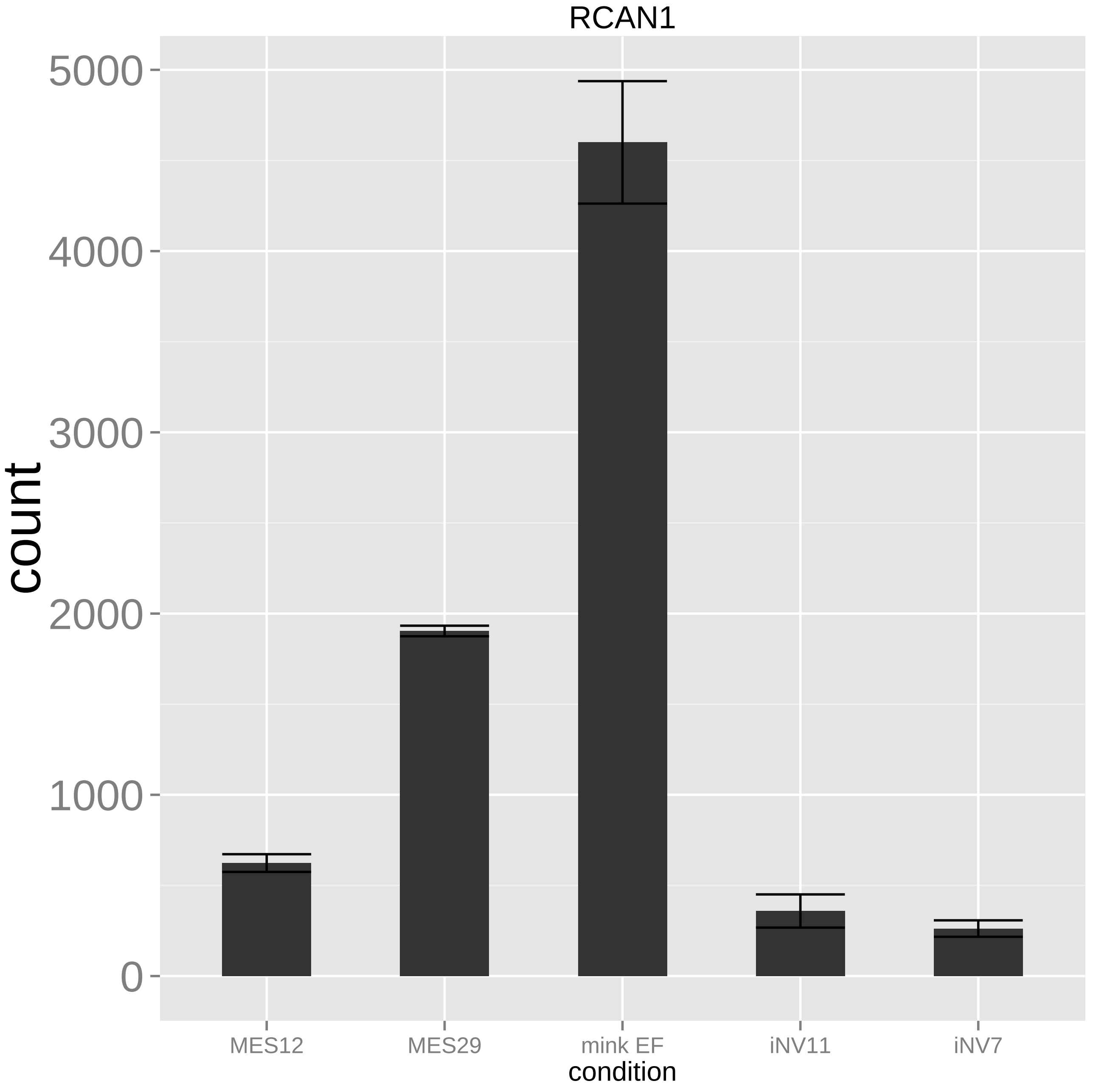
RCAN1
count
5000
4000
3000
2000
1000
0
MES12
MES29
mink EF
iNV11
iNV7
condition

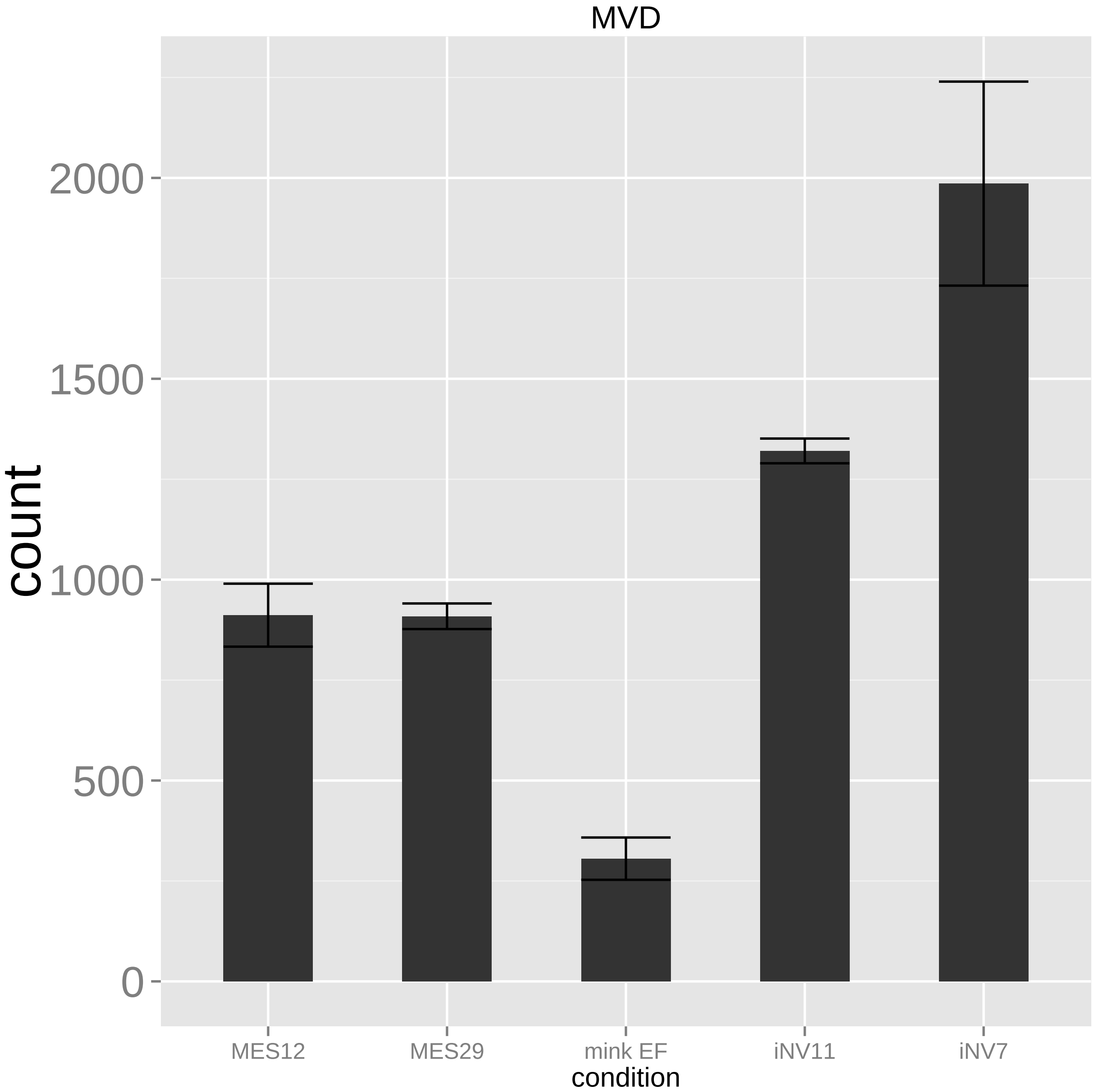
MVD
count
2000
1500
1000
500
0
MES12
MES29
mink EF
iNV11
iNV7
condition

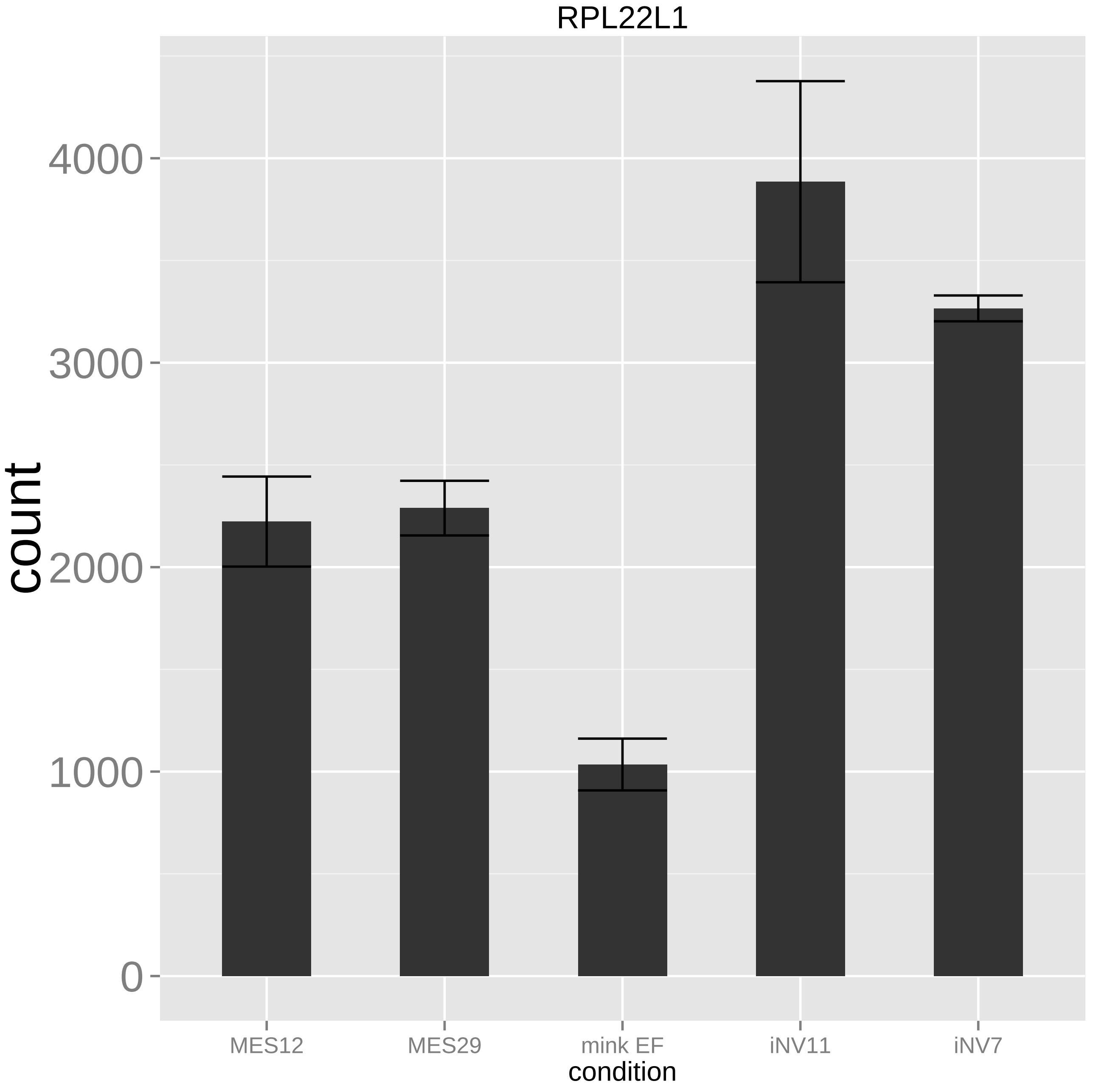
RPL22L1
count
4000
3000
2000
1000
0
MES12
MES29
mink EF
iNV11
iNV7
condition
